# Facile Synthesis of Carbamoyl Fluorides *viaN*-Carbamoylimidazole Activation

**DOI:** 10.1021/acsomega.4c09438

**Published:** 2025-02-14

**Authors:** Anže Meden, Damijan Knez, Stanislav Gobec

**Affiliations:** University of Ljubljana, Faculty of Pharmacy, Department of Pharmaceutical Chemistry, Aškerčeva 7, SI-1000 Ljubljana, Slovenia

## Abstract

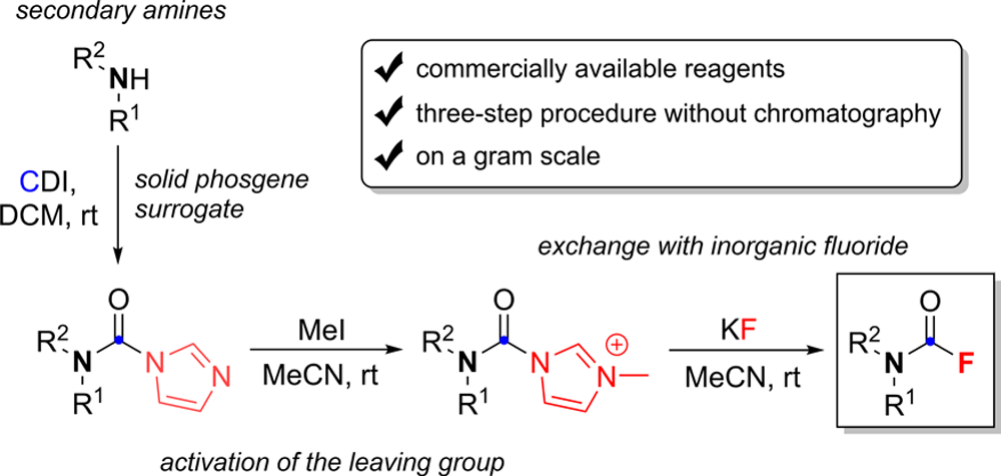

The untapped potential of carbamoyl fluorides for various chemico/biological applications is hampered by the scarcity of straightforward and benign methods for their synthesis. In this report, we disclose a novel mild three-step procedure that avoids exotic, corrosive, and highly toxic reagents. Briefly, commercially available secondary amines are carbamoylated with 1,1′-carbonyldiimidazole, followed by alkylation to improve nucleofugality, and exchange with inorganic KF. This procedure works on a gram scale without chromatographic purification. It is however limited to basic, sterically unhindered secondary amines without alkylation-prone functional groups.

## 1 Introduction

Carbamoyl fluorides (CFs) are an interesting class of underexplored organofluorine compounds with great potential for various biology- and medicinal chemistry-related uses.^1^ Nevertheless, of 963 references in SciFinder database (August 2024) containing NC(=O)F motif, only 47 were in biological studies. Most of these refer to simple CFs (e.g., dimethylcarbamoyl fluoride) which have been identified as covalent inhibitors of cholinesterases since the seminal paper by Myers and Kemp in 1954.^2−5^ Although reactive electrophiles, CFs were reported to be sufficiently stable in organic solvents, water, and aqueous buffers of acidic or neutral pH, even in the presence of nucleophilic amino acids or albumin.^1^ CFs are useful synthetic intermediates, as well–for instance, in synthesis of ureas (thio-, seleno-)carbamates, amides,^6−8^ and for (cross-)coupling reactions.^9−14^ Therefore, development of new methods for synthesis of CFs is currently a hot topic in the research community.

**Scheme 1:**
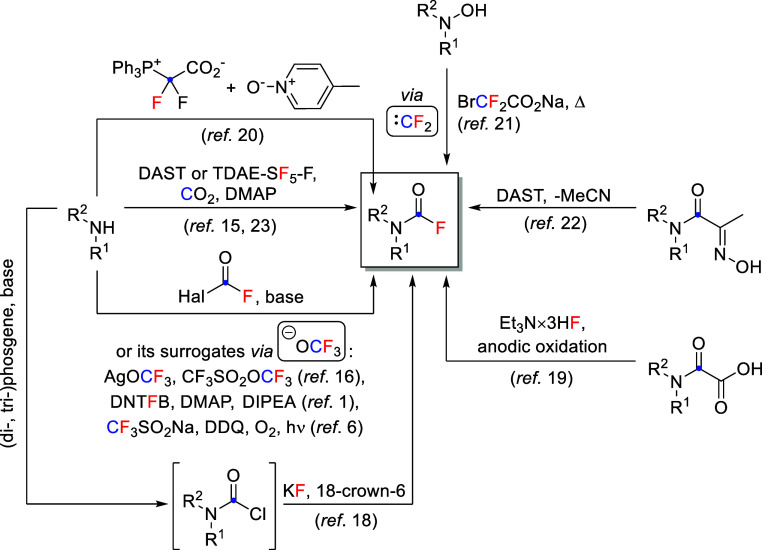
Different Methods for CF Synthesis If R^1^ or R^2^ = H, compounds easily decompose with elimination of HF.

A plethora of different methods were reported for synthesis of CFs, differing in functional group tolerability, substrate scope, convenience, use of exotic, toxic, explosive, gaseous reagents, heavy metals, etc (Scheme 1).^15−18^ Some recent examples include anodic oxidation of oxamic acids,^19^ pyridine-*N*-oxide- or hydroxylamine-mediated oxidation of an in situ-generated difluorocarbene,^20,21^ fluorinative Beckmann fragmentation of α-oximinoamides,^22^ carbon dioxide deoxyfluorination using SF_6_-derived reagent,^23^ and a dinitrotrifluoromethoxybenzene-derived reagent.^1^ Moreover, CF_3_[S,Se]^−^-based methods exist for synthesis of thio- and selenoCFs.^24,25^ Meanwhile, secondary CFs can be prepared from (and also easily decompose back to) isocyanates.^26,27^

## 2 Results and Discussion

**Scheme 2:**
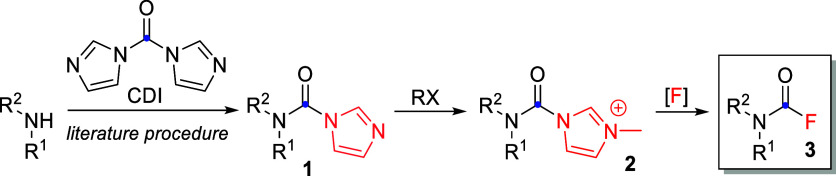
Our Method for Synthesis of Tertiary CFs

Certain carbamoyl fluorides were required as synthetic intermediates for our work on butyrylcholinesterase inhibitors. Inspired by the work of Batey group on carbamoylimidazolium salts,^28^ we serendipitously discovered that CFs can be formed under mild conditions from carbamoylimidazolium salts **2** in the presence of fluoride. Therefore, different fluoride sources and reaction conditions were screened (Table 1, entries 1–14), and mild reaction conditions–potassium fluoride in acetonitrile at room temperature–were found for the conversion of carbamoylimidazolium salts **2** to CFs **3** (Scheme 2). **2** themselves are obtained by alkylation of *N*-carbamoylimidazoles **1**, which are in turn facilely prepared from amines and 1,1′-carbonyldiimidazole (CDI)—a plethora of reported procedures exist in literature.^28,29^

**Table 1:**
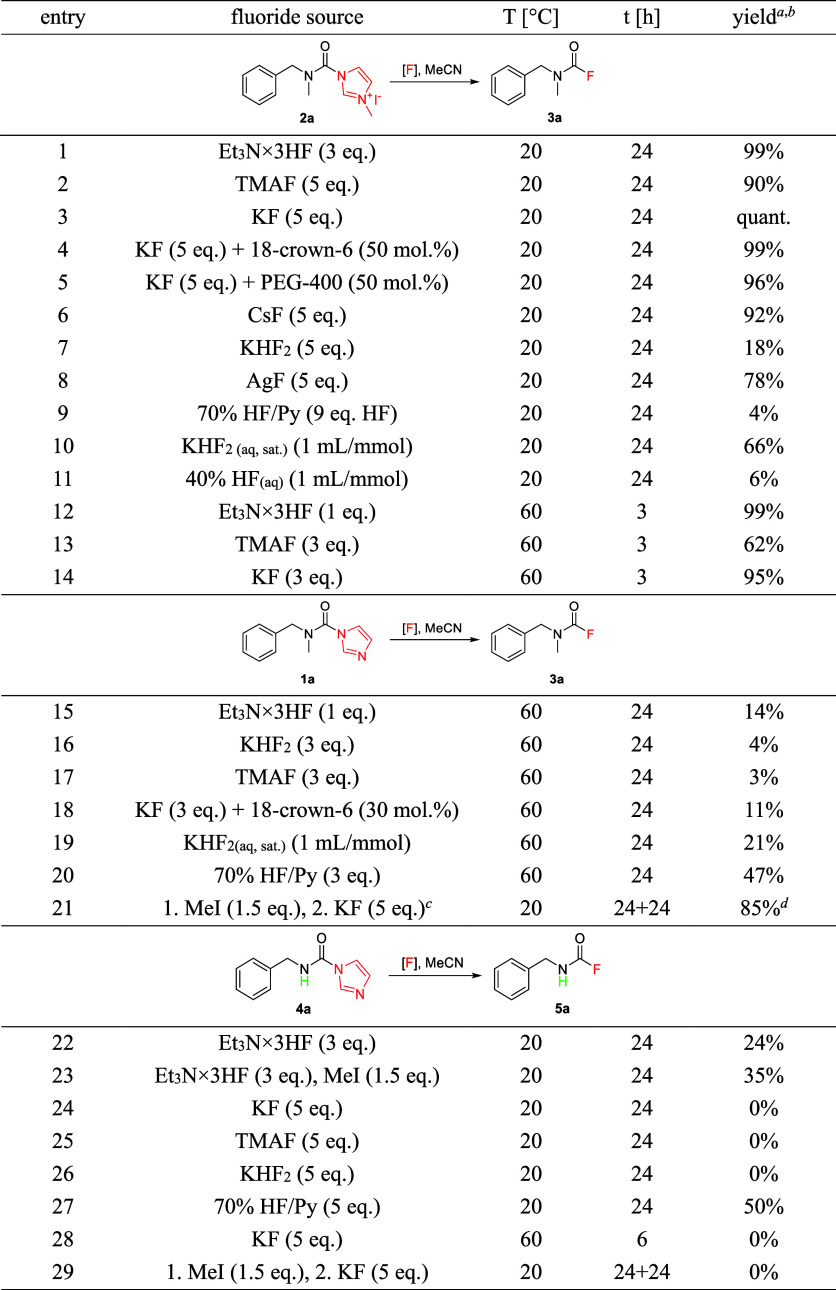
CF Synthesis−Screening of Reaction Conditions^a^,^b^,^c^,^d^

a Reaction conditions: 0.8–1.0 mmol, in MeCN (2 mL/mmol), under Ar. Workup: reaction mixture was diluted with water (30 mL) and 10% citric acid (10 mL), extracted with Et_2_O (2 × 20 mL), combined ethereal phases transferred to a volumetric flask, and diluted to the mark. An aliquot was taken, the solvent was removed in vacuo, and the residue dissolved with internal standard (2,4-dichlorotrifluorotoluene) solution in CDCl_3_.

b Reaction yield by ^19^F qNMR (offset −42 ppm).

c Gram scale (11 mmol), sequential one-pot reaction.

d Isolated yield–only extraction, no column chromatography, pure by NMR.

**Scheme 3:**
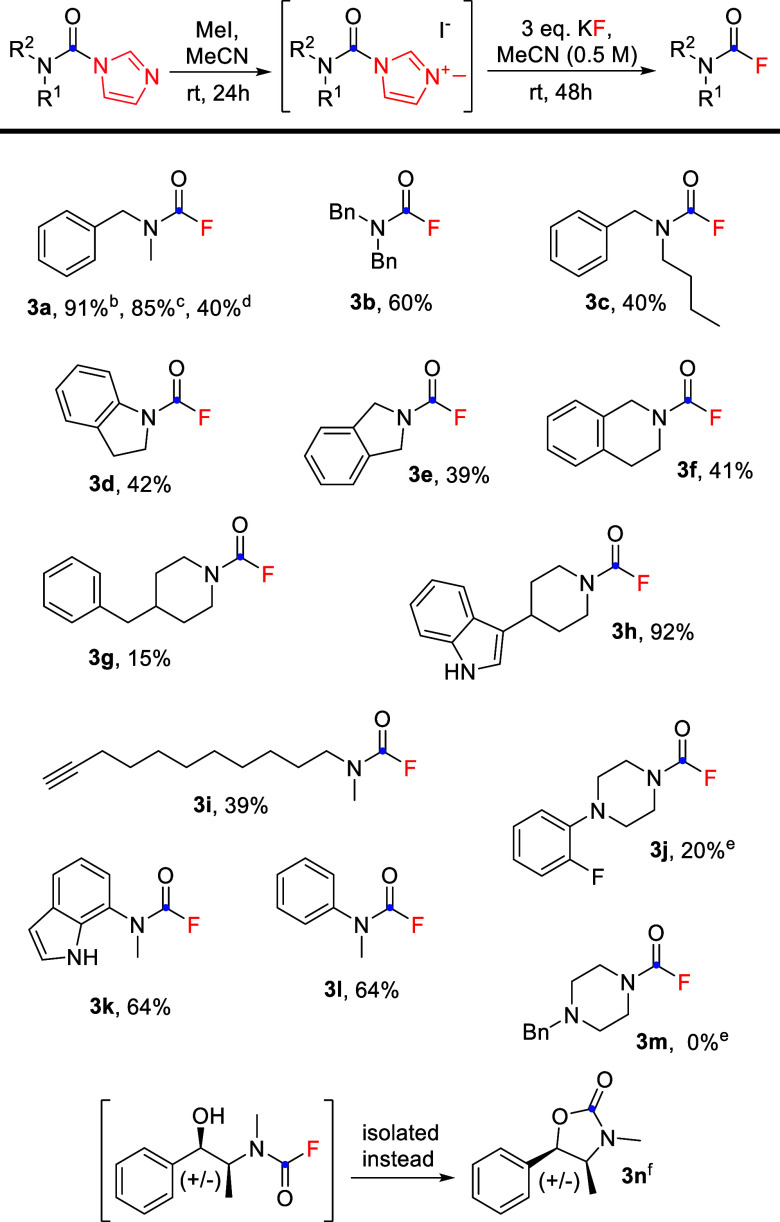
CF Synthesis−Reaction Scope 1–5 mmol scale following *GP2*. Isolated yields after column chromatography. Twelve mmol–gram scale. Eleven mmol–gram scale, purified by extraction only. One-pot reaction where both MeI and KF were added at once. **2j**- and **2m**-hydrochlorides instead of free bases were alkylated with MeI to avoid competing quaternization. Oxazolidinone was isolated instead of CF. Replacing potassium fluoride with less basic triethylamine trihydrofluoride led to a detectable carbamoyl fluoride signal in crude product ^19^F NMR spectrum–however, after column chromatography, only oxazolidinone was isolated.

Although formation of acyl chlorides from imidazolides and anhydrous HCl was already observed in 1966 by the “father” of carbonylazole chemistry, H. A. Staab,^30^ even the use of liquid HF surrogate, 70% hydrogen fluoride-pyridine, was ineffective in reaction with carbamoylimidazole **1a** (Table 1, entries 15–20). Therefore, preactivation of **1** by alkylation was necessary for an efficient transformation.^28^ Compared with the other reported synthetic routes to CFs, this procedure starts from secondary amines, uses only commercially available, cheap, stable, less toxic, and noncorrosive reagents, avoids exposure to acids, works on a gram scale, and does not require chromatographic purification (cf. Table 1, entry 21–purified by extraction only). It is, however, limited to *N*,*N*-disubstituted carbamoylimidazoles without other alkylation-prone functional groups or nearby nucleophilic groups that can cyclize to form a five- or six-membered ring system instead, as in case of **3n** (Scheme 3). *N*-Monosubstituted carbamoylimidazoles **4** were not good substrates, presumably due to the competing decomposition to isocyanates (Table 1, entries 22–27).^26^ Additionally, poorly basic *N*,*N*-diarylamines or sterically hindered amines (such as 2,6-dimethylpiperidine) failed to react with CDI to produce the required **1** intermediates.

This CDI-based activation followed by nucleophilic substitution with fluoride was also tested on other carbon- and phosphorus-based acids—however, fluorinated products were mostly formed in trace amounts only (Table 2). Nonetheless, an efficient conversion of *N*-sulfonyl- and *N*-(*O*-arylsulfonyl)imidazoles to sulfonyl fluorides^31,32^ and fluorosulfonates,^33,34^ respectively, was already reported.

**Table 2:**
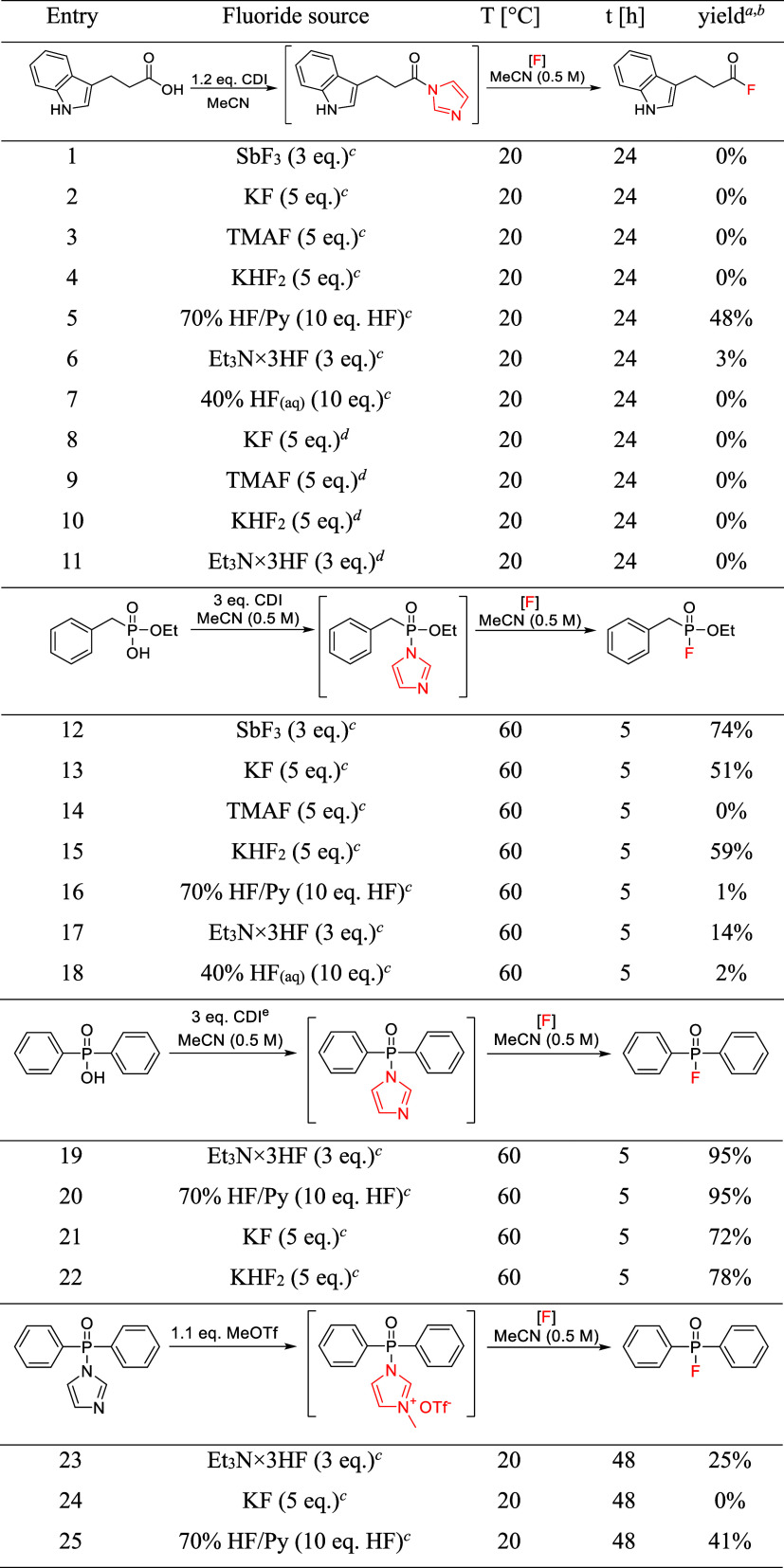

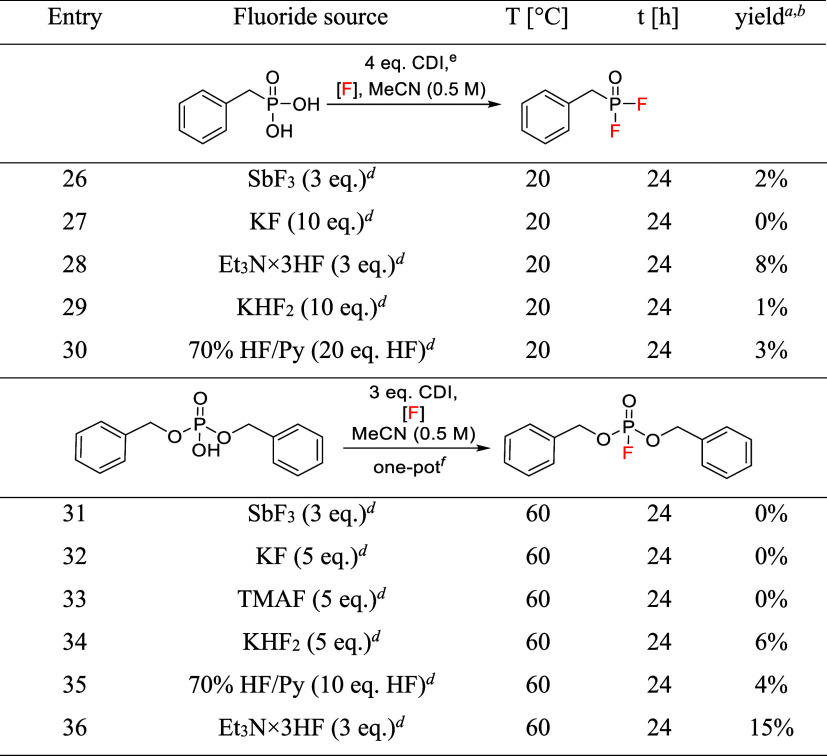
Activation with CDI and Subsequent Nucleophilic Substitution with fluoride–screening of Different C- and P-Acids^a^,^b^,^c^,^d^,^e^,^f^

a Conditions: 1 mmol scale, 2 mL MeCN (∼0.5 M), Ar. Workup: reaction mixture was diluted with water (30 mL) and 10% citric acid (10 mL), extracted with Et_2_O (2 × 20 mL), combined ethereal phases transferred to a 50 mL volumetric flask, and diluted to the mark. A 5 mL aliquot was taken, the solvent removed in vacuo, and the residue dissolved with 2,4-dichlorotrifluorotoluene internal standard (IS) solution in CDCl_3_.

b Reaction yield by^19^F qNMR (offset set to the arithmetic mean between product and IS signals, d1 = 15 s).

c Sequential one-pot reaction: activation for 1 h with CDI or methyl triflate, followed by addition of the fluoride.

d Concomitant one-pot reaction, all reagents were mixed at once.

e The excess of CDI is meant to suppress the formation of oligomeric phosphates.^35,36^

f The activation of dialkyl phosphates with CDI does not proceed as easily as with monoalkyl phosphates,^3^ while the literature data on phosphonic acids is lacking.

*O*-Aryl carbamates are well-known cholinesterase inhibitors–e.g., rivastigmine, which is registered for symptomatic treatment of Alzheimer’s disease.^37,38^ The increased reactivity of the electrophilic CF warhead and a smaller leaving group should make CFs efficient covalent cholinesterase inhibitors–therefore we have tested the synthesized compounds for inhibition of human acetyl- (hAChE) and butyrylcholinesterase (hBChE). As expected, these fragment-sized ligands were efficient cholinesterase inhibitors in the micro- and nanomolar range (Table 3). Mechanism of inhibition is presumed to be covalent—i.e., carbamoylation of catalytic Ser present in both enzymes. This was tentatively confirmed by time-dependent inhibition experiments, where inhibition increased with longer preincubation times (Figures S1–S2). As these compounds show a large potential as tool compounds and enzyme inhibitors for a larger enzyme class of serine hydrolases and beyond, we have also synthesized a CF-containing activity-based probe and performed a proteome-wide screening. However, these results fall outside the scope of this paper and will be reported in due course. Still, this highlights the yet-untapped potential of CFs in chemical biology and related fields.

**Table 3:** Cholinesterase Inhibition by CFs

Compound	hAChE	hBChE
	IC_50_ ± SEM [nM]^a^	
**3a**	5648 ± 947	156 ± 9
**3b**	2200 ± 270	69.6 ± 4.0
**3c**	4480 ± 1140	2430 ± 400
**3d**	4620 ± 1150	56.2 ± 3.7%^b^
**3e**	103,000 ± 33,120	32,200 ± 4800
**3f**	22,600 ± 5900	707 ± 122
**3g**	554 ± 79	2.78 ± 0.15
**3h**	18,700 ± 3300	23.5 ± 4.6
**3i**	5070 ± 1150	197 ± 36
**3j**	10,700 ± 1100	656 ± 32
**3k**	151 ± 8	5.56 ± 0.83

a IC_50_—half-maximal inhibitory concentration. SEM—standard error of the mean. Experiments were performed in triplicate, with preincubation time of 5 min.

b RA—residual activity [%] at 100 μM (mean ± SD of one independent experiment performed in triplicate). Compounds with RA > 50% were considered inactive.

## 3 Conclusion

In conclusion, we have developed a novel niche method for facile synthesis of tertiary carbamoyl fluorides starting from secondary amines. Carbamoyl group is introduced using a stable, solid phosgene surrogate CDI, is activated by alkylation of the imidazole leaving group, and last, CFs are obtained after exchange with an inorganic fluoride salt. Compared with other methods for synthesis of CFs, this three-step procedure is longer, but requires only commercially available, stable, noncorrosive reagents, while interstep manipulation involves only solvent removal/exchange and extraction without chromatographic purification. It is also suitable for acid-labile substrates and works on a gram scale. The product CFs are useful as synthetic intermediates as well as potential enzyme inhibitors (e.g., for serine hydrolases).

## 4 Experimental Section

### 4.1 Chemistry

#### 4.1.1 General Information

The reagents and solvents were used as received from commercial suppliers. Tetrahydrofuran (THF) was distilled from sodium/benzophenone and stored under Ar over 4 Å molecular sieves prior to use. After extraction organic phases were dried over anhydrous sodium sulfate. Reactions were monitored using analytical thin-layer chromatography (TLC) on silica gel 60 F_254_ Al plates. Developed plates were inspected under UV light and, if necessary, visualized with ninhydrin, vanillin/sulfuric acid, Dragendorff’s or potassium permanganate stains. Nuclear magnetic resonance spectra were recorded with a Bruker Avance III 400 MHz spectrometer at 400 MHz for ^1^H, 101 MHz for ^13^C, and 376 MHz for ^19^F nucleus, respectively, using CDCl_3_ with TMS as the internal standard, as solvent. Chemical shifts are reported in parts per million (ppm), ^1^H spectra are calibrated to the TMS peak (0 ppm) or the residual nonperdeuterated solvent peak, ^13^C spectra to the central peak of the deuterated solvent, while the ^19^F spectra were not calibrated. The multiplicities are reported as follows: s (singlet), d (doublet), t (triplet), q (quartet), m (multiplet), dd (doublet of doublets), ddd (doublet doublet of doublets), td (triplet of doublets), qd (quartet of doublets), and br (broad), number of equivalent nuclei (by integration), coupling constants (*J*) quoted in Hertz (Hz). Mass spectra were recorded on Agilent 6224 Accurate Mass TOF LC/MS and Thermo Scientific Q Exactive Plus LC–MS/MS spectrometers and IR spectra on Thermo Nicolet FT-IR spectrophotometer. Column chromatography was performed on silica gel (Silica gel 60, particle size: 0.035–0.070 mm, Merck). UPLC analyses were performed on Thermo Scientific Dionex UltiMate 3000 modular system (Thermo Fisher Scientific Inc.). The general method (method I) used a Waters Acquity UPLC HSS C18 SB column (2.1 × 50 mm, 1.8 μm) thermostated at 40 °C, with injection volume, 5 μL; sample, 0.1–0.2 mg/mL in MeOH; flow rate, 0.4 mL/min; detector λ, 220 and 254 nm; mobile phase A: 0.1% TFA (v/v) in water; mobile phase B: MeCN. Gradient: 0–2 min, 20% B; 2–5 min, 20%–90% B; 5–8 min, 90% B. Method II had the same conditions except gradient: 0–5 min, 20–100% B; 5–6 min, 20% B.^43^ The purities of the tested compounds were established to be ≥ 95%, as determined by UPLC, unless indicated otherwise.

##### 4.1.1.1 General Procedure 1 (GP1)-Preparation of *N*-Carbamoylimidazoles 1 (Literature Procedure^28^)

Secondary amine (1.0 mmol) was dissolved in DCM (5 mL), CDI (1.1 eq., 178 mg) was added, and the reaction mixture stirred at rt for 12 h. The organic phase was extracted with water (2 × 10 mL), dried with sodium sulfate, and solvent removed in vacuo to afford crude **1**, which was sufficiently pure to be directly used further.

##### 4.1.1.2 General Procedure 2 (GP2)-Synthesis of Carbamoyl Fluorides

**1** from *GP1* (1.0 mmol) and methyl iodide (1.5 eq., 93 μL) were stirred in MeCN (2 mL) at rt under Ar for 24 h. The solvent was removed in vacuo, the residue dissolved in MeCN (2 mL), potassium fluoride (5 eq., 290 mg) was added, and the reaction mixture vigorously stirred at rt under Ar for 24 h. The solvent was removed in vacuo, 10% citric acid (10 mL) and DCM (10 mL) were added, organic phase separated, dried with sodium sulfate, and solvent removed in vacuo to afford crude carbamoyl fluoride **3**, which was optionally purified by column chromatography on silica.

#### 4.1.2 1-(Benzyl(methyl)carbamoyl)-3-methyl-1*H*-imidazol-3-ium Iodide (**2a**)

For the screening of reaction conditions, **2a** was prepared in advance. *N*-Benzyl-*N*-methyl-1*H*-imidazole-1-carboxamide **1a** (3076 mg, 14.29 mmol) and methyl iodide (1.5 eq., 1.33 mL) in MeCN (15 mL) were stirred at rt under Ar for 24 h. The reaction mixture was concentrated to half its initial volume, diluted with Et_2_O (50 mL), sonicated for 5 min, the precipitate filtered, washed with Et_2_O (2 × 15 mL), and dried in vacuo to afford 1-(benzyl(methyl)carbamoyl)-3-methyl-1*H*-imidazol-3-ium iodide as beige solid (yield: 4660 mg, 13.05 mmol, 93%), which was kept under Ar and directly used further.

#### 4.1.3 Benzyl(methyl)carbamic Fluoride (**3a**)

Prepared following *GP2* on a scale of 12.0 mmol and isolated by column chromatography on silica (PE/EtOAc = 20:1). Yield: 1817 mg (10.87 mmol, 91%) of beige oil.^1^ ESI-HRMS: *m*/*z* = 168.0823 (MH^+^); C_9_H_11_NOF requires: *m*/*z* = 168.0819 (MH^+^). ν_max_ 3065, 3032, 2935, 1776, 1455, 1402, 1221, 1112, 999, 748, 697 cm^–1^. Purity: UPLC (method II, 220 nm): t_r_ = 2.407 min, 99.1% total area. ^1^H NMR (400 MHz, CDCl_3_) δ = 2.88 and 2.93 (s, 3H), 4.43 and 4.47 (s, 2H), 7.22–7.26 (m, 1H), 7.27–7.41 (m, 4H). ^13^C NMR (101 MHz, CDCl_3_) δ = 33.9 (d, *J* = 3.8), 35.0 (d, *J* = 1.5), 53.0 (d, *J* = 3.6), 53.5 (d, *J* = 1.0), 127.5, 128.1, 128.2, 128.9, 129.0, 135.4, 147.6 (d, *J* = 286.1), 148.2 (d, *J* = 286.9). ^19^F NMR (376 MHz, CDCl_3_) δ = −23.4, −21.8.

Table 1, entry 21: prepared following *GP2* on a scale of 11.37 mmol, no column chromatography–extraction only. Yield: 85%. ^1^H NMR (400 MHz, CDCl_3_) δ = 2.88 and 2.93 (2 × s, 3H), 4.43 and 4.47 (2 × s, 2H), 7.22–7.26 (m, 1H), 7.27–7.41 (m, 4H). ^19^F NMR (376 MHz, CDCl_3_) δ = −23.4, −21.8.

Scheme 3, footnote d: one-pot concomitant reaction on a scale of 1 mmol. Isolated by column chromatography on silica (PE/EtOAc = 20:1). Yield: 67 mg (0.40 mmol, 40%) of beige oil.

#### 4.1.4 Dibenzylcarbamic Fluoride (**3b**)

Prepared according to *GP2* on a scale of 1.17 mmol and isolated by column chromatography on silica (PE/EtOAc = 20:1). Yield: 171 mg (0.703 mmol, 60%) of beige oil.^1^ ESI-HRMS: *m*/*z* = 244.1131 (MH^+^); C_15_H_15_FNO requires: *m*/*z* = 244.1132 (MH^+^). ν_max_ 3065, 3032, 2937, 1778, 1496, 1454, 1419, 1220, 1082, 1033, 747, 696 cm^–1^. Purity: ^19^F qNMR −100%. ^1^H NMR (400 MHz, CDCl_3_) δ = 4.32 (s, 2H), 4.40 (s, 2H), 7.17–7.22 (m, 2H), 7.24–7.28 (m, 2H), 7.29–7.39 (m, 6H). ^13^C NMR (101 MHz, CDCl_3_) δ = 49.7, 49.7, 50.5, 127.8, 128.2, 128.3, 128.5, 129.0, 129.0, 135.4, 135.4, 148.2 (d, *J* = 288.0). ^19^F NMR (376 MHz, CDCl_3_) δ = −22.6.

#### 4.1.5 Benzyl(butyl)carbamic Fluoride (**3c**)

Prepared according to *GP2* on a scale of 1.06 mmol and isolated by column chromatography on silica (PE/EtOAc = 5:1). Yield: 89 mg (0.425 mmol, 40%) of beige oil. ESI-HRMS: *m*/*z* = 210.1288 (MH^+^); C_12_H_17_FNO requires: *m*/*z* = 210.1289 (MH^+^). ν_max_ 2959, 2934, 2874, 1777, 1455, 1420, 1218, 1129, 1032, 750, 728, 697 cm^–1^. Purity: ^19^F qNMR −99%, UPLC (method II, 220 nm): t_r_ = 3.453 min, 97.9% total area. ^1^H NMR (400 MHz, CDCl_3_) δ = 0.91 (td, *J* = 5.1, 7.3, 3H), 1.23–1.36 (m, 2H), 1.47–1.61 (m, 2H), 3.16 (t, *J* = 7.4, 1H), 3.24 (t, *J* = 7.6, 1H), 4.43 and 4.48 (2 × s, 2H), 7.22–7.26 (m, 1H), 7.28–7.40 (m, 4H). ^13^C NMR (101 MHz, CDCl_3_) δ = 13.7, 13.8, 19.8, 19.9, 29.1, 30.2, 46.7, 46.8, 47.8, 47.8, 50.9, 51.0, 51.5, 127.5, 128.1, 128.1, 128.9, 129.0, 136.0, 147.6 (d, *J* = 288.1), 148.6 (d, *J* = 285.7). ^19^F NMR (376 MHz, CDCl_3_) δ = −22.8, −21.6.

#### 4.1.6 Indoline-1-carbonyl Fluoride (**3d**)

Prepared according to *GP2* on a scale of 5.08 mmol and isolated by column chromatography on silica (PE/EtOAc = 10:1). Yield: 566 mg (3.43 mmol, 68%) of white solid.^1^ ESI-HRMS: *m*/*z* = 166.0663 (MH^+^); C_9_H_9_FNO requires: *m*/*z* = 166.0663 (MH^+^). ν_max_ 3052, 2982, 2945, 2927, 2867, 1781, 1598, 1486, 1462, 1444, 1402, 1337, 1316, 1291, 1258, 1105, 941, 751, 734 cm^–1^. Purity: UPLC (method II, 220 nm): t_r_ = 2.683 min, 99.4% total area. ^1^H NMR (400 MHz, CDCl_3_) δ = 3.15–3.22 (m, 2H), 4.02–4.11 (m, 2H), 7.03–7.10 (m, 1H), 7.18–7.32 and 7.73–7.78 (m, 3H). ^13^C NMR (101 MHz, CDCl_3_) δ = 27.4, 27.5, 47.8, 48.3, 115.0, 115.1, 115.2, 124.4, 124.6, 125.1, 125.6, 127.9, 128.0, 128.0, 131.3, 131.3, 131.5, 139.7, 139.7, 140.4, 143.2 (d, *J* = 289.3), 144.2 (d, *J* = 295.8). ^19^F NMR (376 MHz, CDCl_3_) δ = −13.1, −5.0.

#### 4.1.7 Isoindoline-2-carbonyl Fluoride (**3e**)

Prepared according to *GP2* on a scale of 4.38 mmol and isolated by column chromatography on silica (PE/EtOAc = 10:1). Yield: 467 mg (2.83 mmol, 65%) of white solid.^22^ ESI-HRMS: *m*/*z* = 166.0662 (MH^+^); C_9_H_9_FNO requires: *m*/*z* = 166.0663 (MH^+^). ν_max_ 3201, 3081, 3024, 2944, 2884, 1785, 1466, 1400, 1283, 1175, 1010, 754, 743 cm^–1^. Purity: UPLC (method II, 220 nm): t_r_ = 2.420 min, 99.4% total area. ^1^H NMR (400 MHz, CDCl_3_) δ = 4.79 (br s, 2H), 4.81 (br s, 2H), 7.23–7.36 (m, 4H). ^13^C NMR (101 MHz, CDCl_3_) δ = 52.6, 53.1, 122.8, 122.9, 122.9, 128.1, 128.2, 135.4, 135.5, 144.6, 147.5 ^19^F NMR (376 MHz, CDCl_3_) δ = −14.3.

#### 4.1.8 3,4-Dihydroisoquinoline-2(1*H*)-carbonyl Fluoride (**3f**)

Prepared according to *GP2* on a scale of 2.35 mmol and isolated by column chromatography on silica (PE/EtOAc = 20:1). Yield: 517 mg (2.89 mol, 51%) of beige oil.^39^ ESI-HRMS: *m*/*z* = 180.0816 (MH^+^); C_10_H_11_FNO requires: *m*/*z* = 180.0819 (MH^+^). ν_max_ 3026, 2900, 1775, 1428, 1407, 1285, 1220, 1112, 1060, 1023, 952, 926, 831, 741, 653 cm^–1^. Purity: UPLC (method II, 220 nm): t_r_ = 2.983 min, 99.1% total area. ^1^H NMR (400 MHz, CDCl_3_) δ = 2.93 (q, *J* = 6.4, 2H), 3.71 (dt, *J* = 6.0, 10.8, 2H), 4.64 (d, *J* = 2.7, 2H), 7.09–7.26 (m, 4H). ^13^C NMR (101 MHz, CDCl_3_) δ = 28.2, 28.7, 42.5, 42.5, 42.7, 46.1, 46.1, 46.6, 126.1, 126.3, 126.7, 126.8, 127.1, 127.1, 128.7, 128.9, 131.5, 131.8, 133.6, 133.8, 146.6 (d, J = 284.7), 146.7 (d, J = 286.0). ^19^F NMR (376 MHz, CDCl_3_) δ = −23.2, −20.2. Mixture of rotamers and conformers.

#### 4.1.9 4-Benzylpiperidine-1-carbonyl Fluoride (**3g**)

Prepared according to *GP2* on a scale of 1.30 mmol and isolated by column chromatography on silica (PE/EtOAc = 20:1). Yield: 44 mg (0.199 mmol, 15%) of beige solid. ESI-HRMS: *m*/*z* = 222.1285 (MH^+^); C_13_H_17_FNO requires: *m*/*z* = 222.1289 (MH^+^). ν_max_ 2943, 2912, 2847, 1777, 1430, 1265, 1238, 751, 742, 701 cm^–1^. Purity: UPLC (method II, 220 nm): t_r_ = 3.390 min, 100% total area. ^1^H NMR (400 MHz, CDCl_3_) δ = 1.18–1.33 (m, 2H), 1.67–1.79 (m, 3H), 2.57 (d, *J* = 6.8, 2H), 2.79–2.91 (m, 2H), 3.90–3.96 (m, 1H), 4.03 and 4.06 (2 × dq, *J* = 2.5, 4.8, 1H), 7.12–7.16 (m, 2H), 7.19–7.24 (m, 1H), 7.27–7.33 (m, 2H). ^13^C NMR (101 MHz, CDCl_3_) δ = 31.4, 31.8, 37.7, 42.9, 45.1, 45.2, 45.5, 45.5, 126.3, 128.5, 129.2, 139.7, 146.6 (d, *J* = 287.0). ^19^F NMR (376 MHz, CDCl_3_) δ = −24.7.

#### 4.1.10 4-(1*H*-Indol-3-yl)piperidine-1-carbonyl Fluoride (**3h**)

Prepared according to *GP2* on a scale of 0.42 mmol and isolated by column chromatography on silica (PE/EtOAc = 4:1). Yield: 91 mg (0.37 mol, 92%) of beige solid. ESI-HRMS: *m*/*z* = 247.1241 (MH^+^); C_14_H_16_FN_2_O requires: *m*/*z* = 247.1241 (MH^+^). ν_max_ 3356, 2949, 2877, 1752, 1459, 1433, 1219, 1105, 1001, 733 cm^–1^. Purity: UPLC (method II, 220 nm): t_r_ = 2.983 min, 99.9% total area. ^1^H NMR (400 MHz, CDCl_3_) δ = 1.69–1.86 (m, 2H), 2.10–2.19 (m, 2H), 3.02–3.19 (m, 3H), 4.06–4.13 (m, 1H), 4.19 and 4.23 (2 × dq, *J* = 2.7, 4.9, 1H), 6.97–6.98 (m, 1H), 7.14 (ddd, *J* = 1.0, 7.1, 8.0, 1H), 7.22 (ddd, *J* = 1.1, 7.1, 8.1, 1H),, 7.39 (dt, *J* = 0.8, 8.2, 1H), 7.60–7.64 (m, 1H), 8.06 (s, 1H). ^13^C NMR (101 MHz, CDCl_3_) δ = 32.2, 32.8, 33.3, 45.6, 45.7, 46.0, 111.5, 118.9, 119.5, 119.9, 120.0, 122.4, 126.4, 136.5, 145.3, 148.2. ^19^F NMR (376 MHz, CDCl_3_) δ = −24.6.

#### 4.1.11 Methyl(undec-10-yn-1-yl)carbamic Fluoride (**3i**)

Prepared according to *GP2* on a scale of 1.59 mmol and isolated by column chromatography on silica (1. PE/DCM = 3:1; 2. DCM). Yield: 139 mg (0.611 mmol, 39%) of beige oil. ESI-HRMS: *m*/*z* = 228.1756 (MH^+^); C_13_H_23_NOF requires: *m*/*z* = 228.1758 (MH^+^). ν_max_ 3309, 2929, 2856, 1782, 1465, 1404, 1155, 1120, 1089, 997, 751, 627 cm^–1^. ^1^H NMR (400 MHz, CDCl_3_) δ = 1.23–1.44 (m, 10H), 1.47–1.61 (m, 4H), 1.91–1.95 (m, 1H), 2.17 (t, *J* = 6.0, 2H), 2.94 (d, *J* = 8.7, 3H), 3.19–3.29 (m, 2H). ^13^C NMR (101 MHz, CDCl_3_) δ = 18.5, 26.5, 26.5, 27.0, 27.9, 28.5, 28.5, 28.8, 28.8, 29.1, 29.3, 29.3, 29.4, 34.4, 34.4, 35.4, 35.4, 49.6, 49.6, 50.2, 50.2, 68.2, 68.2, 84.8, 84.8, 146.4 (d, *J* = 286.8), 148.4 (d, *J* = 286.4). ^19^F NMR (376 MHz, CDCl_3_) δ = −23.9, −20.9.

#### 4.1.12 4-(2-Fluorophenyl)piperazine-1-carbonyl Fluoride (**3j**)

Prepared according to *GP2* on a scale of 1.30 mmol and isolated by column chromatography on silica (PE/EtOAc = 10:1). Yield: 58 mg (0.256 mmol, 20%) of white solid. ESI-HRMS: *m*/*z* = 227.0987 (MH^+^); C_11_H_13_F_2_N_2_O requires: *m*/*z* = 227.0991 (MH^+^). ν_max_ 2922, 2830, 1779, 1500, 1430, 1231, 1214, 1032, 913, 744 cm^–1^. Purity: UPLC (method II, 220 nm): t_r_ = 2.830 min, 99.9% total area. ^1^H NMR (400 MHz, CDCl_3_) δ = 3.06–3.14 (m, 4H), 3.62–3.69 (m, 4H), 6.91–7.11 (m, 4H). ^13^C NMR (101 MHz, CDCl_3_) δ = 45.0, 45.0, 50.0, 50.1, 50.4, 50.4, 116.4 (d, *J* = 20.6), 119.4 (d, *J* = 2.6), 123.6 (d, *J* = 8.0), 124.7 (d, *J* = 3.7), 139.4 (d, *J* = 8.7), 146.4 (d, *J* = 286.3), 155.8 (d, *J* = 246.1). ^19^F NMR (376 MHz, CDCl_3_) δ = −123.1 (ddd, *J* = 4.8, 8.6, 12.0), −24.3.

#### 4.1.13 (1*H*-Indol-7-yl)(methyl)carbamic Fluoride (**3k**)

7-Nitro-1*H*-indole (5000 mg, 30.84 mmol) and 10% Pd/C (100 mg) in MeOH (100 mL) were cooled in an ice bath, and sodium borohydride was added in 50 mg portions, waiting in between for the foaming to subside, until the yellow color disappeared and the conversion was complete (by TLC). The suspension was filtered through Celite, the solvent removed in vacuo, residue diluted with water (100 mL), 50% sodium hydroxide_(aq)_ (10 mL) was added, and 7-aminoindole^40^ extracted with Et_2_O (2 × 30 mL). The organic phase was dried with sodium sulfate, the solvent removed in vacuo, the residue dissolved in ethyl formate (50 mL), transferred to a pressure tube, and stirred at 100 °C for 18 h. The solvent was removed in vacuo, the residue evaporated with toluene (50 mL), crude *N*-(1*H*-indol-7-yl)formamide dissolved in THF (50 mL), and lithium aluminum hydride solution (2.4 M in THF, 3.0 eq., 38.6 mL) was slowly added under Ar while stirring in an icebath. The reaction mixture was stirred at 50 °C under Ar for 12 h and then cautiously quenched with brine while being cooled in an icebath. The resulting gray solids were suspended in Et_2_O (50 mL), sonicated in an ultrasonic cleaning bath for 5 min, filtered, and washed with Et_2_O (2 × 20 mL). The ethereal extracts were dried over sodium sulfate, filtered, and volatile components evaporated in vacuo to afford crude *N*-methyl-1*H*-indol-7-amine. This brown-greenish solid (3450 mg, 23.6 mmol) was dissolved in THF (30 mL), CDI (1.5 eq., 5740 mg) was added, and the mixture heated at 60 °C for 18 h. Water (20 mL) was then added, THF removed in vacuo, and the resulting suspension filtered, washed with water (2 × 20 mL) and Et_2_O (20 mL), and air-dried to afford crude *N*-(1*H*-indol-7-yl)-*N*-methyl-1*H*-imidazole-1-carboxamide **2k** as greenish solid (4500 mg, 18.73 mmol, 61% yield over 4 steps). The crude product was purified by column chromatography on silica (1. PE/EtOAc = 1:1; 2. EtOAc). mp 196.0–197.7 °C. ESI-HRMS: *m*/*z* = 241.1081 (MH^+^); C_13_H_13_N_4_O requires: *m*/*z* = 241.1084 (MH^+^). ν_max_ 3123, 2918, 2873, 1699, 1585, 1422, 1382, 1298, 1201, 1122, 801, 733 cm^–1^. Purity: UPLC (method I, 254 nm): t_r_ = 1.293 min, 97.3% total area. ^1^H NMR (400 MHz, acetone-*d*_6_) δ = 3.48 (s, 3H), 6.60 (d, *J* = 3.2, 1H), 6.62–6.64 (m, 1H), 6.78 (s, 1H), 7.03 (s, 1H), 7.04 (d, *J* = 1.2, 1H), 7.41 (s, 1H), 7.43 (d, *J* = 3.2, 1H), 7.61–7.65 (m, 1H), 10.83 (s, 1H). ^13^C NMR (101 MHz, acetone-*d*_6_) δ = 39.4, 103.8, 119.0, 120.5, 120.8, 121.8, 127.1, 128.2, 129.3, 131.5, 132.8, 137.9, 151.1.

**3k** was prepared according to *GP2* from **2k** (240 mg, 1 mmol) and isolated by column chromatography on silica (PE/EtOAc = 5:1). Yield: 123 mg (0.64 mmol, 64%) of beige semisolid. ESI-HRMS: *m*/*z* = 193.0777 (MH^+^); C_10_H_10_FN_2_O requires: *m*/*z* = 193.0772 (MH^+^). ν_max_ 3361, 3310, 3113, 1792, 1589, 1497, 1421, 1390, 1339, 1294, 1279, 1187, 1138, 1068, 1012, 915, 801, 732, 641, 565, 515 cm^–1^. Purity: UPLC (method II, 220 nm): t_r_ = 2.447 min, 97.3% total area. ^1^H NMR (400 MHz, CDCl_3_) δ = 3.40 (s) and 3.42 (d, *J* = 1.0, 3H), 6.56 and 6.58 (2 × dd, *J* = 2.0, 3.1, 1H), 7.03–7.12 (m, 2H), 7.16 and 7.21 (2 × t, *J* = 2.8, 1H), 7.59–7.65 (m, 1H), 8.89 and 9.42 (2 × s, 1H). ^13^C NMR (101 MHz, CDCl_3_) δ = 38.8 (d, *J* = 2.7), 38.9 (d, *J* = 2.0), 103.2, 103.3, 118.1, 118.1, 119.2, 119.9, 120.0, 120.9, 121.3, 124.5, 125.5, 125.6, 125.7, 130.3, 130.5, 130.6, 131.6, 147.1 (d, *J* = 292.3), 147.8 (d, *J* = 286.7). ^19^F NMR (376 MHz, CDCl_3_) δ = −16.9, −15.9. Two sets of conformer signals.

#### 4.1.14 Methyl(phenyl)carbamic Fluoride (**3l**)

Prepared according to *GP2* on a scale of 3.18 mmol and isolated by column chromatography on silica (PE/EtOAc = 10:1). Yield: 311 mg (2.03 mmol, 64%) of beige oil.^1^ ESI-HRMS: *m*/*z* = 154.0661 (MH^+^); C_8_H_9_ONF requires: *m*/*z* = 154.0663 (MH^+^). ν_max_ 3068, 2950, 1779, 1598, 1498, 1369, 1114, 943, 767, 748, 694, 585 cm^–1^. Purity: ^19^F qNMR −99%, UPLC (method II, 220 nm): t_r_ = 2.193 min, 99.9% total area. ^1^H NMR (400 MHz, CDCl_3_) δ = 3.25 and 3.27 (s, 3H), 7.11–7.35 (m, 5H). ^13^C NMR (101 MHz, CDCl_3_) δ = 37.9, 38.9, 124.7, 125.6, 127.2, 127.8, 129.3, 129.5, 140.8, 141.7, 146.4 (d, *J* = 285.5), 146.7 (d, *J* = 288.9). ^19^F NMR (376 MHz, CDCl_3_) δ = −16.1, −16.0.

#### 4.1.15 (4*S*,5*R*)- and (4*R*,5*S*)-3,4-dimethyl-5-phenyloxazolidin-2-one (**3n**)

Prepared according to *GP2* on a scale of 1.03 mmol and isolated by column chromatography on silica (PE/EtOAc = 3:1). Isolated yield: 154 mg (0.805 mmol, 78%) of white solid.^41^ ESI-HRMS: *m*/*z* = 192.1017 (MH^+^); C_11_H_14_O_2_N requires: *m*/*z* = 192.1019 (MH^+^). ν_max_ 3499, 3032, 2976, 2931, 1743, 1426, 1393, 1242, 1148, 1106, 1019, 999, 762, 700, 664 cm^–1^. ^1^H NMR (400 MHz, CDCl_3_) δ = 0.76 (d, *J* = 6.6, 3H), 2.86 (s, 3H), 4.03 (dq, *J* = 6.6, 8.2, 1H), 5.58 (d, *J* = 8.2, 1H), 7.25–7.28 (m, 2H), 7.30–7.39 (m, 3H). ^13^C NMR (101 MHz, CDCl_3_) δ = 14.1, 28.8, 56.9, 78.2, 126.0, 128.3, 128.3, 135.1, 157.9. ^19^F NMR (376 MHz, CDCl_3_)-no signal.

### 4.2 Inhibition of ChEs

The inhibitory potencies of the compounds against the ChEs were determined using the method of Ellman following the procedure described previously.^42−44^ Briefly, compound stock solutions in DMSO were incubated with Ellman’s reagent and the ChEs (final concentrations: 370 μM Ellman’s reagent, approximately 1 nM or 50 pM hBChE or hAChE, respectively) in 0.1 M sodium phosphate pH 8.0 for 5 min at 20 °C. For time-dependency measurements, the preincubation time was varied (1, 5, 15, 30 or 60 min). The reactions were started by the addition of the substrate (final concentration, 500 μM butyrylthiocholine iodide (BTChI) or acetylthiocholine iodide for hBChE and hAChE, respectively). The final content of DMSO was always 1%. The increase in absorbance at 412 nm was monitored for 2 min using a microplate reader (Synergy HT, BioTek Instruments, VT, USA). The initial velocities in the presence (*v*_i_) and absence (*v*_o_) of the test compounds were calculated. The inhibitory potencies were expressed as the residual activities, according to RA = (*v*_i_ – *b*)/(*v*_o_ – *b*), where *b* is the blank value using phosphate buffer without ChEs. For IC_50_ determinations, at least seven different concentrations for each compound were used. The IC_50_ values were obtained by plotting the residual ChE activities against the applied inhibitor log_10_ concentrations, with the experimental data fitted to a four-parameter logistic function (GraphPad Prism 9.4). Rivastigmine and donepezil were used as positive controls.

## Supporting Information Available

Cholinesterase inhibition time-dependency data (Figures S1–S2), NMR spectra (Figures S3–S53) (PDF)

## References

[ref1] Bonnefoy C.; Chefdeville E.; Tourvieille C.; Panossian A.; Hanquet G.; Leroux F.; Toulgoat F.; Billard T. Study of Carbamoyl Fluoride: Synthesis, Properties and Applications. Chem. - Eur. J. 2022, 28 (43), e202201589 10.1002/chem.202201589. 35639343

[ref2] Myers D. K.; Kemp A. Inhibition of Esterases by the Fluorides of Organic Acids. Nature 1954, 173 (4392), 33–34. 10.1038/173033a0. 13119739

[ref3] Metzger H. P.; Wilson I. B. Evidence for an Electrophilic Mechanism in Catalysis by Hydrolytic Enzymes. Biochemistry 1964, 3 (7), 926–931. 10.1021/bi00895a013. 14214081

[ref4] Johnson M. K. Sensitivity and Selectivity of Compounds Interacting with Neuropathy Target Esterase: Further Structure-Activity Studies. Biochem. Pharmacol. 1988, 37 (21), 4095–4104. 10.1016/0006-2952(88)90101-3. 3190748

[ref5] Venkatasubban K. S.; Johnson J. L.; Thomas J. L.; Fauq A.; Cusack B.; Rosenberry T. L. Decarbamoylation of Acetylcholinesterases Is Markedly Slowed as Carbamoyl Groups Increase in Size. Arch. Biochem. Biophys. 2018, 655, 67–74. 10.1016/j.abb.2018.08.006. 30098983 PMC6204216

[ref6] Cho H.; Jang S.; Lee K.; Cha D.; Min S.-J. Visible-Light-Induced DDQ-Catalyzed Fluorocarbamoylation Using CF3SO2Na and Oxygen. Org. Lett. 2023, 25 (48), 8558–8563. 10.1021/acs.orglett.3c03335. 37987781

[ref7] Zivkovic F. G.; Nielsen C.D.-T.; Schoenebeck F. Access to N–CF3 Formamides by Reduction of N–CF3 Carbamoyl Fluorides. Angew. Chem., Int. Ed. 2022, 61 (52), e202213829 10.1002/anie.202213829. PMC10099374 36308723

[ref8] Scattolin T.; Bouayad-Gervais S.; Schoenebeck F. Straightforward Access to N-Trifluoromethyl Amides, Carbamates, Thiocarbamates and Ureas. Nature 2019, 573 (7772), 102–107. 10.1038/s41586-019-1518-3. 31485055

[ref9] Jabbarpoor M.; LeBlanc J.; Chen Z.; Cadwallader D.; Le C. M. Pd-Catalyzed Suzuki-Type Cross-Coupling of 2-Pyridyl Carbamoyl Fluorides. Chem. Commun. 2024, 60, 8700 10.1039/D4CC02431A. 39054900

[ref10] McKnight E. A.; Arora R.; Pradhan E.; Fujisato Y. H.; Ajayi A. J.; Lautens M.; Zeng T.; Le C. M. BF3-Catalyzed Intramolecular Fluorocarbamoylation of Alkynes via Halide Recycling. J. Am. Chem. Soc. 2023, 145 (20), 11012–11018. 10.1021/jacs.3c03982. 37172320

[ref11] Cadwallader D.; Shevchuk D.; Tiburcio T. R.; Le C. M. Fluoride-Catalyzed Cross-Coupling of Carbamoyl Fluorides and Alkynylsilanes. Org. Lett. 2023, 25 (40), 7369–7373. 10.1021/acs.orglett.3c02871. 37767985

[ref12] He F.; Hou L.; Wu X.; Ding H.; Qu J.; Chen Y. Enantioselective Synthesis of α-Alkenylated γ-Lactam Enabled by Ni-Catalyzed 1,4-Arylcarbamoylation of 1,3-Dienes. CCS Chem. 2023, 5 (2), 341–349. 10.31635/ccschem.022.202202010.

[ref13] Nielsen C. D.-T.; Zivkovic F. G.; Schoenebeck F. Synthesis of N-CF3 Alkynamides and Derivatives Enabled by Ni-Catalyzed Alkynylation of N-CF3 Carbamoyl Fluorides. J. Am. Chem. Soc. 2021, 143 (33), 13029–13033. 10.1021/jacs.1c07780. 34428910

[ref14] Li Y.; Zhang F.-P.; Wang R.-H.; Qi S.-L.; Luan Y.-X.; Ye M. Carbamoyl Fluoride-Enabled Enantioselective Ni-Catalyzed Carbocarbamoylation of Unactivated Alkenes. J. Am. Chem. Soc. 2020, 142 (47), 19844–19849. 10.1021/jacs.0c09949. 33170685

[ref15] Yang Y.; Taponard A.; Vantourout J. C.; Tlili A. Synthesis of Fluorinated Amines: A Personal Account. ACS Org. Inorg. Au 2023, 3 (6), 364–370. 10.1021/acsorginorgau.3c00029. 38075451 PMC10704584

[ref16] Liu L.; Gu Y.-C.; Zhang C.-P. Recent Advances in the Synthesis and Transformation of Carbamoyl Fluorides, Fluoroformates, and Their Analogues. Chem. Rec. 2023, 23 (9), e202300071 10.1002/tcr.202300071. 37098875

[ref17] Turksoy A.; Scattolin T.; Bouayad-Gervais S.; Schoenebeck F. Facile Access to AgOCF3 and Its New Applications as a Reservoir for OCF2 for the Direct Synthesis of N–CF3, Aryl or Alkyl Carbamoyl Fluorides. Chem. - Eur. J. 2020, 26 (10), 2183–2186. 10.1002/chem.202000116. 31922296

[ref18] Cuomo J.; Olofson R. A. Efficient and Convenient Synthesis of Fluoroformates and Carbamoyl Fluorides. J. Org. Chem. 1979, 44 (6), 1016–1017. 10.1021/jo01320a034.

[ref19] Pulikkottil F.; Burnett J. S.; Saiter J.; Goodall C. A. I.; Claringbold B.; Lam K. eFluorination for the Rapid Synthesis of Carbamoyl Fluorides from Oxamic Acids. Org. Lett. 2024, 26 (29), 6103–6108. 10.1021/acs.orglett.4c01605. 39016380 PMC11287745

[ref20] Cadwallader D.; Tiburcio T. R.; Cieszynski G. A.; Le C. M. Synthesis of Carbamoyl Fluorides Using a Difluorophosgene Surrogate Derived from Difluorocarbene and Pyridine N-Oxides. J. Org. Chem. 2022, 87 (17), 11457–11468. 10.1021/acs.joc.2c01017. 35972076

[ref21] Baars H.; Engel J.; Mertens L.; Meister D.; Bolm C. The Reactivity of Difluorocarbene with Hydroxylamines: Synthesis of Carbamoyl Fluorides. Adv. Synth. Catal. 2016, 358 (14), 2293–2299. 10.1002/adsc.201600308.

[ref22] Song J. W.; Lim H. N. Synthesis of Carbamoyl Fluorides via a Selective Fluorinative Beckmann Fragmentation. Org. Lett. 2021, 23 (14), 5394–5399. 10.1021/acs.orglett.1c01721. 34197129

[ref23] Taponard A.; Jarrosson T.; Khrouz L.; Médebielle M.; Broggi J.; Tlili A. Metal-Free SF6 Activation: A New SF5-Based Reagent Enables Deoxyfluorination and Pentafluorosulfanylation Reactions. Angew. Chem., Int. Ed. 2022, 61 (27), e202204623 10.1002/anie.202204623. 35471641

[ref24] Zhen L.; Fan H.; Wang X.; Jiang L. Synthesis of Thiocarbamoyl Fluorides and Isothiocyanates Using CF3SiMe3 and Elemental Sulfur or AgSCF3 and KBr with Amines. Org. Lett. 2019, 21 (7), 2106–2110. 10.1021/acs.orglett.9b00383. 30855147

[ref25] Liu L.; Ran L.-Y.; Gu Y.; Zhang C.-P. Facile Synthesis of Selenocarbamyl Fluorides, Selenoureas and Their Derivatives with [Me4N][SeCF3]. Org. Chem. Front. 2021, 8 (20), 5736–5743. 10.1039/D1QO00736J.

[ref26] Buckley G. D.; Piggott H. A.; Welch A. J. E. 227 Preparation of Carbamyl Fluorides by the Action of Anhydrous Hydrogen Fluoride on Isocyanates. J. Chem. Soc. 1945, (0), 864–865. 10.1039/jr9450000864.

[ref27] Olah G. A.; Welch J. T.; Vankar Y. D.; Nojima M.; Kerekes I.; Olah J. A. Synthetic Methods and Reactions. 63. Pyridinium Poly(Hydrogen Fluoride) (30% Pyridine-70% Hydrogen Fluoride): A Convenient Reagent for Organic Fluorination Reactions. J. Org. Chem. 1979, 44 (22), 3872–3881. 10.1021/jo01336a027.

[ref28] Grzyb J. A.; Shen M.; Yoshina-Ishii C.; Chi W.; Brown R. S.; Batey R. A. Carbamoylimidazolium and Thiocarbamoylimidazolium Salts: Novel Reagents for the Synthesis of Ureas, Thioureas, Carbamates, Thiocarbamates and Amides. Tetrahedron 2005, 61 (30), 7153–7175. 10.1016/j.tet.2005.05.056.

[ref29] Bansagi J.; Wilson-Konderka C.; Debrauwer V.; Narayanan P.; Batey R. A. N-Alkyl Carbamoylimidazoles as Isocyanate Equivalents: Exploration of the Reaction Scope for the Synthesis of Ureas, Hydantoins, Carbamates, Thiocarbamates, and Oxazolidinones. J. Org. Chem. 2022, 87 (17), 11329–11349. 10.1021/acs.joc.2c00803. 35968929

[ref30] Staab H. A.; Wendel K.; Datta A. P. Carbonsäurechloride durch direkte Acylierung von Chlorwasserstoff mit Imidazoliden. Justus Liebigs Ann. Chem. 1966, 694 (1), 78–85. 10.1002/jlac.19666940110.

[ref31] Passia M. T.; Demaerel J.; Amer M. M.; Drichel A.; Zimmer S.; Bolm C. Acid-Mediated Imidazole-to-Fluorine Exchange for the Synthesis of Sulfonyl and Sulfonimidoyl Fluorides. Org. Lett. 2022, 24 (48), 8802–8805. 10.1021/acs.orglett.2c03546. 36417547

[ref32] Uematsu N.; Hoshi N.; Ikeda M. New Preparation of 1,1,2,2-Tetrafluoro-2-(Trifluoroethenyloxy)-Ethanesulfonyl Fluoride. J. Fluorine Chem. 2006, 127 (12), 1595–1600. 10.1016/j.jfluchem.2006.09.005.

[ref33] Kwon Y.-D.; Jeon M. H.; Park N. K.; Seo J. K.; Son J.; Ryu Y. H.; Hong S. Y.; Chun J.-H. Synthesis of 18F-Labeled Aryl Fluorosulfates via Nucleophilic Radiofluorination. Org. Lett. 2020, 22 (14), 5511–5516. 10.1021/acs.orglett.0c01868. 32589035

[ref34] Chou T. S.; Becke L. M.; O’Toole J. C.; Carr M. A.; Parker B. E. Triethylamine Poly(Hydrogen Fluorides) in the Synthesis of a Fluorinated Nucleoside Glycon. Tetrahedron Lett. 1996, 37 (1), 17–20. 10.1016/0040-4039(95)02102-7.

[ref35] Tsukamoto H.; Kahne D. N-Methylimidazolium Chloride-Catalyzed Pyrophosphate Formation: Application to the Synthesis of Lipid I and NDP-Sugar Donors. Bioorg. Med. Chem. Lett. 2011, 21 (17), 5050–5053. 10.1016/j.bmcl.2011.04.061. 21592792 PMC3156252

[ref36] Yanachkov I. B.; Dix E. J.; Yanachkova M. I.; Wright G. E. P1,P2-Diimidazolyl derivatives of pyrophosphate and bis-phosphonates – synthesis, properties, and use in preparation of dinucleoside tetraphosphates and analogs. Org. Biomol. Chem. 2011, 9 (3), 730–738. 10.1039/C0OB00542H. 21082127 PMC5705240

[ref37] Zhang H.; Wang Y.; Wang Y.; Li X.; Wang S.; Wang Z. Recent Advance on Carbamate-Based Cholinesterase Inhibitors as Potential Multifunctional Agents against Alzheimer’s Disease. Eur. J. Med. Chem. 2022, 240, 114606 10.1016/j.ejmech.2022.114606. 35858523

[ref38] Xing S.; Li Q.; Xiong B.; Chen Y.; Feng F.; Liu W.; Sun H. Structure and Therapeutic Uses of Butyrylcholinesterase: Application in Detoxification, Alzheimer’s Disease, and Fat Metabolism. Med. Res. Rev. 2021, 41 (2), 858–901. 10.1002/med.21745. 33103262

[ref39] Onida K.; Tlili A. Direct Synthesis of Carbamoyl Fluorides by CO2 Deoxyfluorination. Angew. Chem., Int. Ed. 2019, 58 (36), 12545–12548. 10.1002/anie.201907354. 31297908

[ref40] Gale P. A.; Hiscock J. R.; Lalaoui N.; Light M. E.; Wells N. J.; Wenzel M. Benzimidazole-Based Anion Receptors: Tautomeric Switching and Selectivity. Org. Biomol. Chem. 2012, 10 (30), 5909–5915. 10.1039/c1ob06800h. 22218531

[ref41] Cutugno S.; Martelli G.; Negro L.; Savoia D. The Reaction of β-Amino Alcohols with 1,1′-Carbonyldiimidazole – Influence of the Nitrogen Substituent on the Reaction Course. Eur. J. Org Chem. 2001, 2001 (3), 517–522. 10.1002/1099-0690(200102)2001:3<517::AID-EJOC517>3.0.CO;2-N.

[ref42] Meden A.; Knez D.; Malikowska-Racia N.; Brazzolotto X.; Nachon F.; Svete J.; Sałat K.; Grošelj U.; Gobec S. Structure-Activity Relationship Study of Tryptophan-Based Butyrylcholinesterase Inhibitors. Eur. J. Med. Chem. 2020, 208, 112766 10.1016/j.ejmech.2020.112766. 32919297

[ref43] Meden A.; Knez D.; Brazzolotto X.; Nachon F.; Dias J.; Svete J.; Stojan J.; Grošelj U.; Gobec S. From Tryptophan-Based Amides to Tertiary Amines: Optimization of a Butyrylcholinesterase Inhibitor Series. Eur. J. Med. Chem. 2022, 234, 114248 10.1016/j.ejmech.2022.114248. 35299116

[ref44] Meden A.; Knez D.; Brazzolotto X.; Modeste F.; Perdih A.; Pišlar A.; Zorman M.; Zorović M.; Denic M.; Pajk S.; Živin M.; Nachon F.; Gobec S. Pseudo-Irreversible Butyrylcholinesterase Inhibitors: Structure–Activity Relationships, Computational and Crystallographic Study of the *N*-Dialkyl *O*-Arylcarbamate Warhead. Eur. J. Med. Chem. 2023, 247, 115048 10.1016/j.ejmech.2022.115048. 36586299

