# Supplementary material for: Facile Synthesis of Carbamoyl Fluorides viaN-Carbamoylimidazole Activation
Source: ACS Omega. 2025 Feb 14;10(7):6908–17. doi: 10.1021/acsomega.4c09438 (PMC11866180; doi:10.1021/acsomega.4c09438)
Supplement: Supplementary file 1 — ao4c09438_si_001.pdf [file ao4c09438_si_001.pdf]

Facile synthesis of carbamoyl fluorides *via* *N*-carbamoylimidazole activation

Anže Meden, Damijan Knez, and Stanislav Gobec*

University of Ljubljana, Faculty of Pharmacy, Department of Pharmaceutical Chemistry. Aškerčeva 7, SI-1000 Ljubljana, Slovenia.
**e-mail: stanislav.gobec@ffa.uni-lj.si*

Supplementary Results

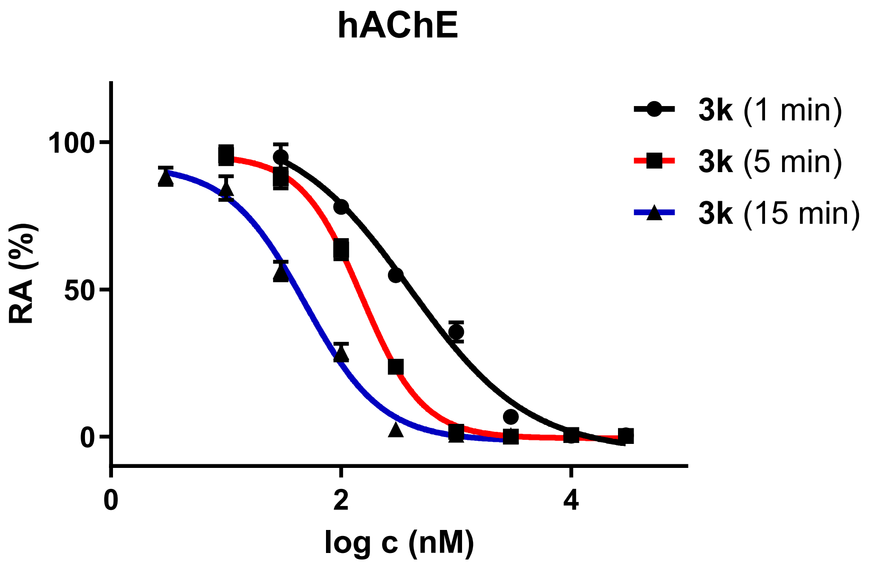

Figure S1. Time-dependency experiments on hAChE for compound 3k. The residual activities are plotted against log-concentration. The leftward shift, i.e., the decreasing of IC_{50} values with longer preincubation time, suggests covalent mechanism of inhibition.

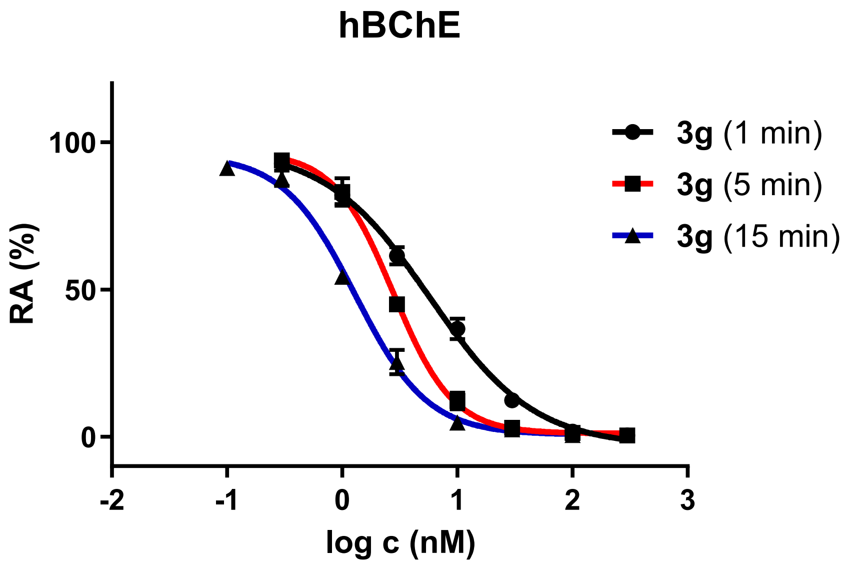

Figure S2. Time-dependency experiments on hBChE for compound 3g. The residual activities are plotted against log-concentration. The leftward shift, i.e., the decreasing of IC_{50} values with longer preincubation time, suggests covalent mechanism of inhibition.

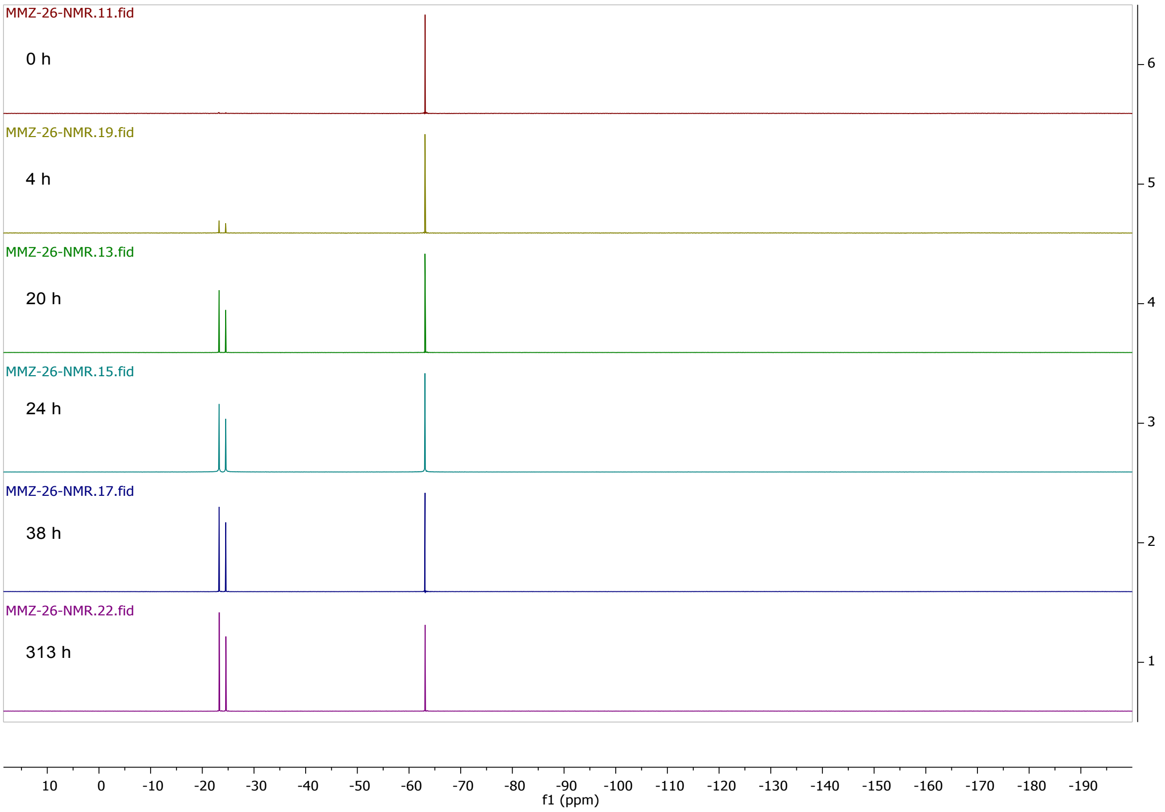

Figure S3. In an NMR tube, 0.3 mmol **2a**, 1.5 eq. MeI and 5 eq. KF, 5 µL of internal standard, and 600 µL MeCN-d_3 were mixed, the tube was left in an autosampler at room temperature, and product formation was followed over the course of several hours/days. Singlet at –62.74 ppm corresponds to internal standard signal, and the two signals around –24 ppm to carbamoyl fluoride product.

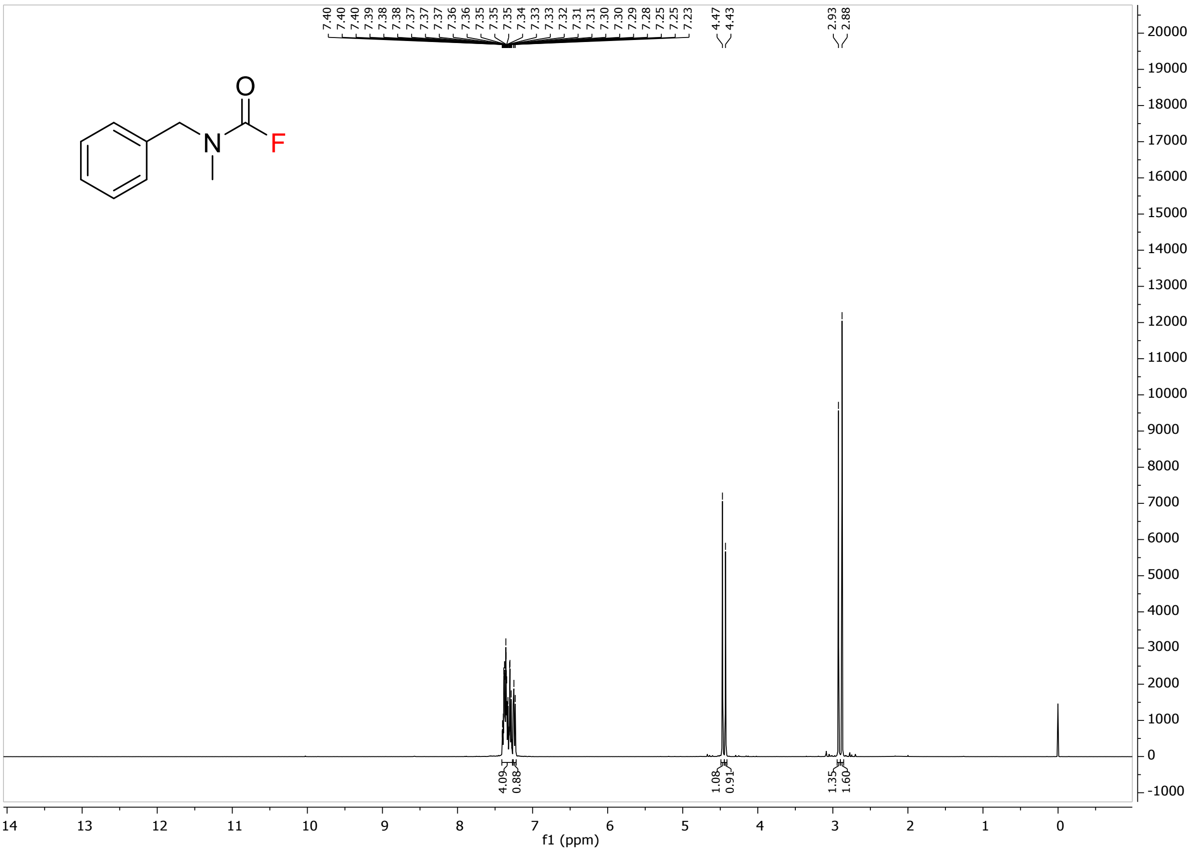

Figure S4. ^{1}H NMR spectrum of **3a**

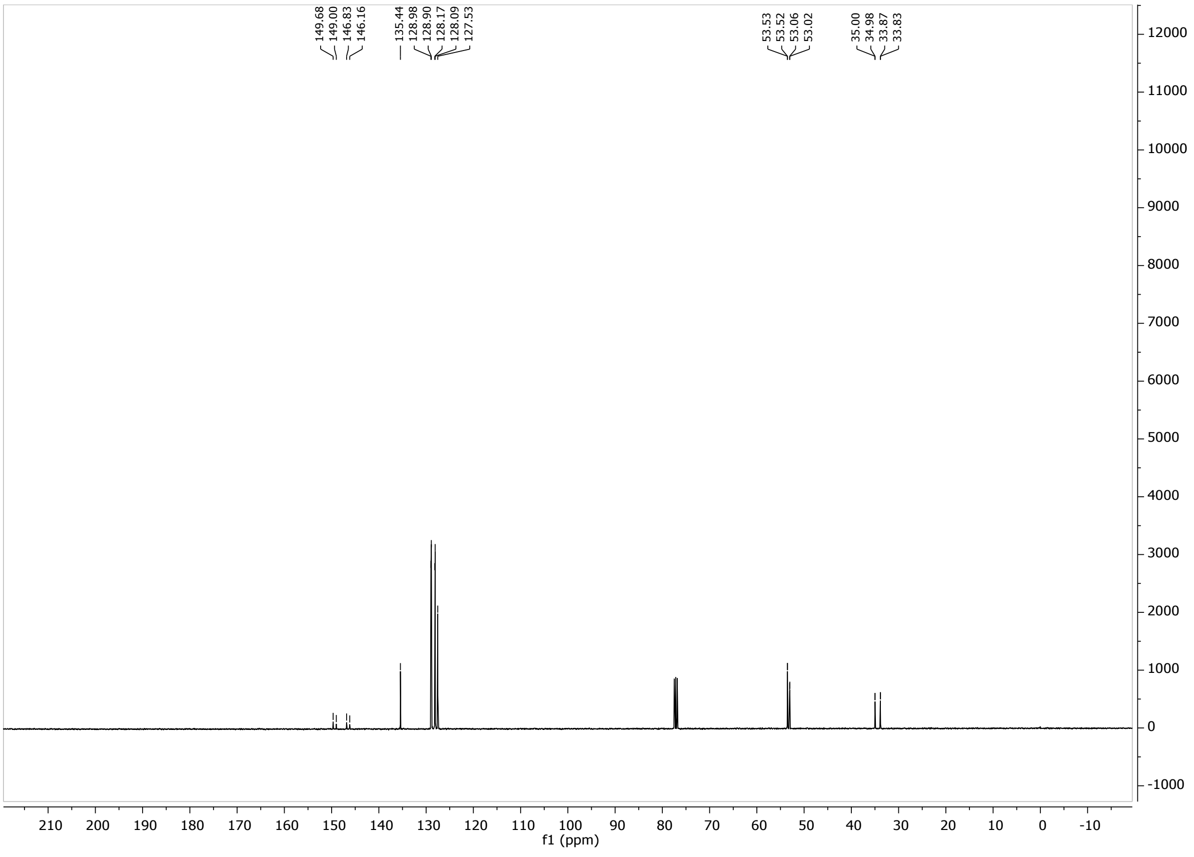

Figure S5. ^{13}C NMR spectrum of **3a**

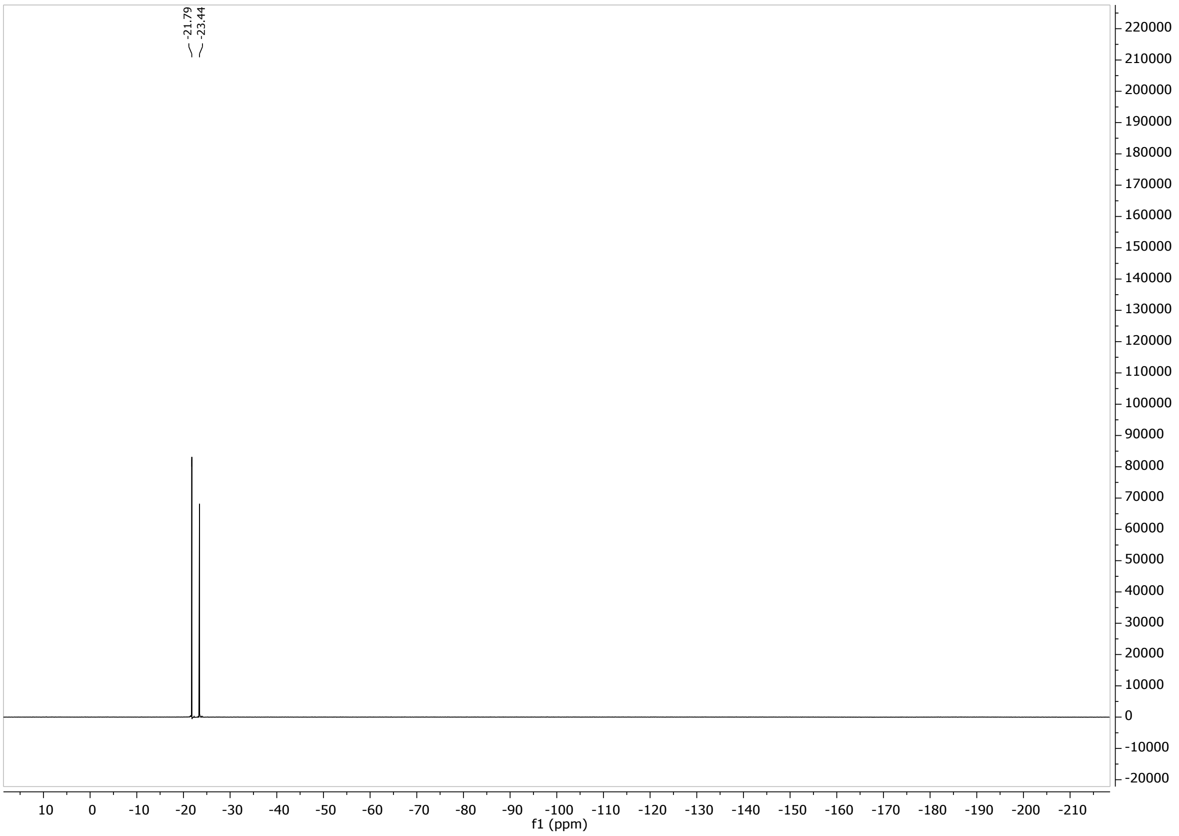

Figure S6. ^{19}F NMR spectrum of **3a**

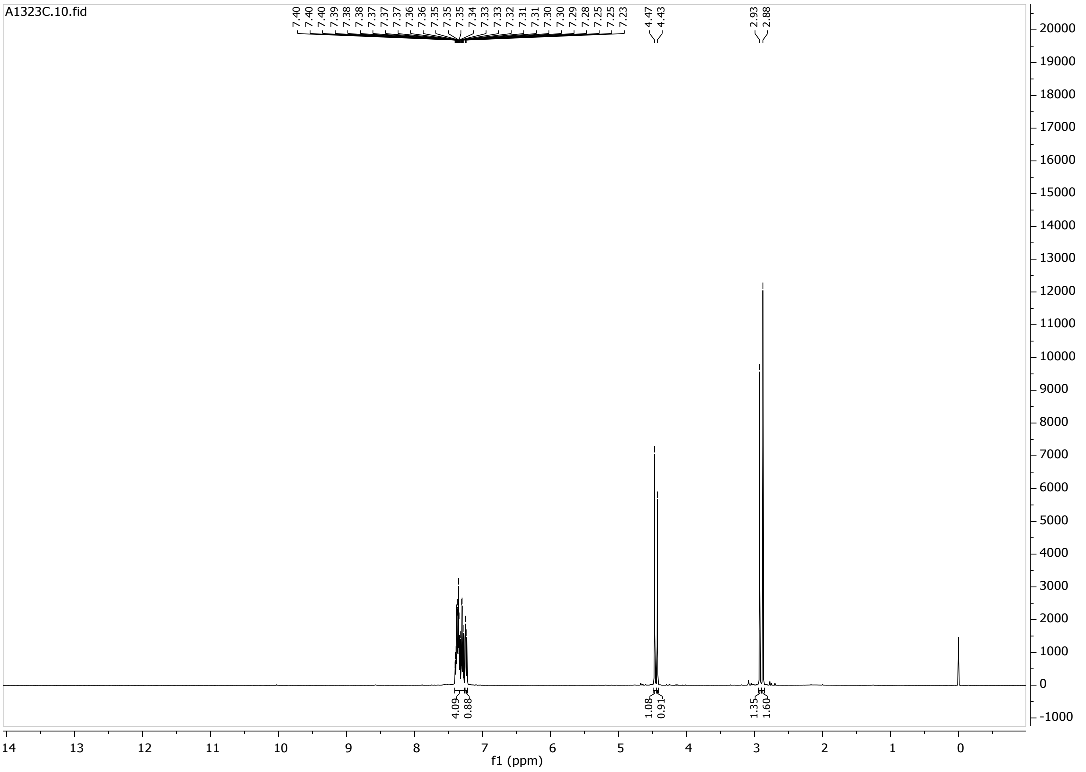

Figure S7. ^{1}H NMR spectrum of **3a**, entry 21 of Table 1

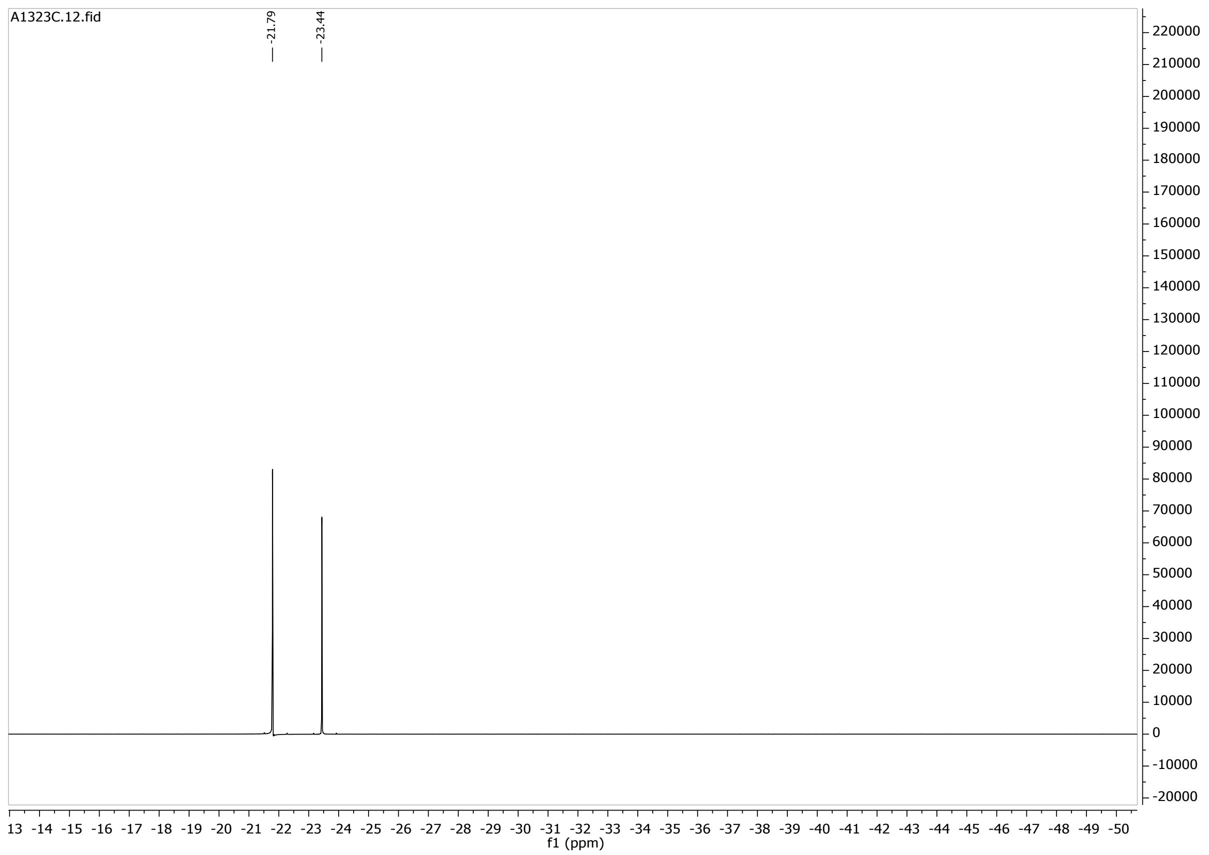

Figure S8. ^{19}F NMR spectrum of **3a**, entry 21 of Table 1

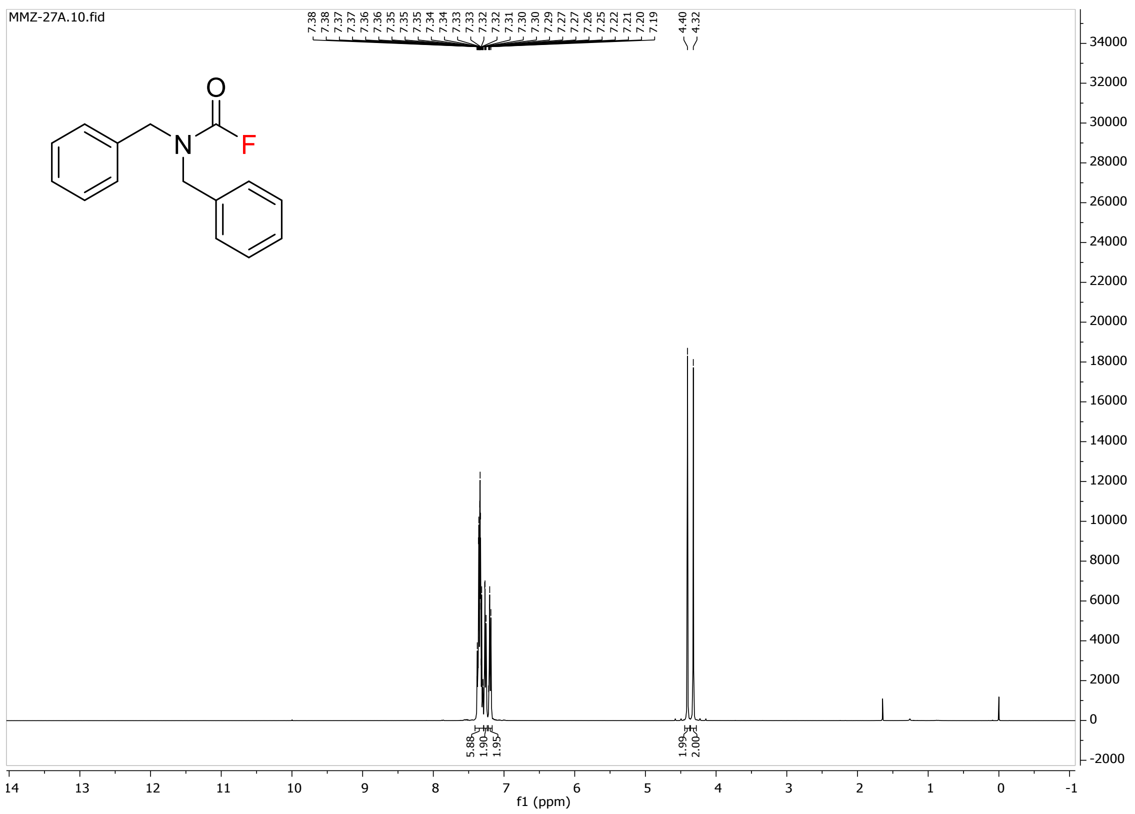

Figure S9. ^{1}H NMR spectrum of **3b**

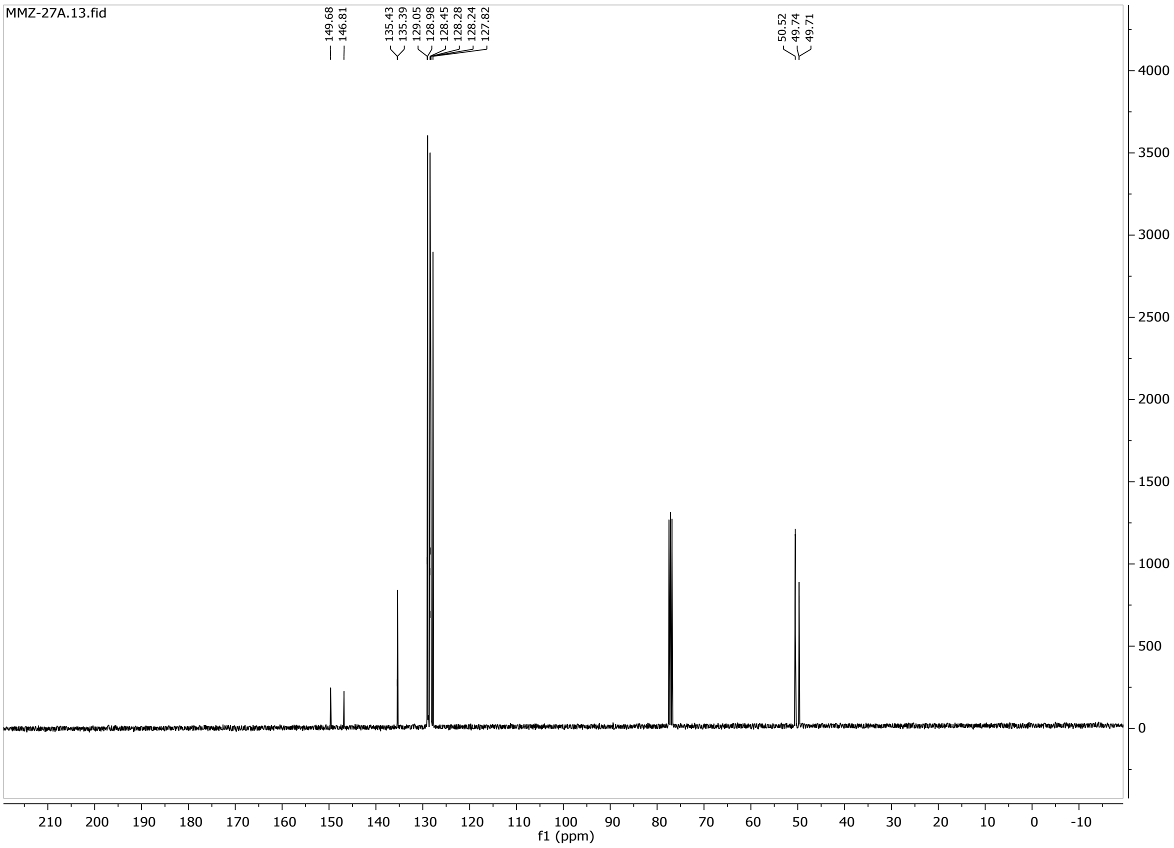

Figure S10. ^{13}C NMR spectrum of **3b**

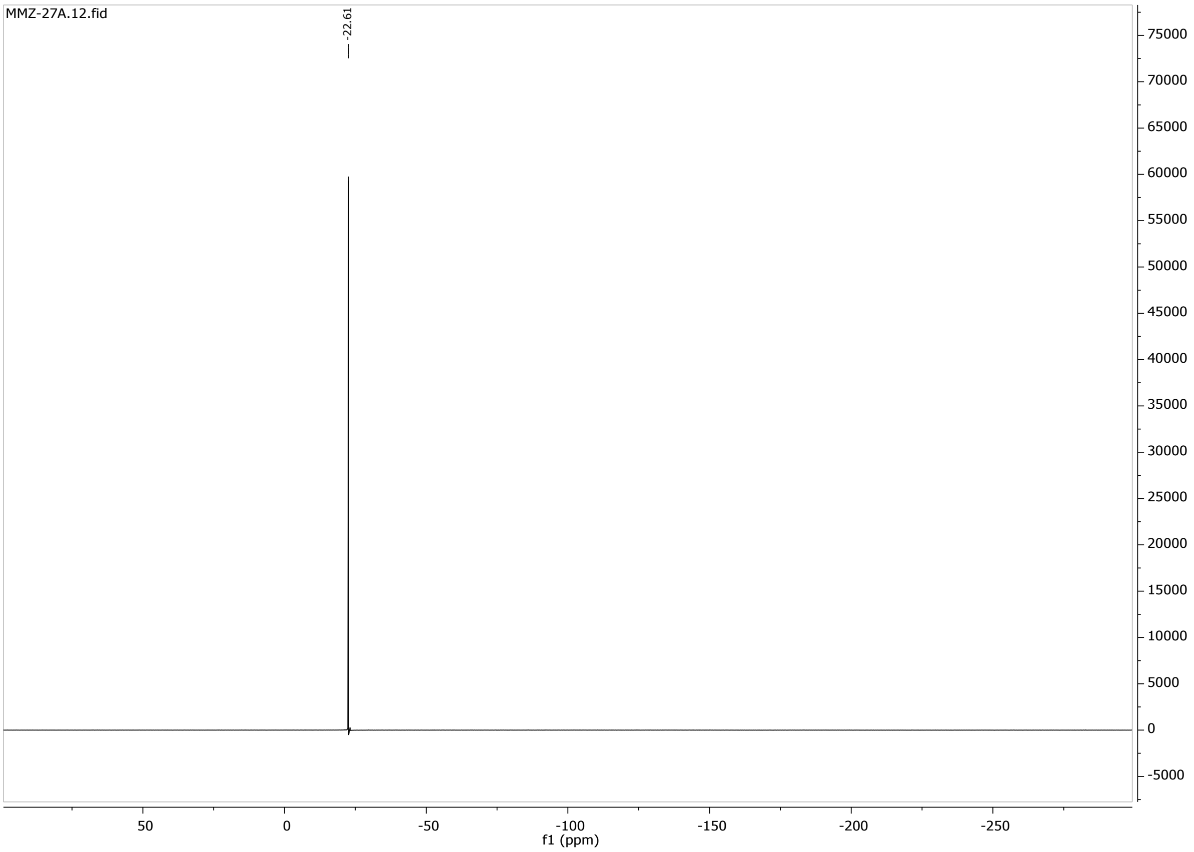

Figure S11. ^{19}F NMR spectrum of **3b**

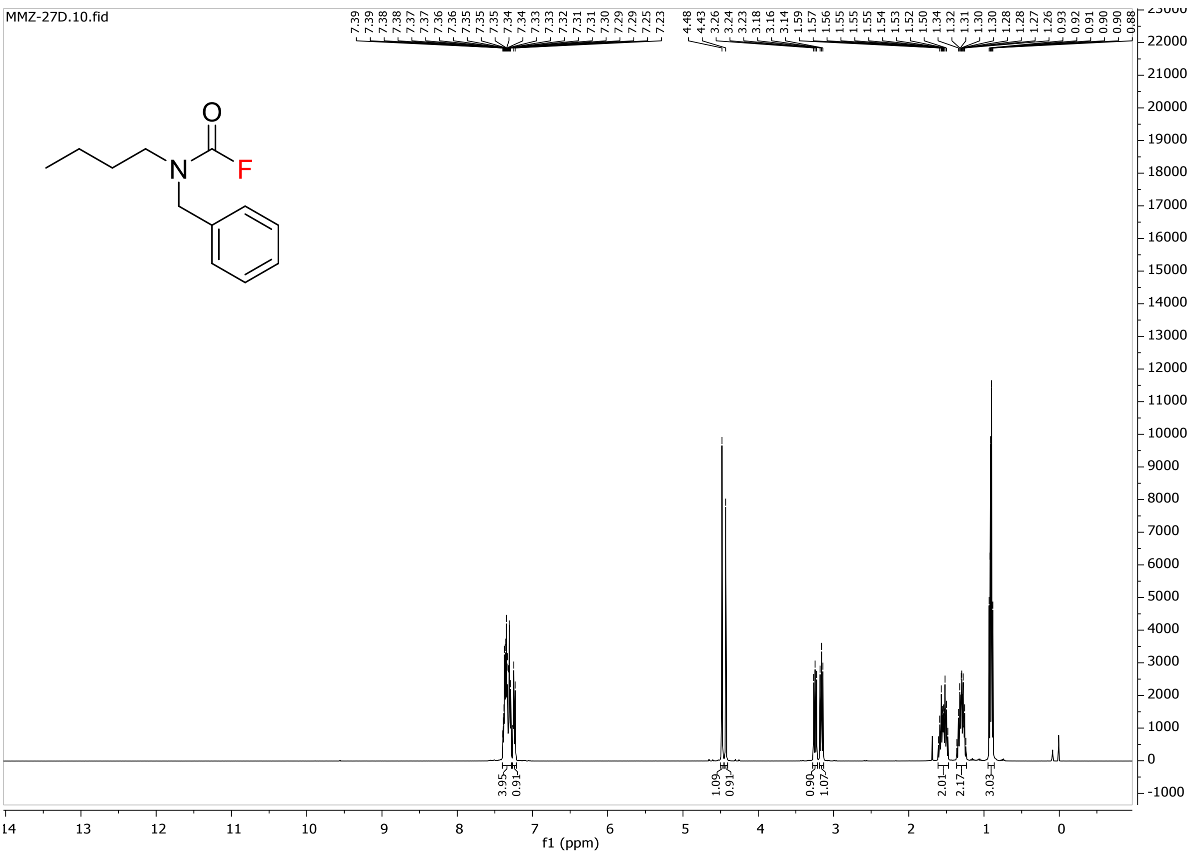

Figure S12. ^{1}H NMR spectrum of **3c**

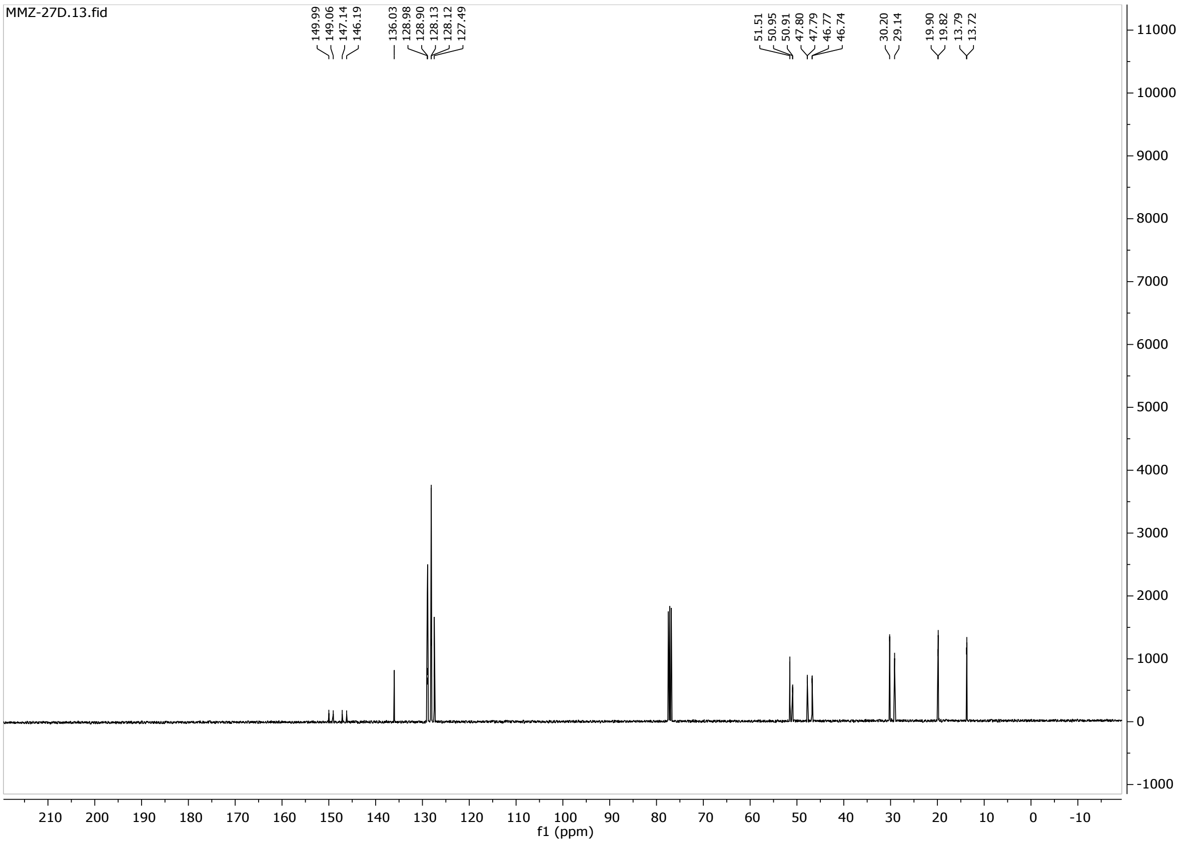

Figure S13. ^{13}C NMR spectrum of **3c**

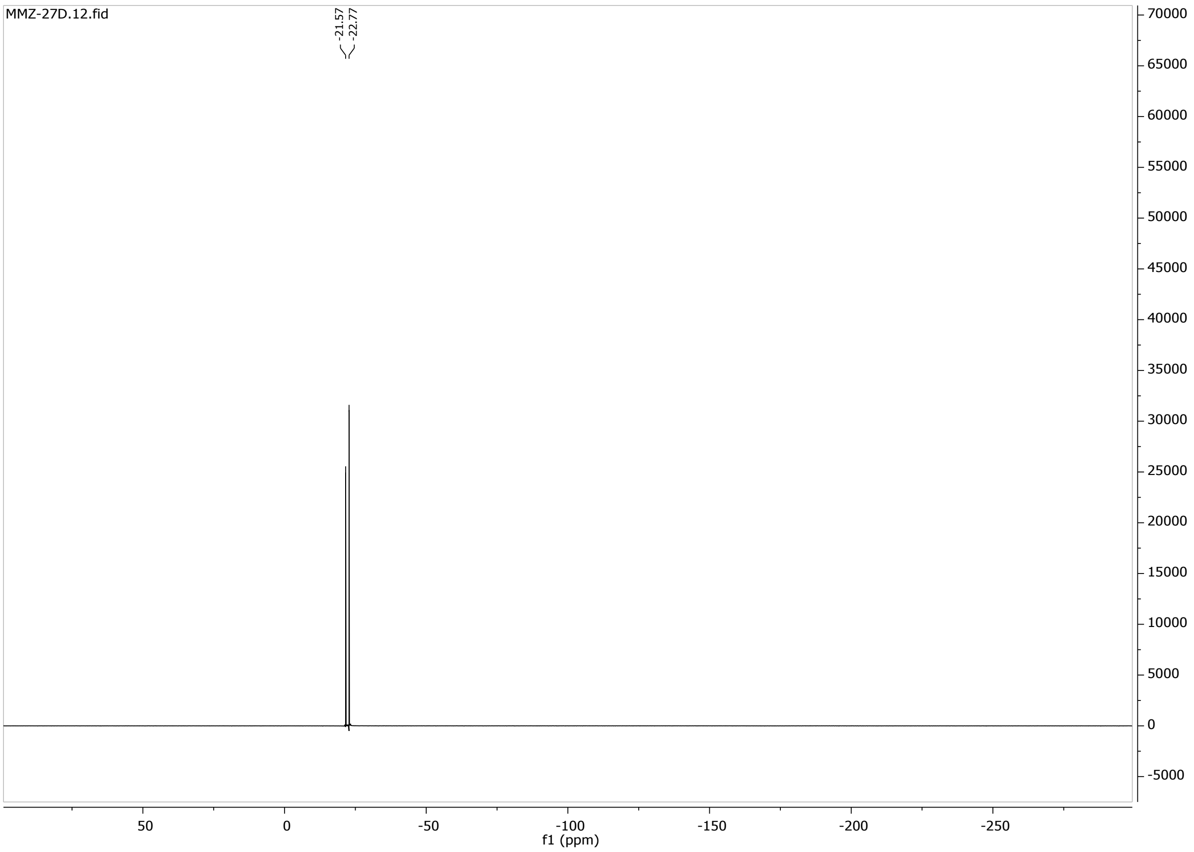

Figure S14. ^{19}F NMR spectrum of **3c**

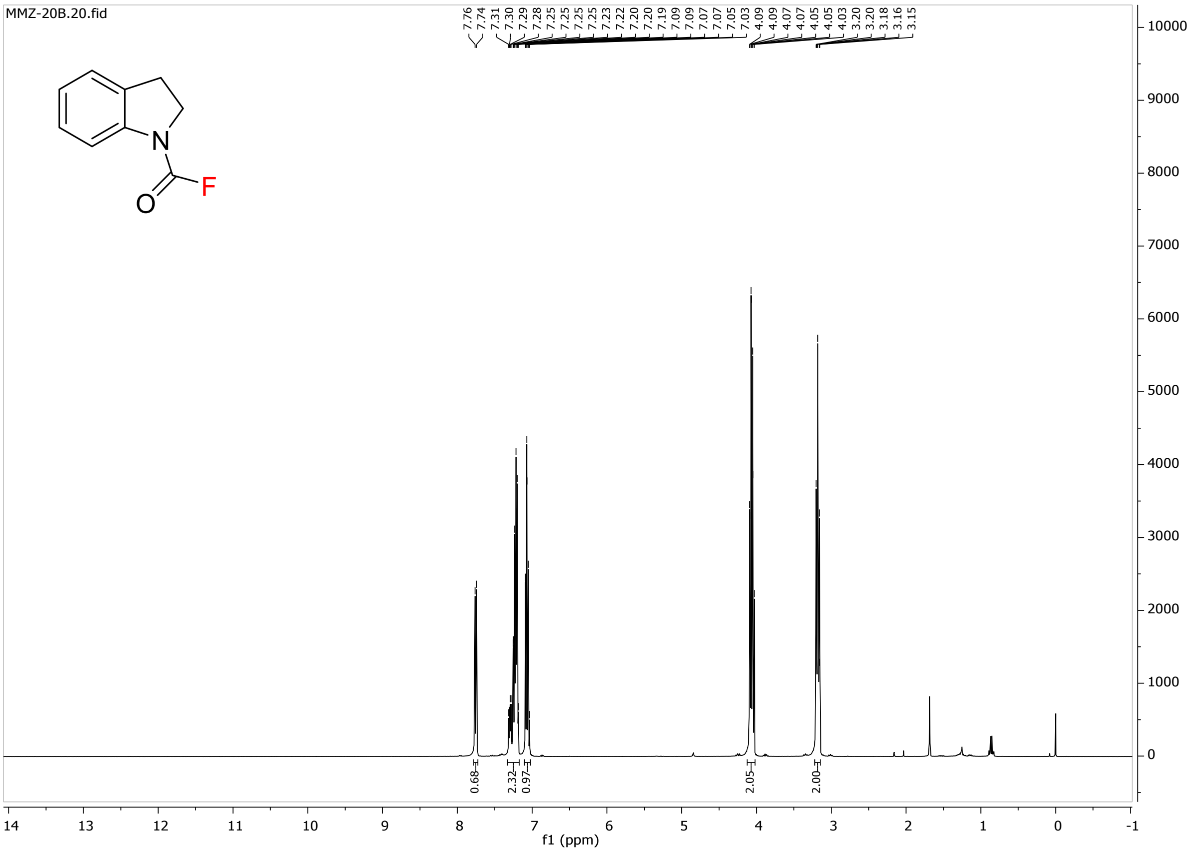

Figure S15. ^{1}H NMR spectrum of **3d**

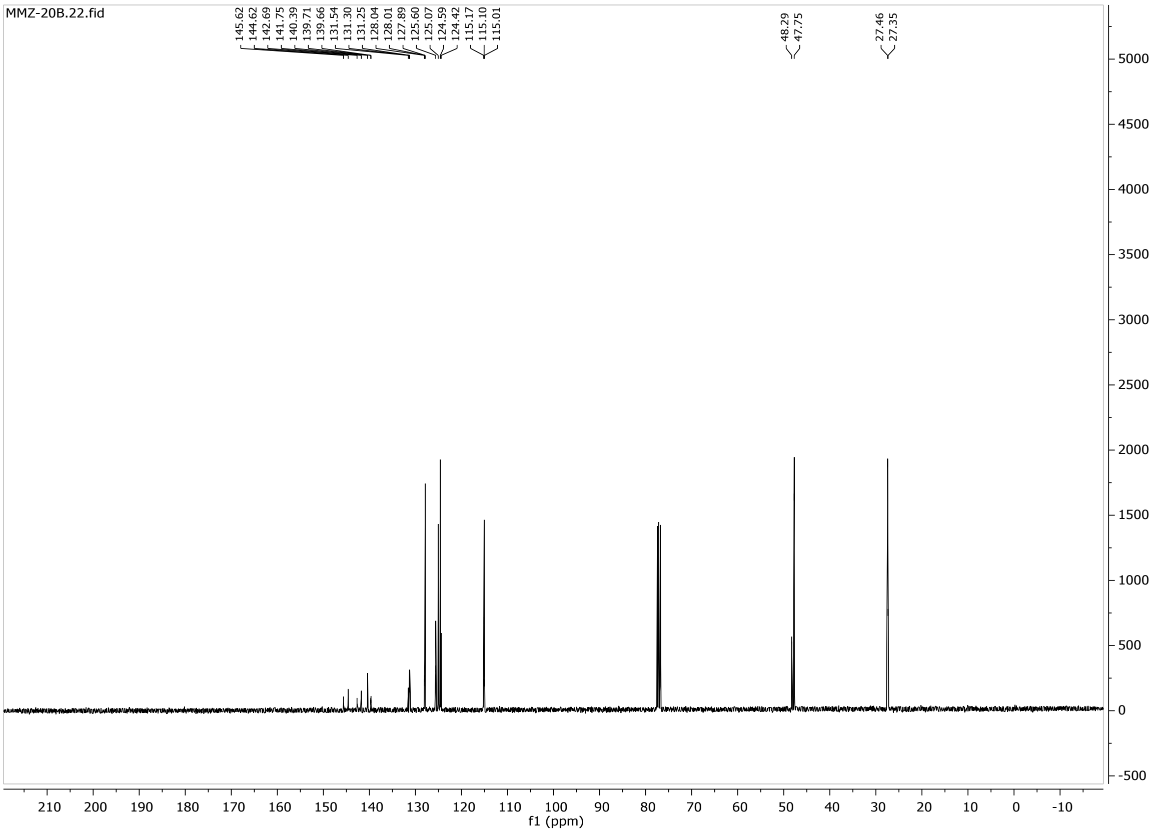

Figure S16. ^{13}C NMR spectrum of **3d**

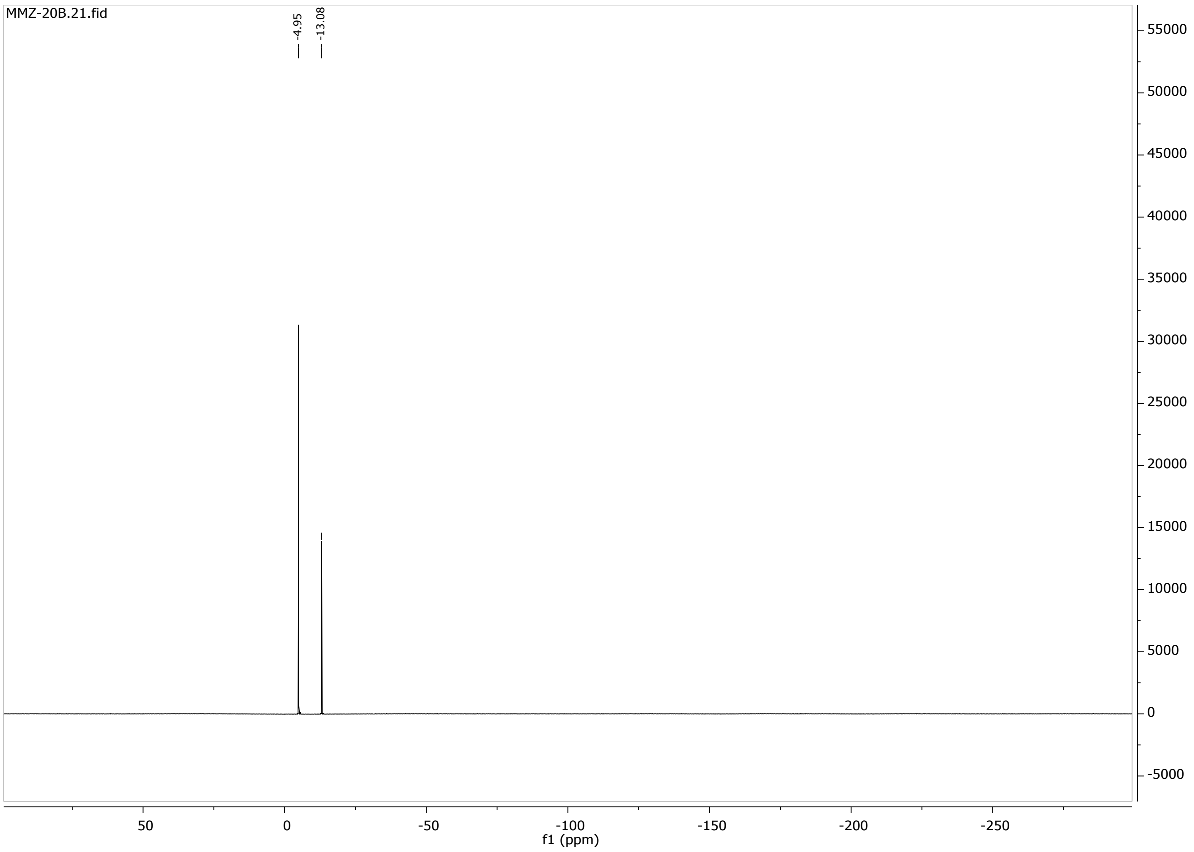

Figure S17. ^{19}F NMR spectrum of **3d**

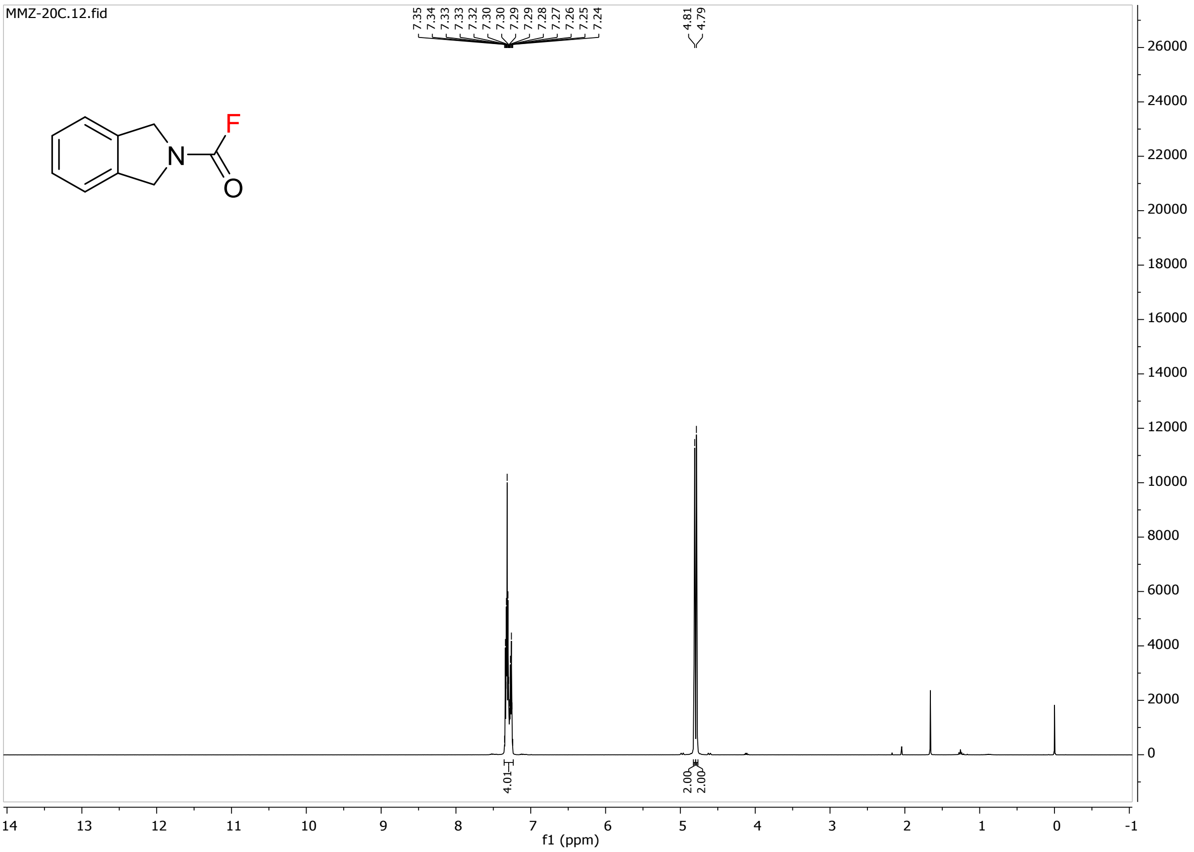

Figure S18. ^{1}H NMR spectrum of **3e**

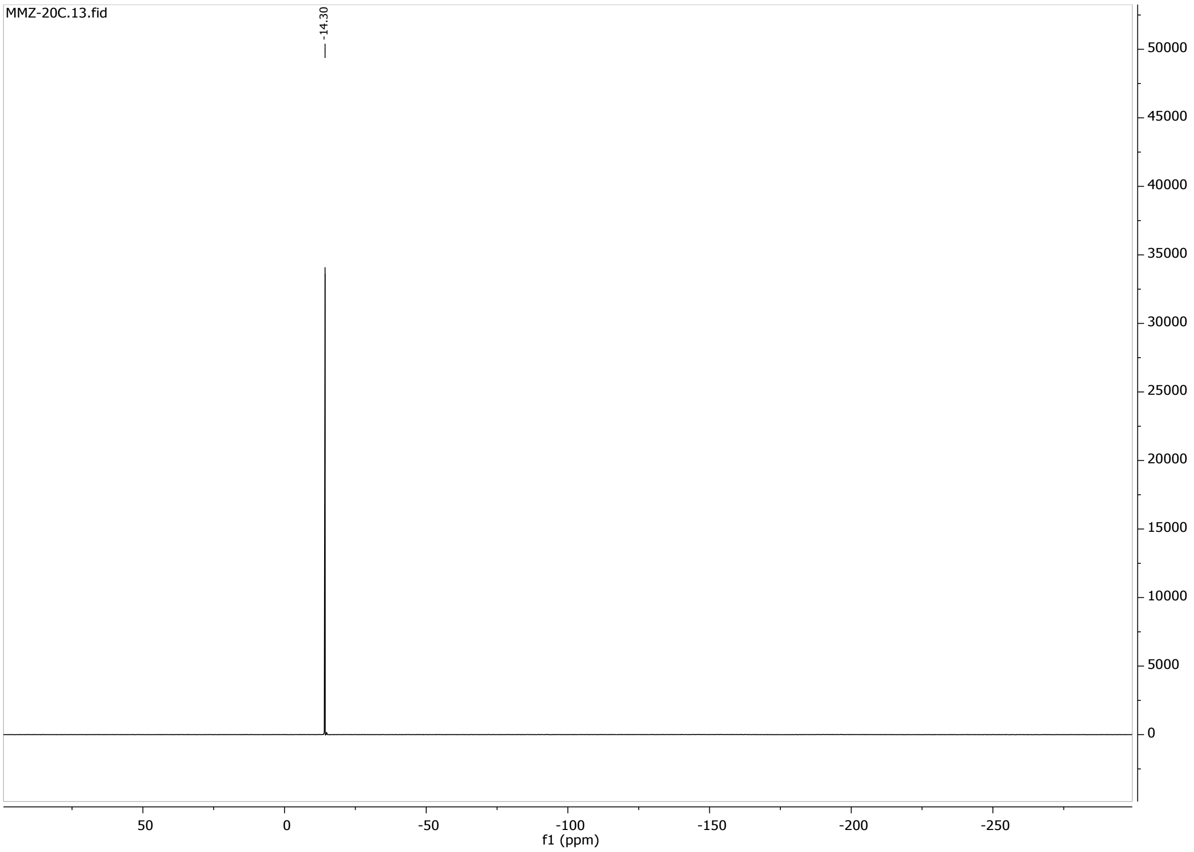

Figure S19. ^{19}F NMR spectrum of **3e**

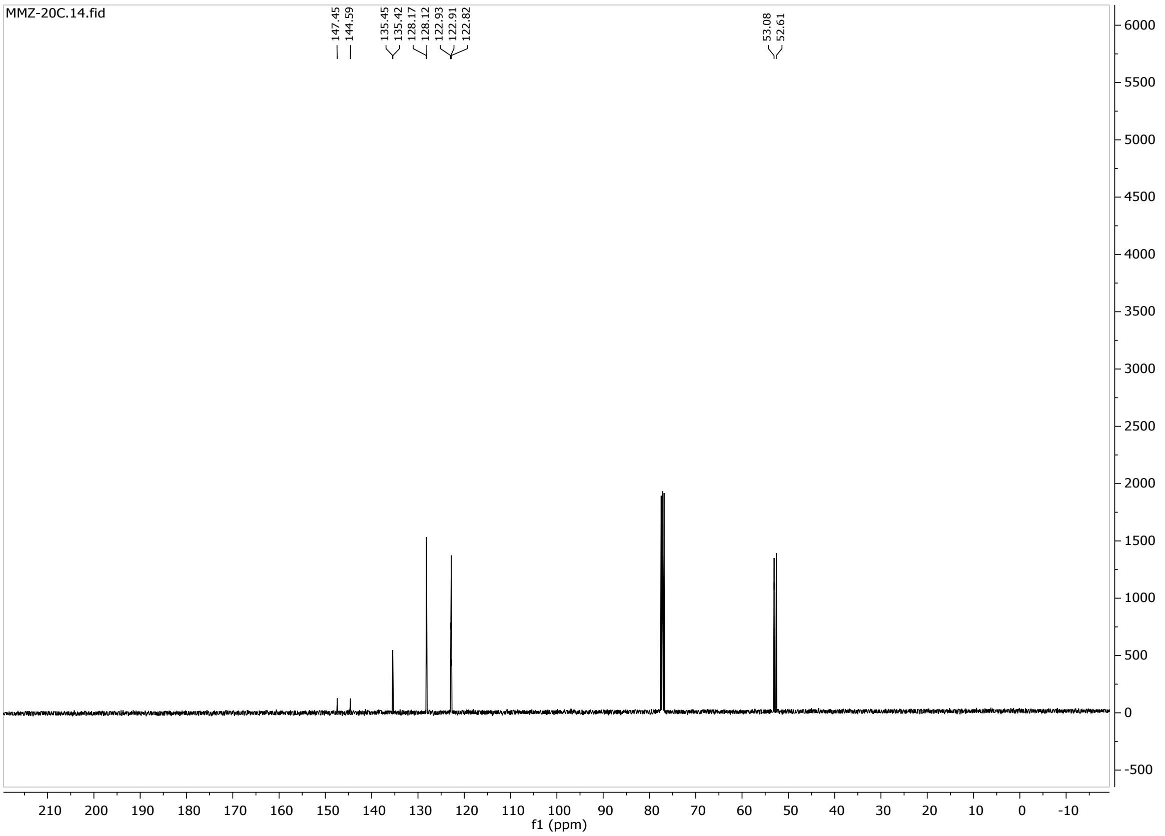

Figure S20. ^{13}C NMR spectrum of **3e**

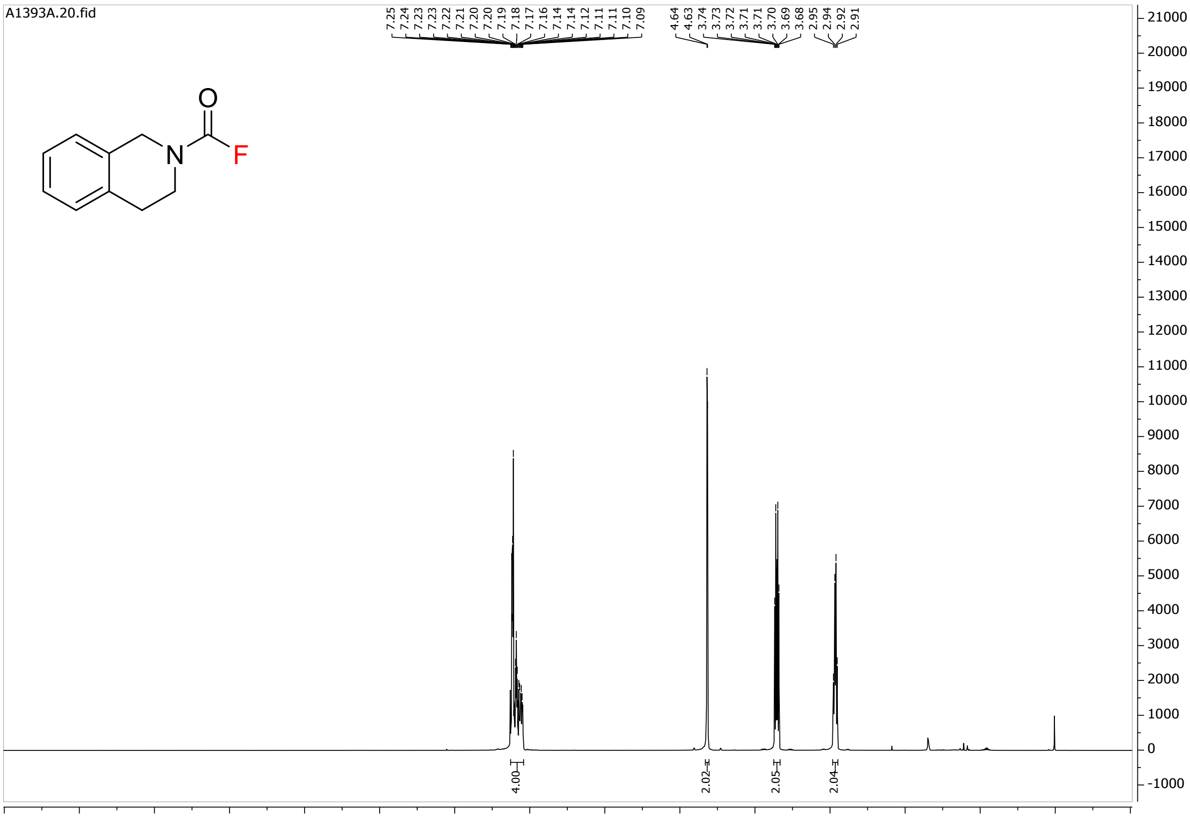

Figure S21. ^{1}H NMR spectrum of **3f**

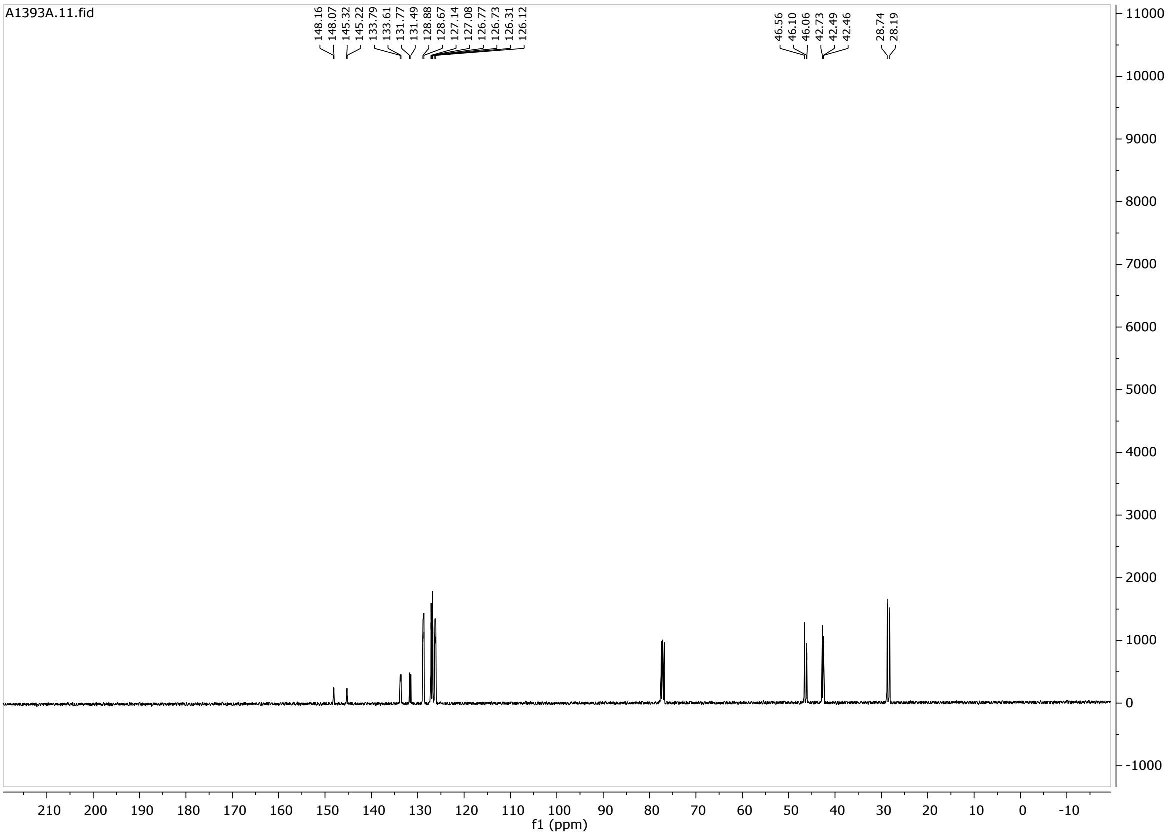

Figure S22. ^{13}C NMR spectrum of **3f**

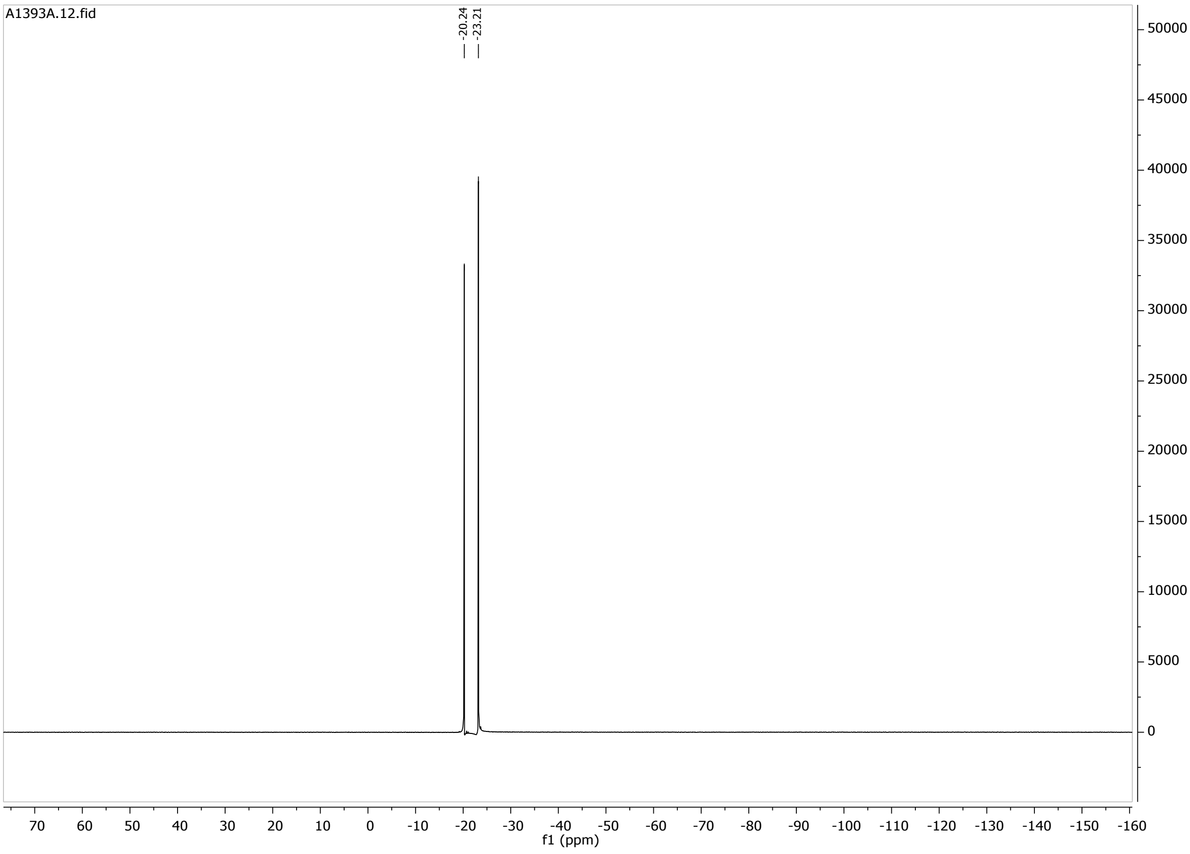

Figure S23. ^{19}F NMR spectrum of **3f**

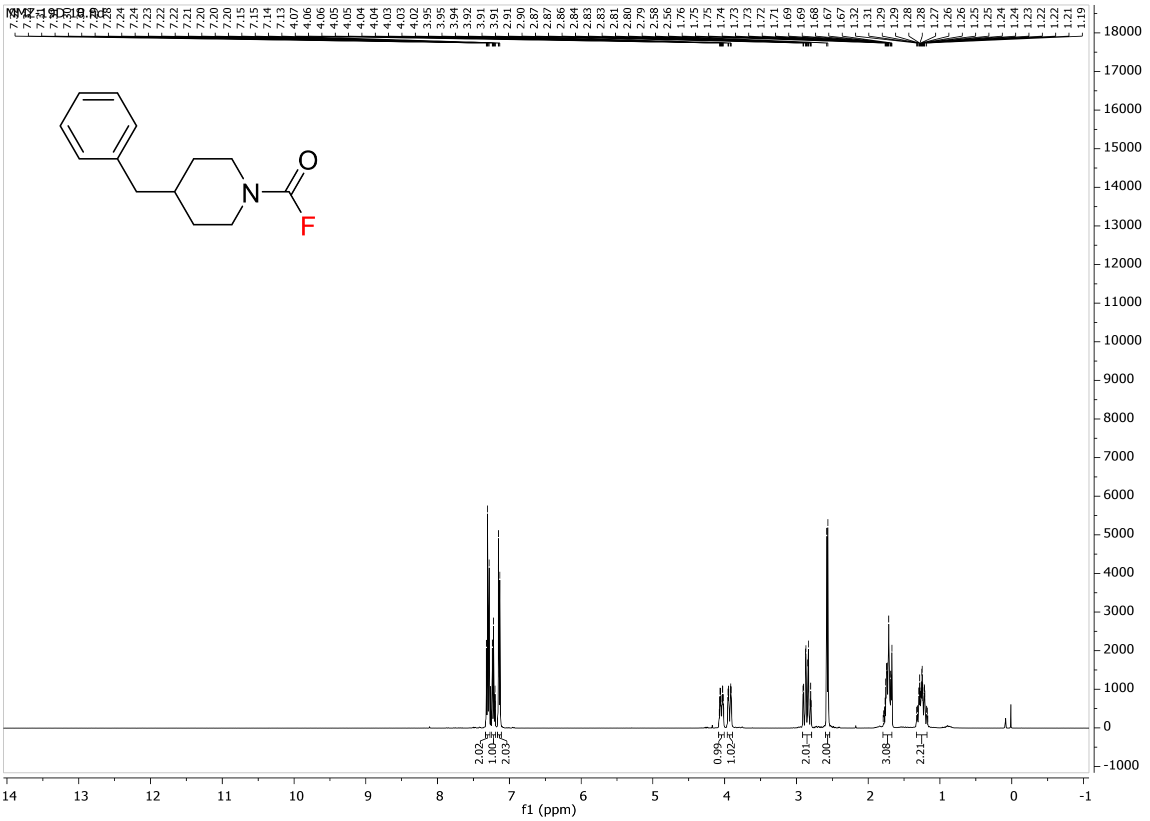

Figure S24. ^{1}H NMR spectrum of **3g**

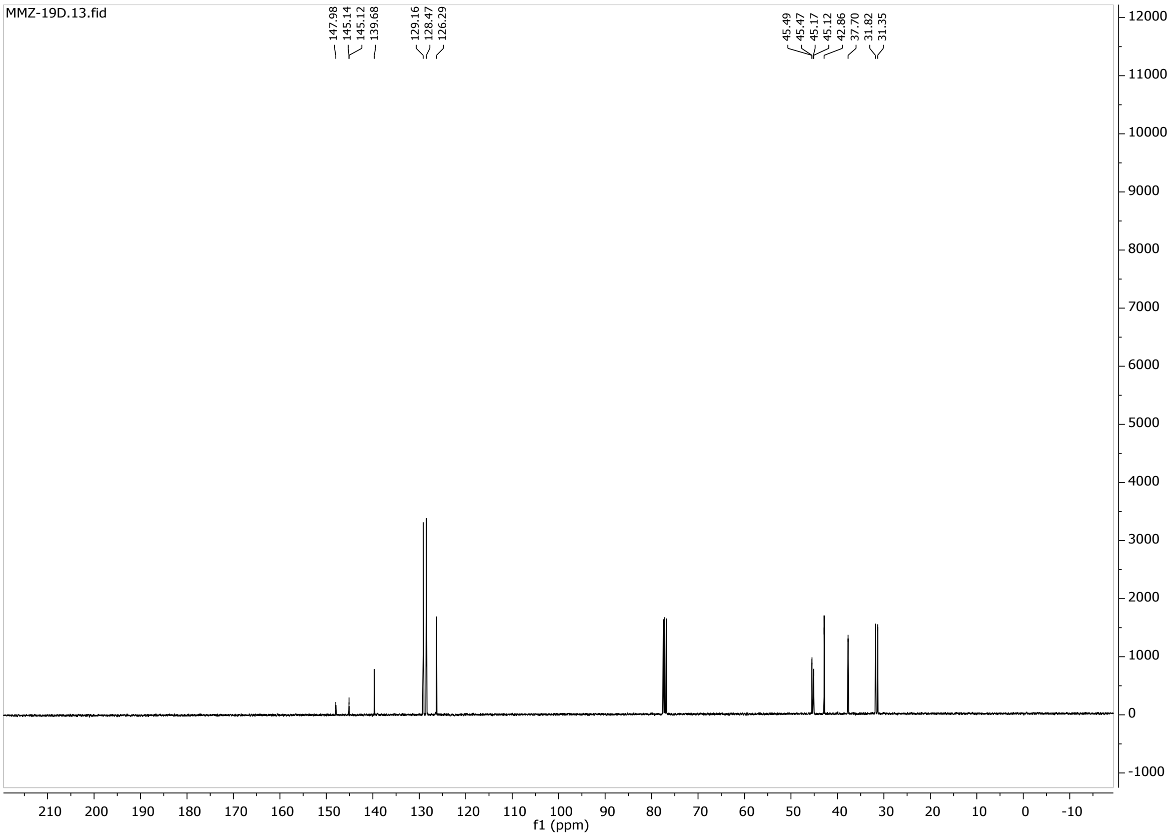

Figure S25. ^{13}C NMR spectrum of **3g**

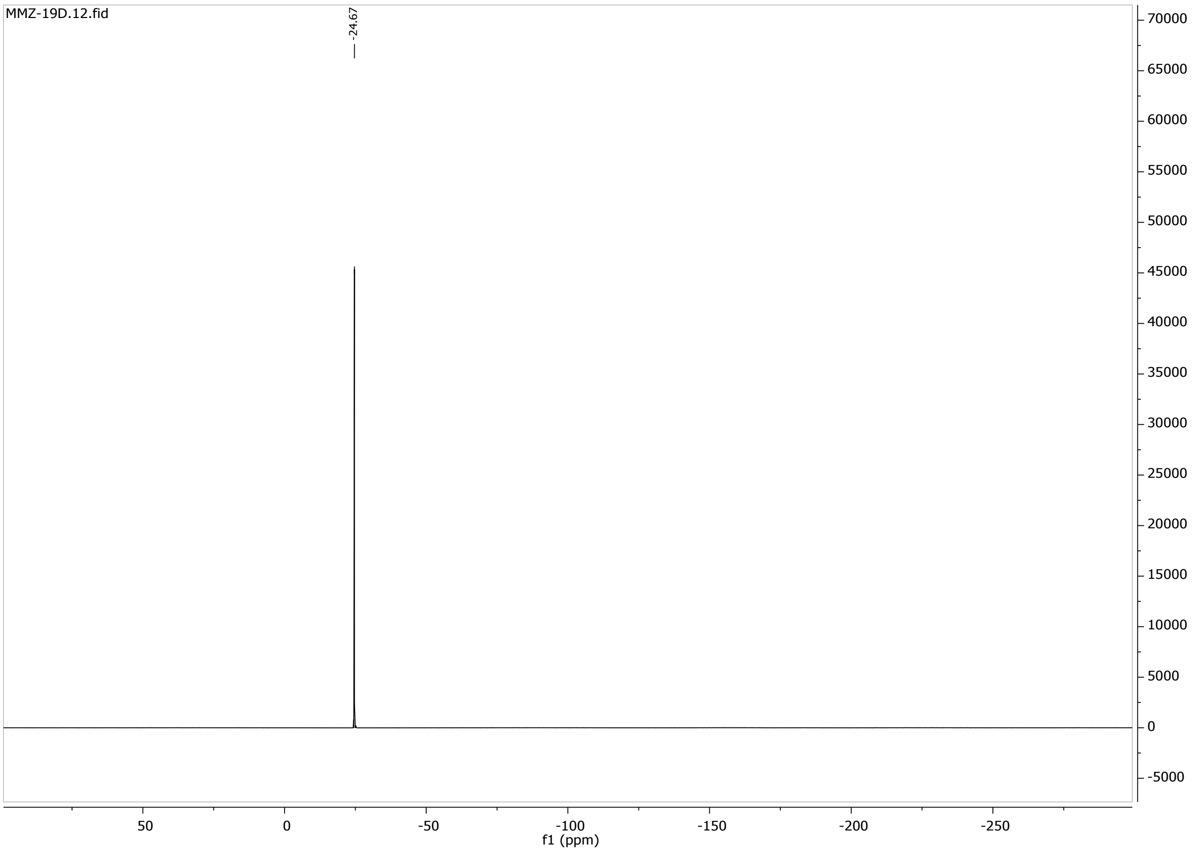

Figure S26. ^{19}F NMR spectrum of **3g**

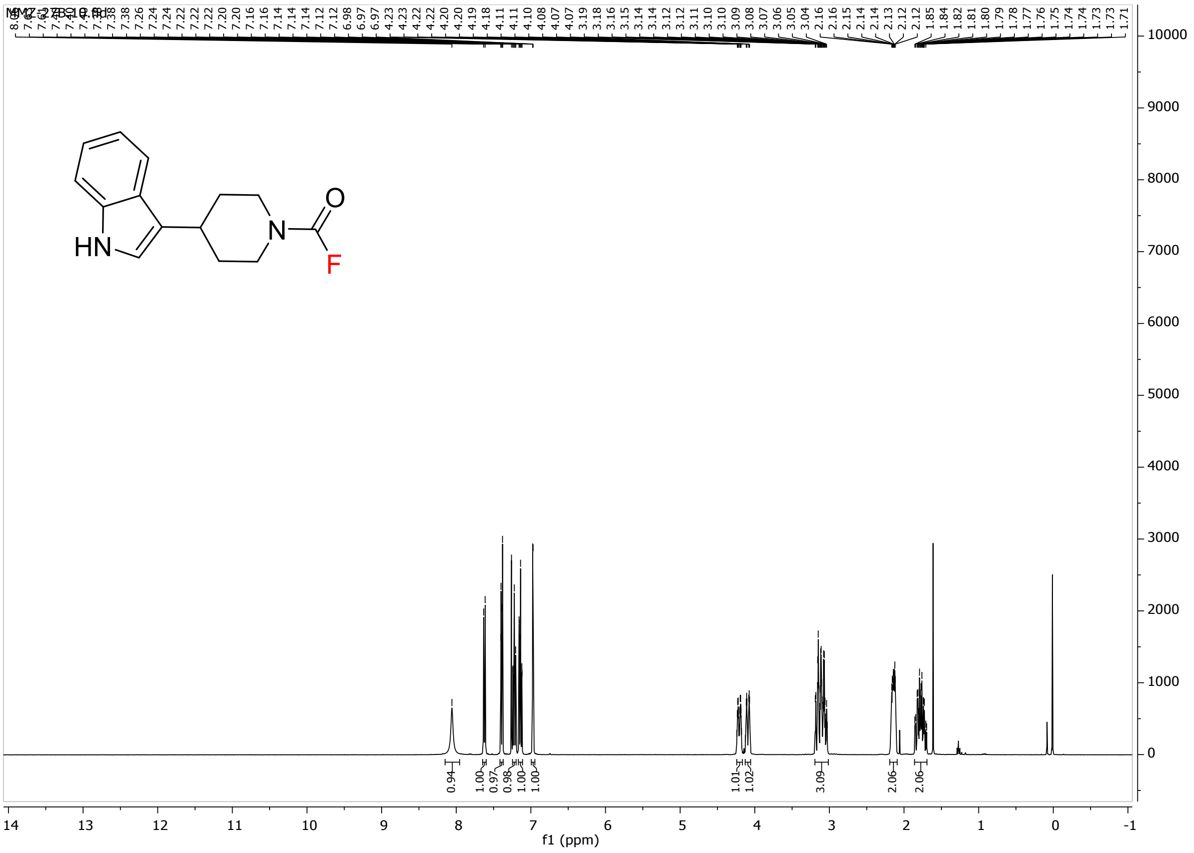

Figure S27. ^{1}H NMR spectrum of **3h**

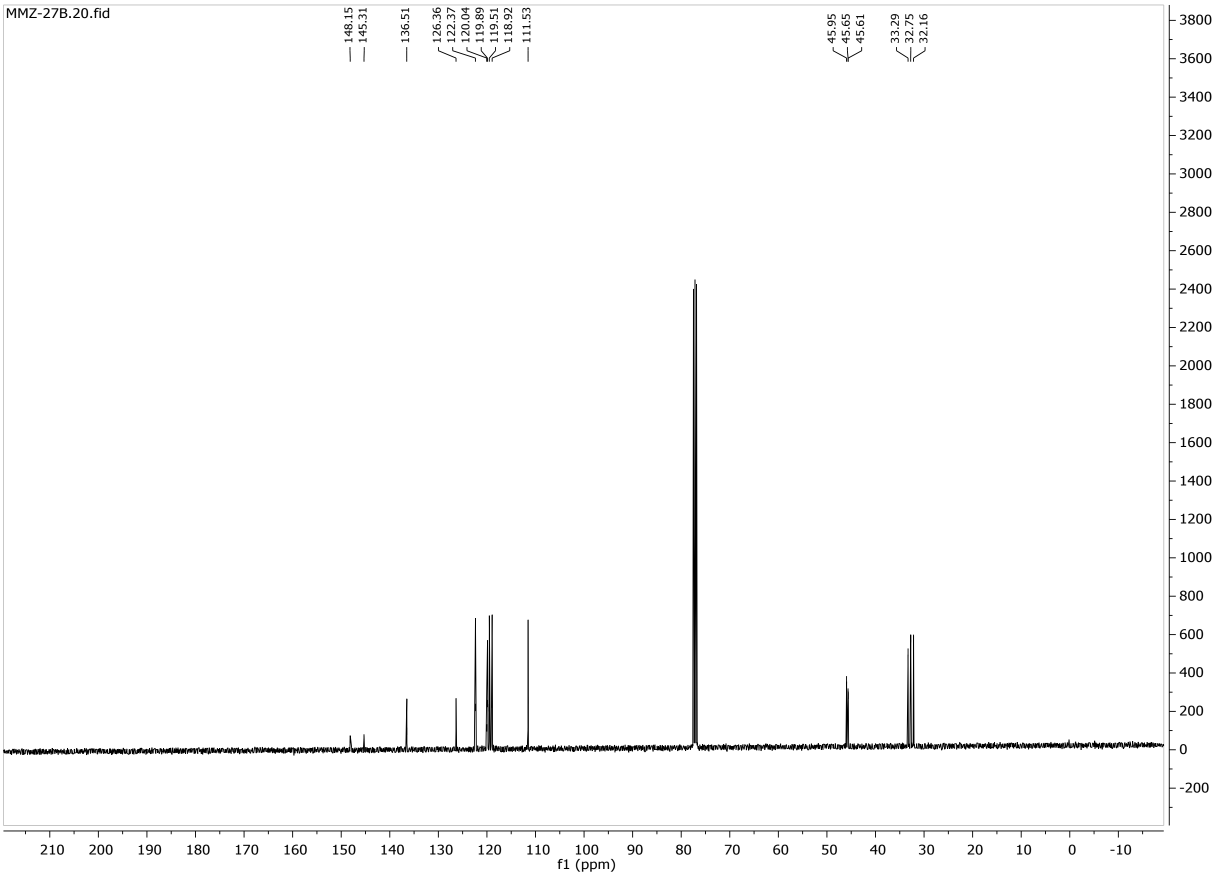

Figure S28. ^{13}C NMR spectrum of **3h**

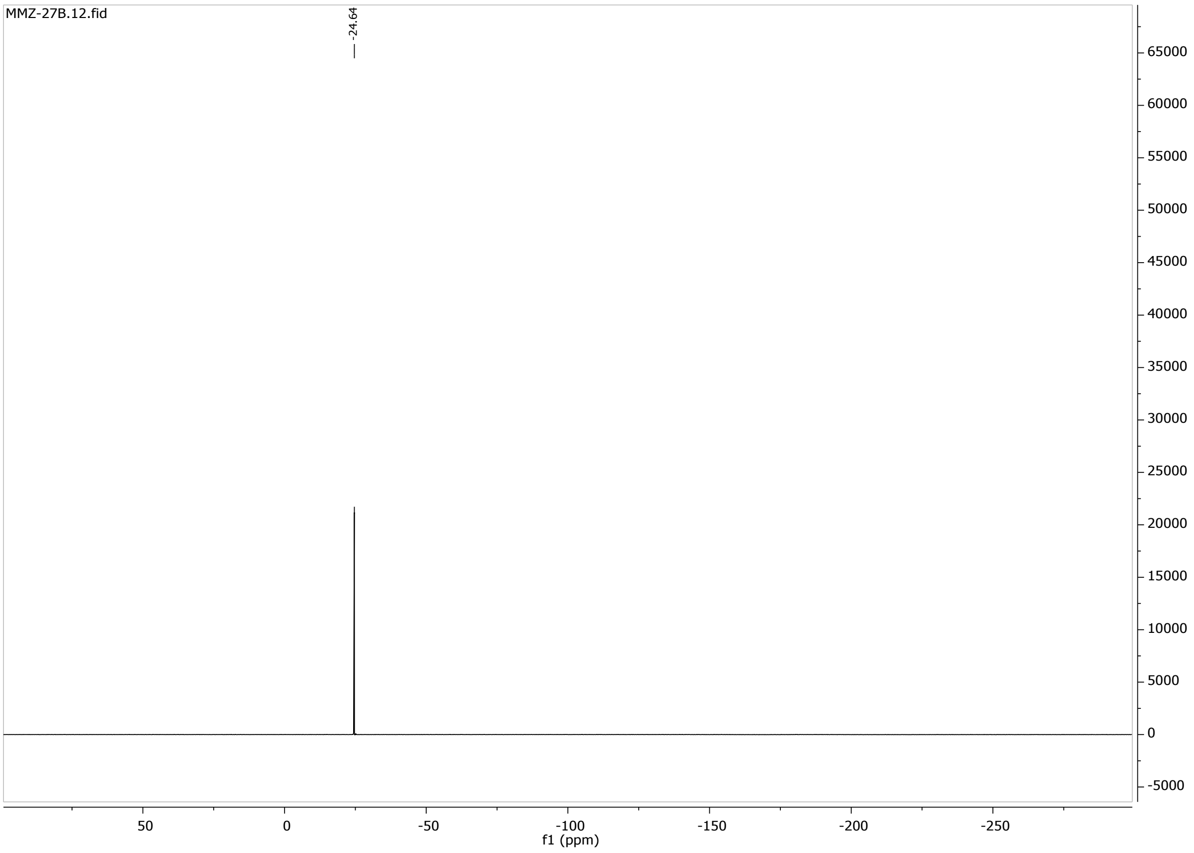

Figure S29. ^{19}F NMR spectrum of **3h**

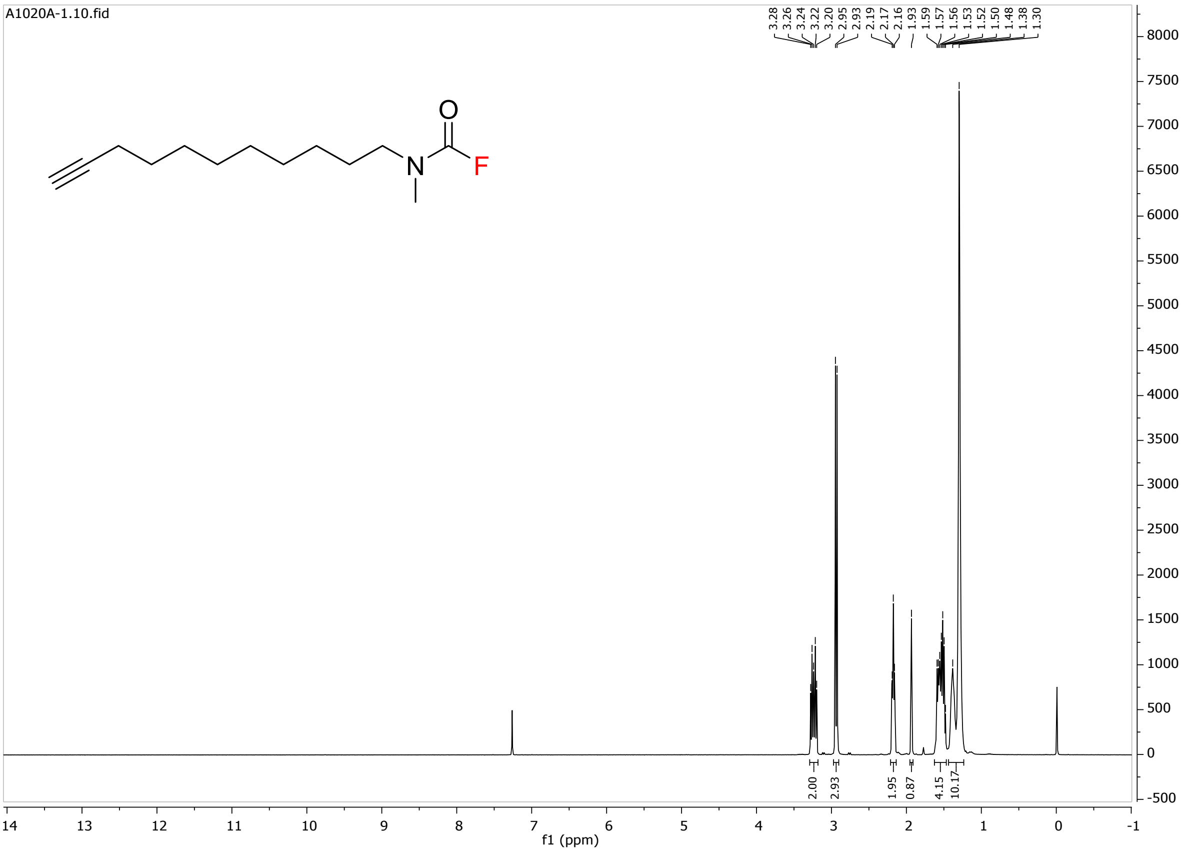

Figure S30. ^{1}H NMR spectrum of **3i**

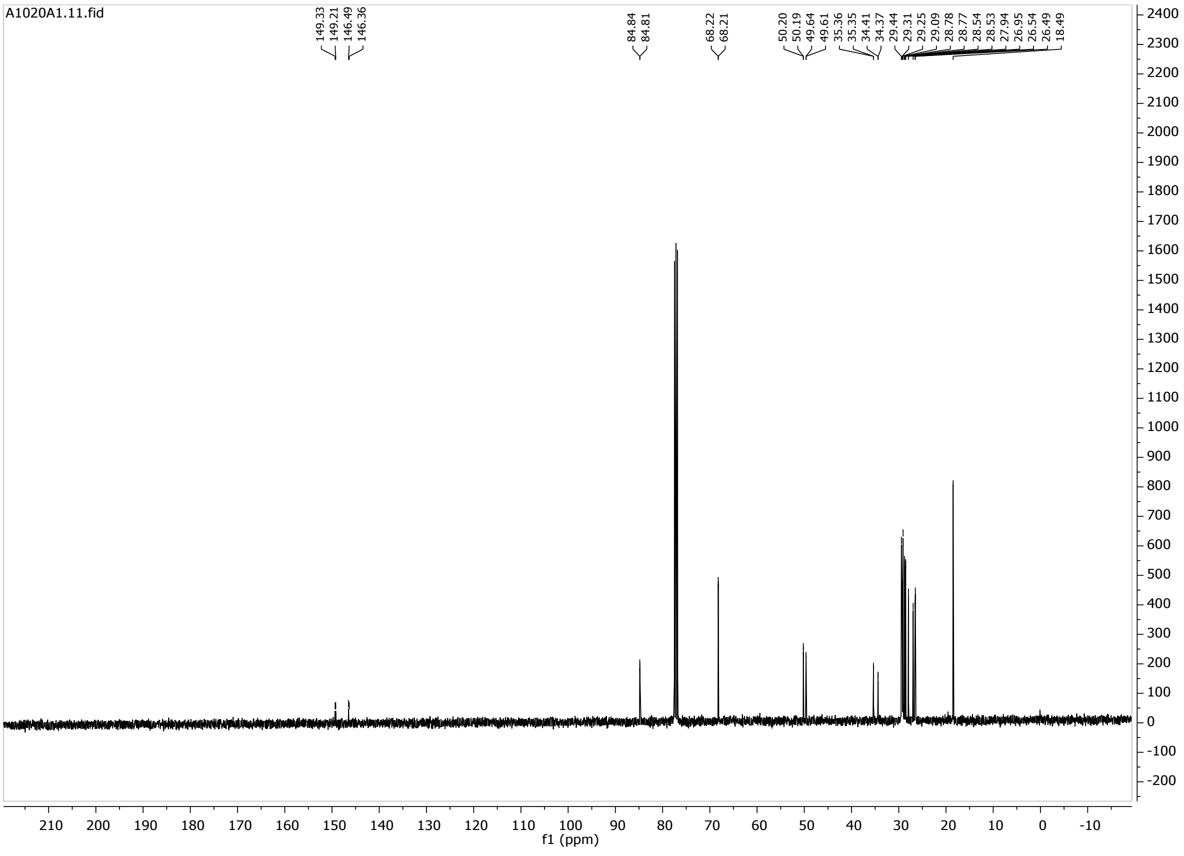

Figure S31. ^{13}C NMR spectrum of **3i**

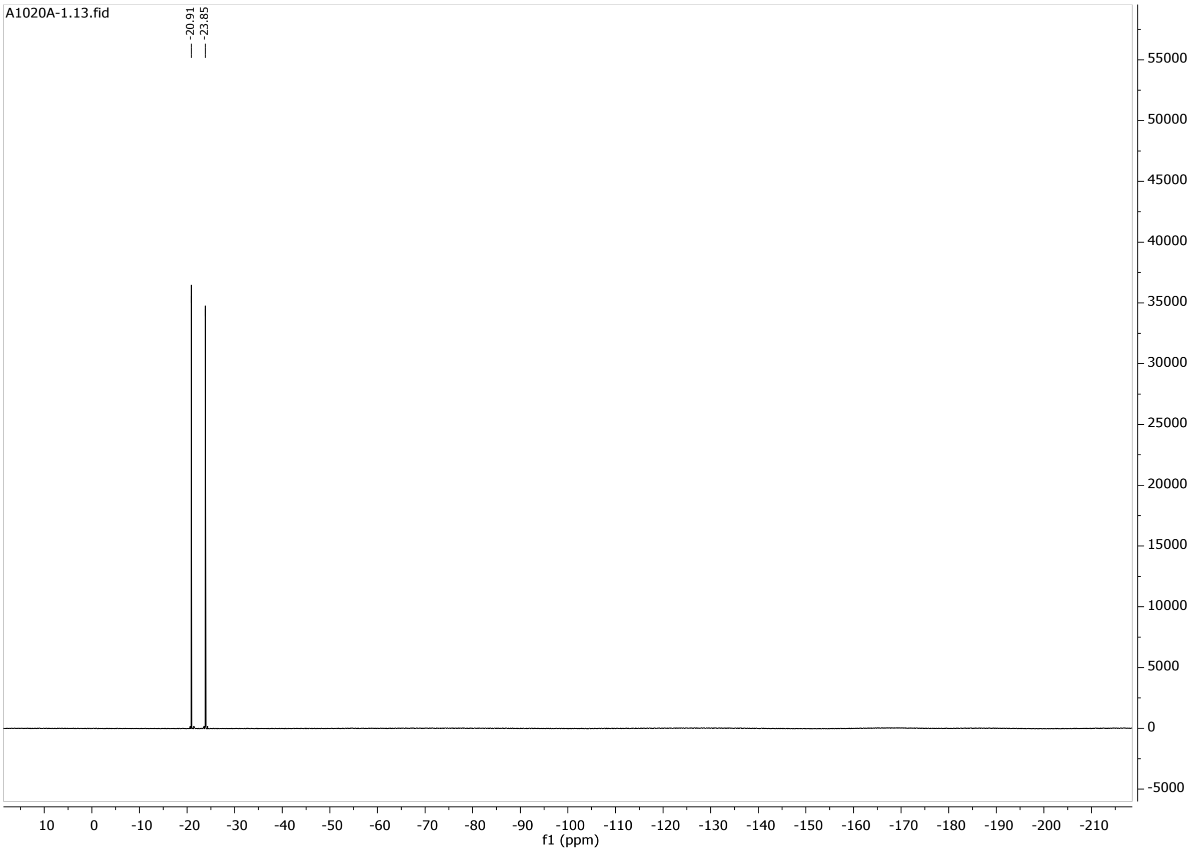

Figure S32. ^{19}F NMR spectrum of **3i**

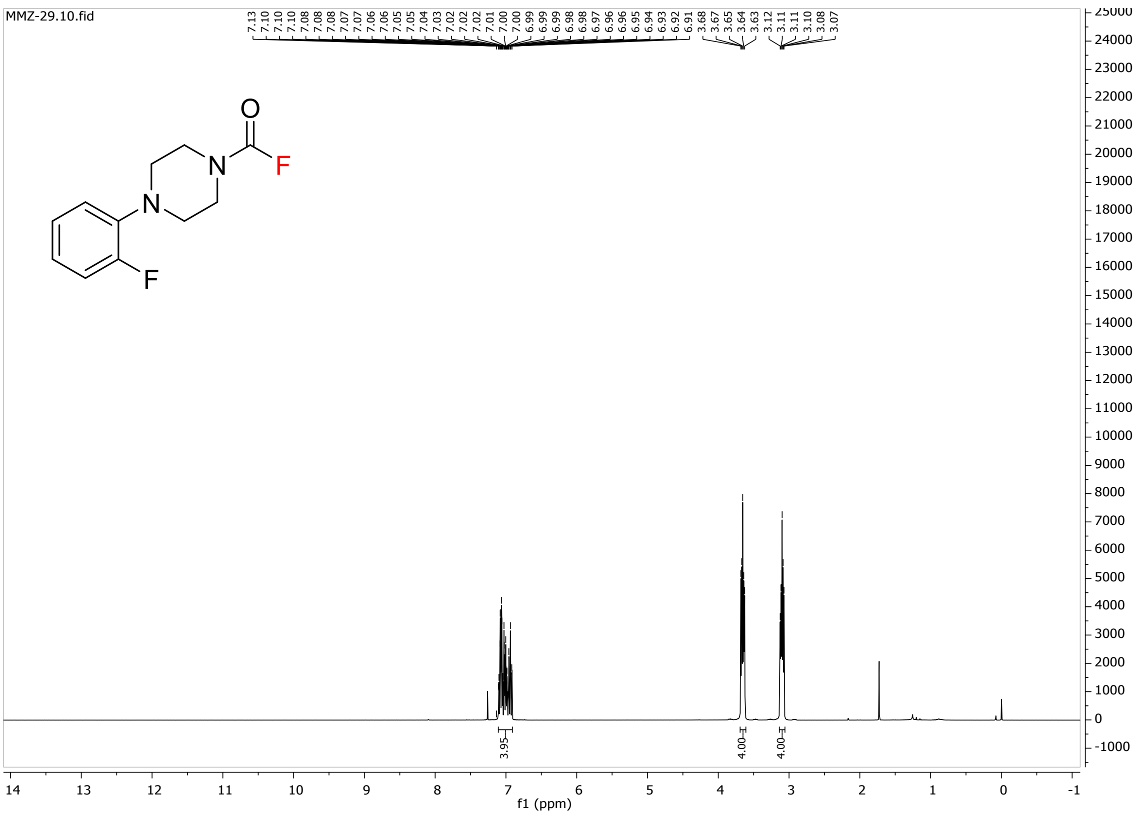

Figure S33. ^{1}H NMR spectrum of **3j**

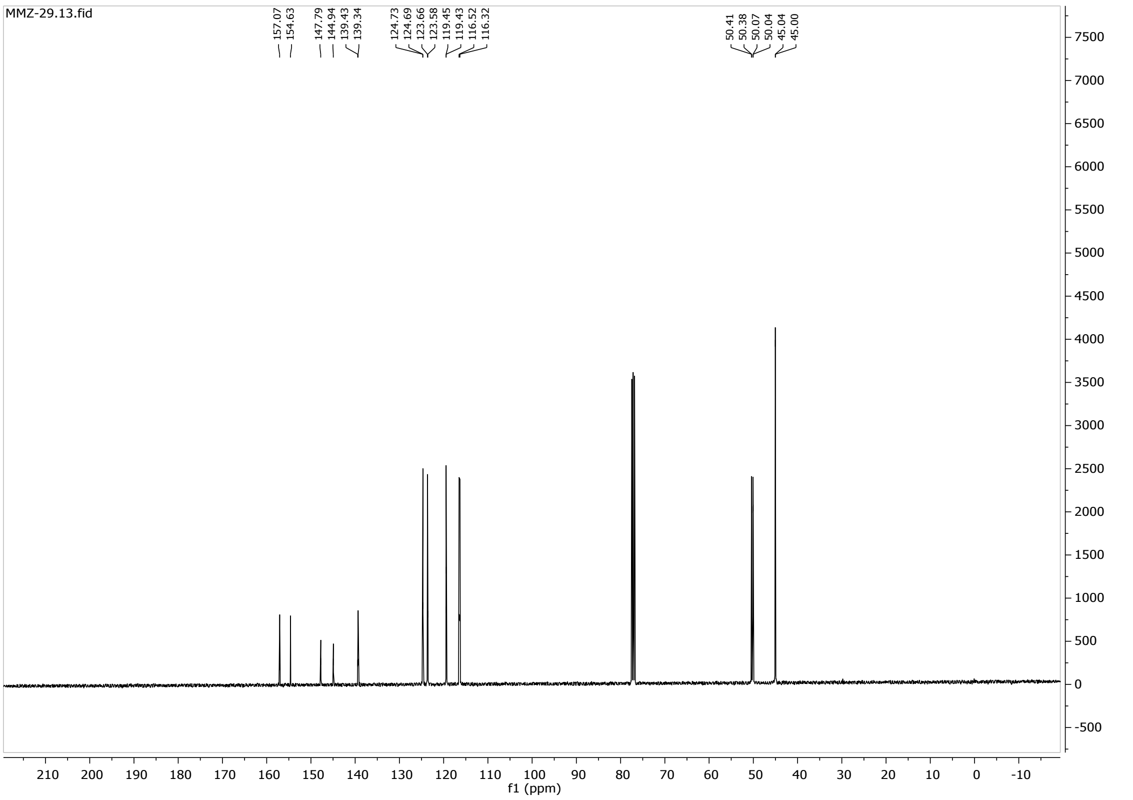

Figure S34. ^{13}C NMR spectrum of **3j**

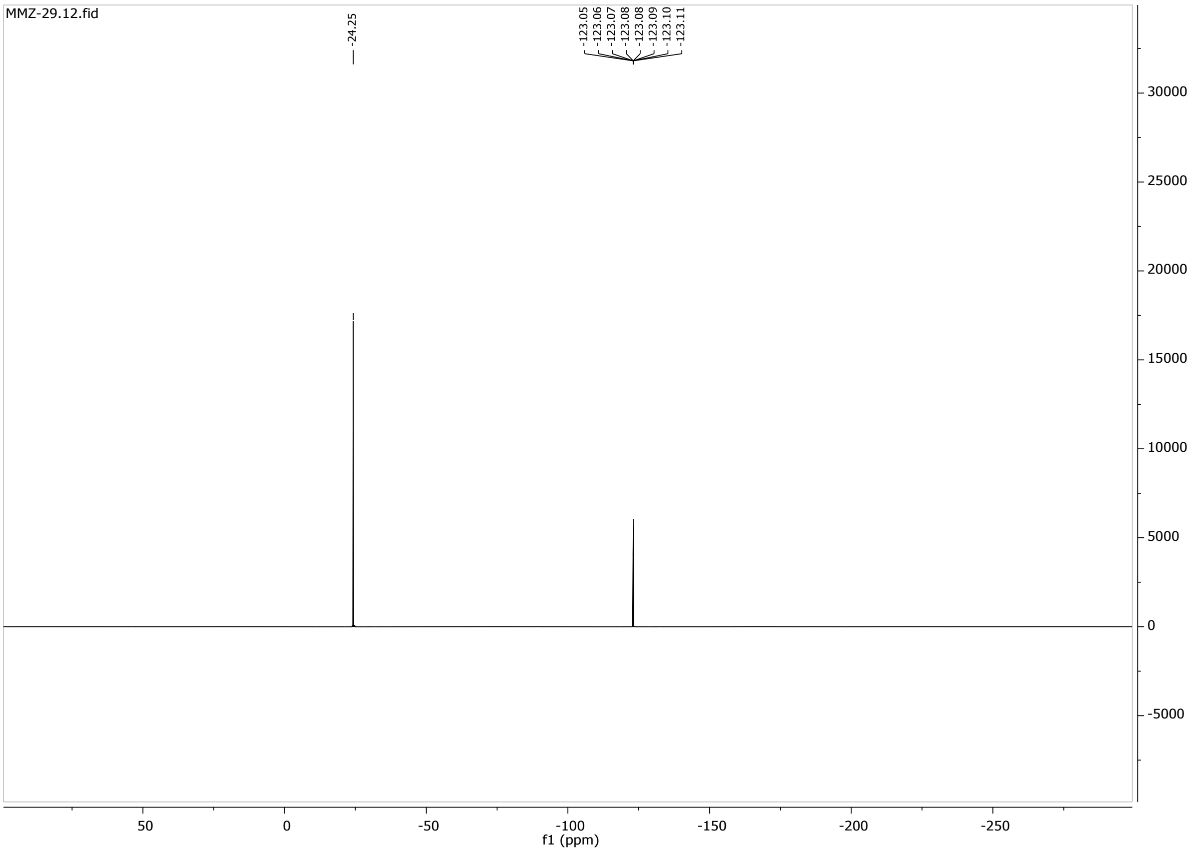

Figure S35. ^{19}F NMR spectrum of **3j**

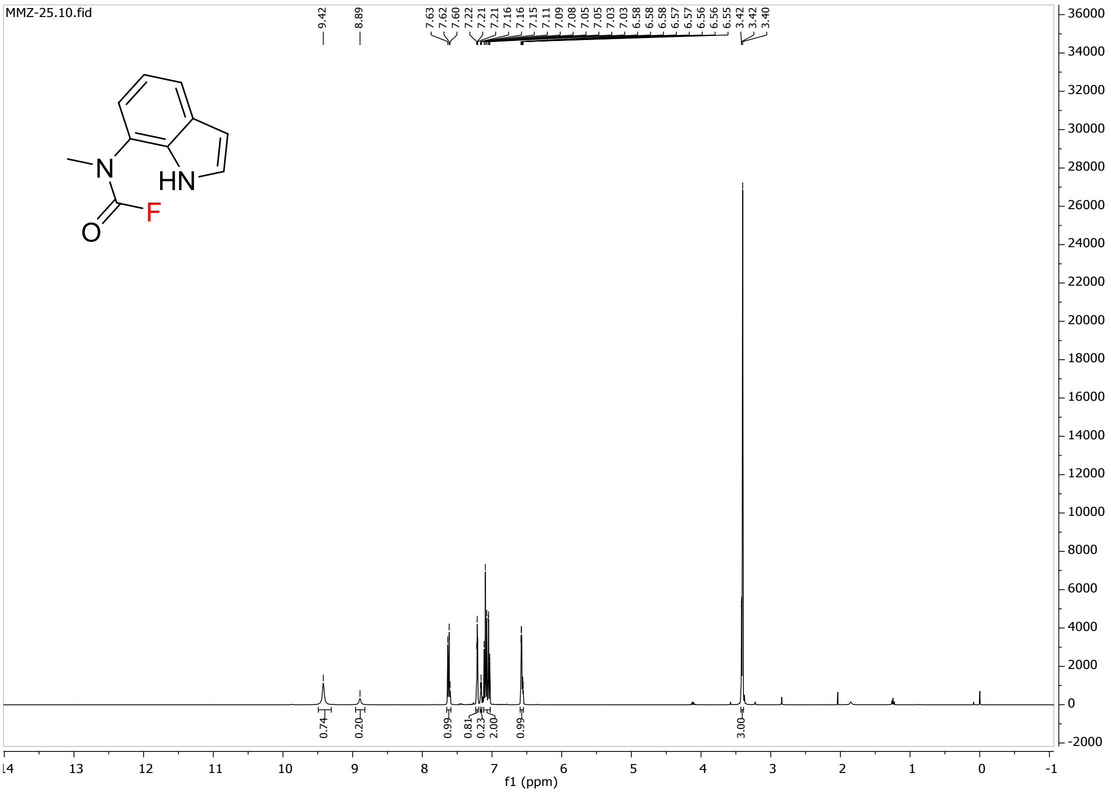

Figure S36. ^{1}H NMR spectrum of **3k**

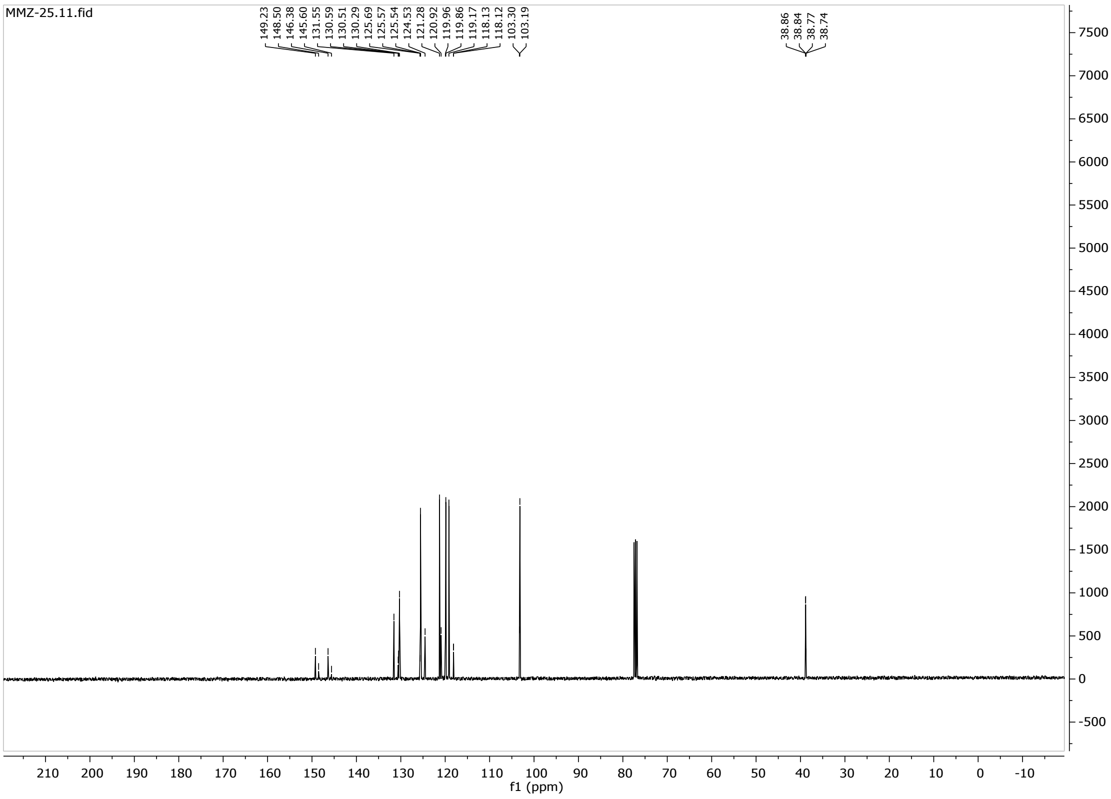

Figure S37. ^{13}C NMR spectrum of **3k**

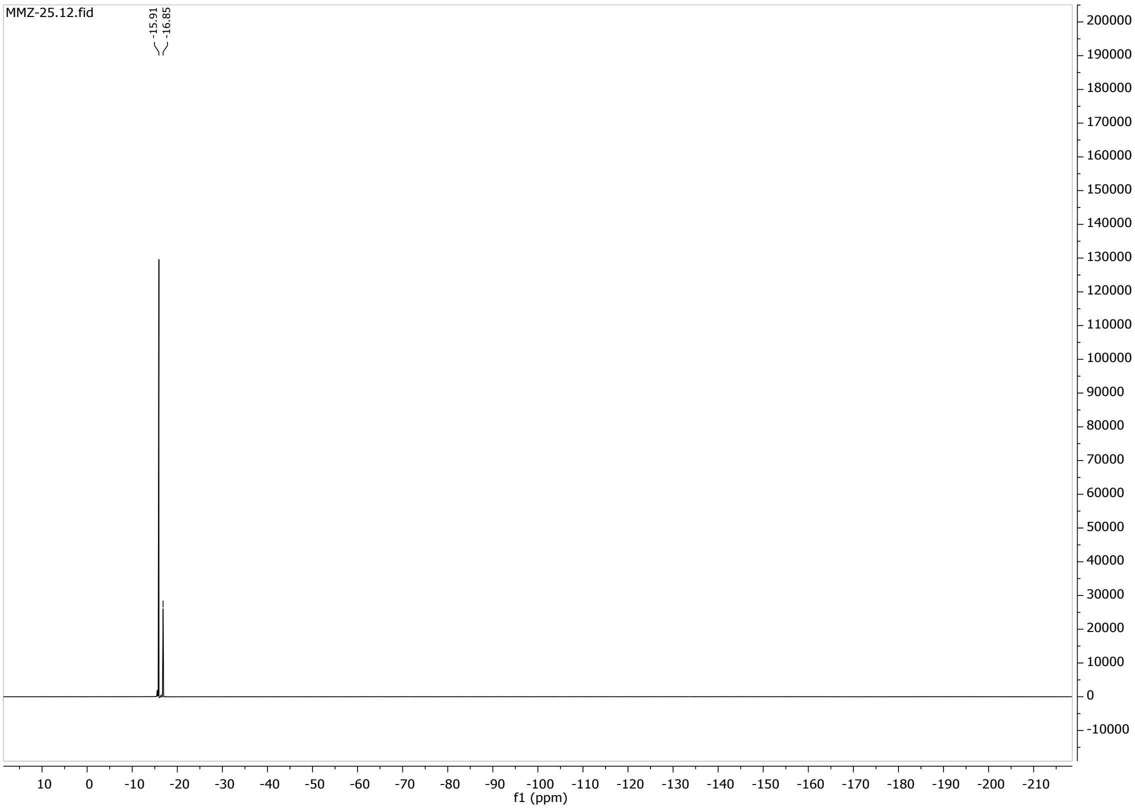

Figure S38. ^{19}F NMR spectrum of **3k**

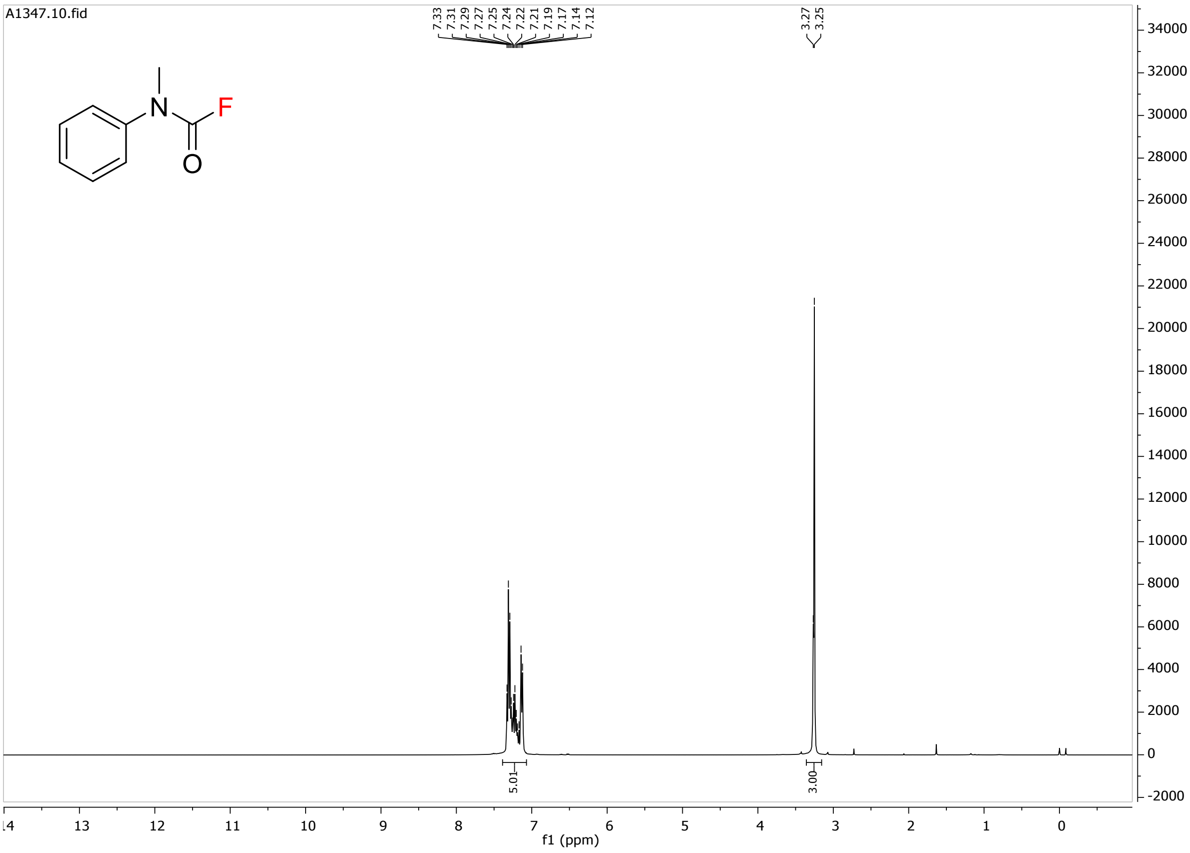

Figure S39. ^{1}H NMR spectrum of **31**

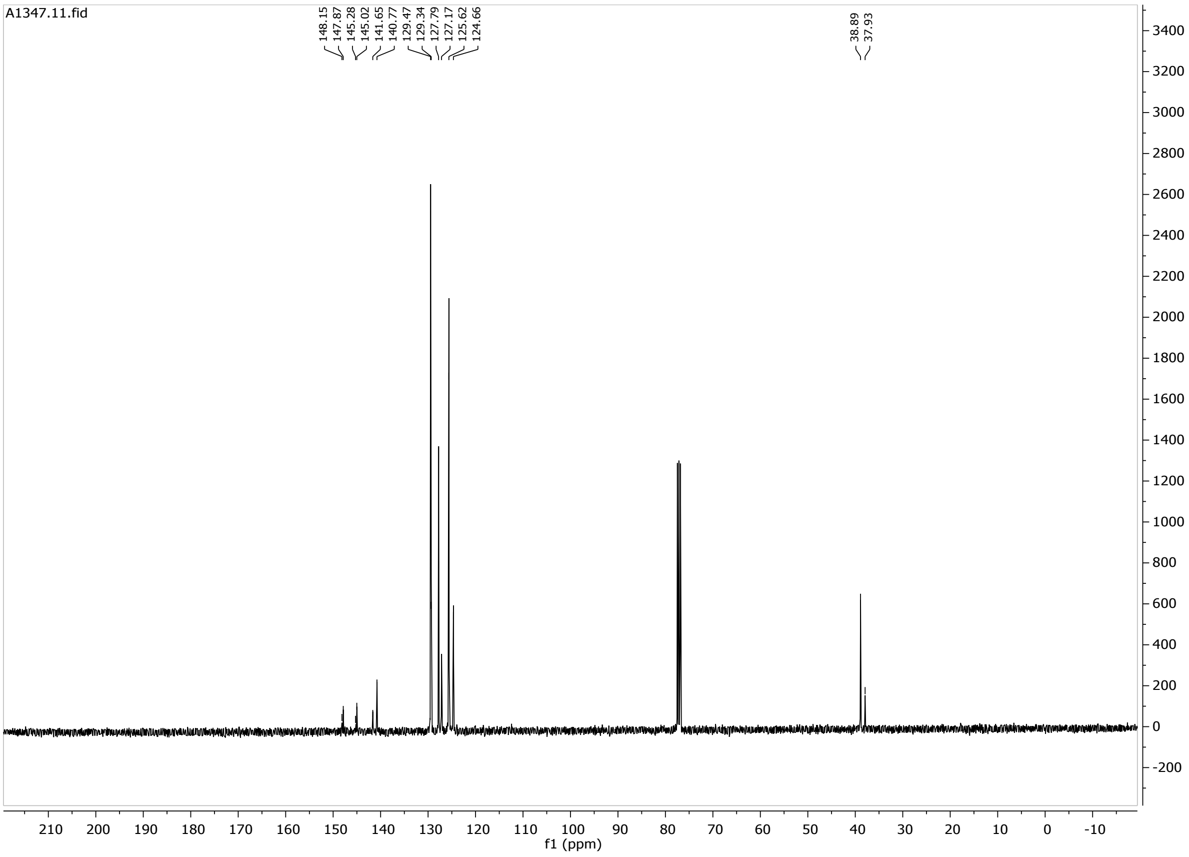

Figure S40. ^{13}C NMR spectrum of **31**

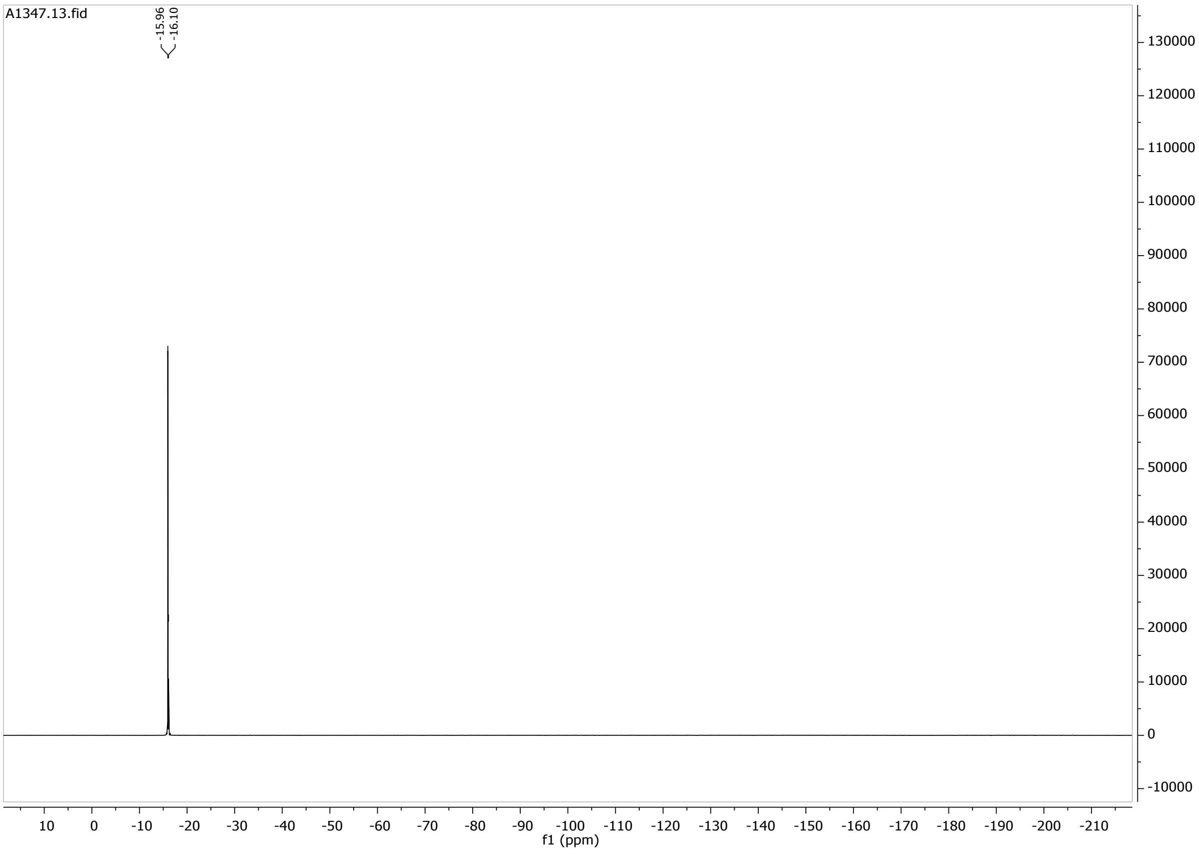

Figure S41. ^{19}F NMR spectrum of **3l**

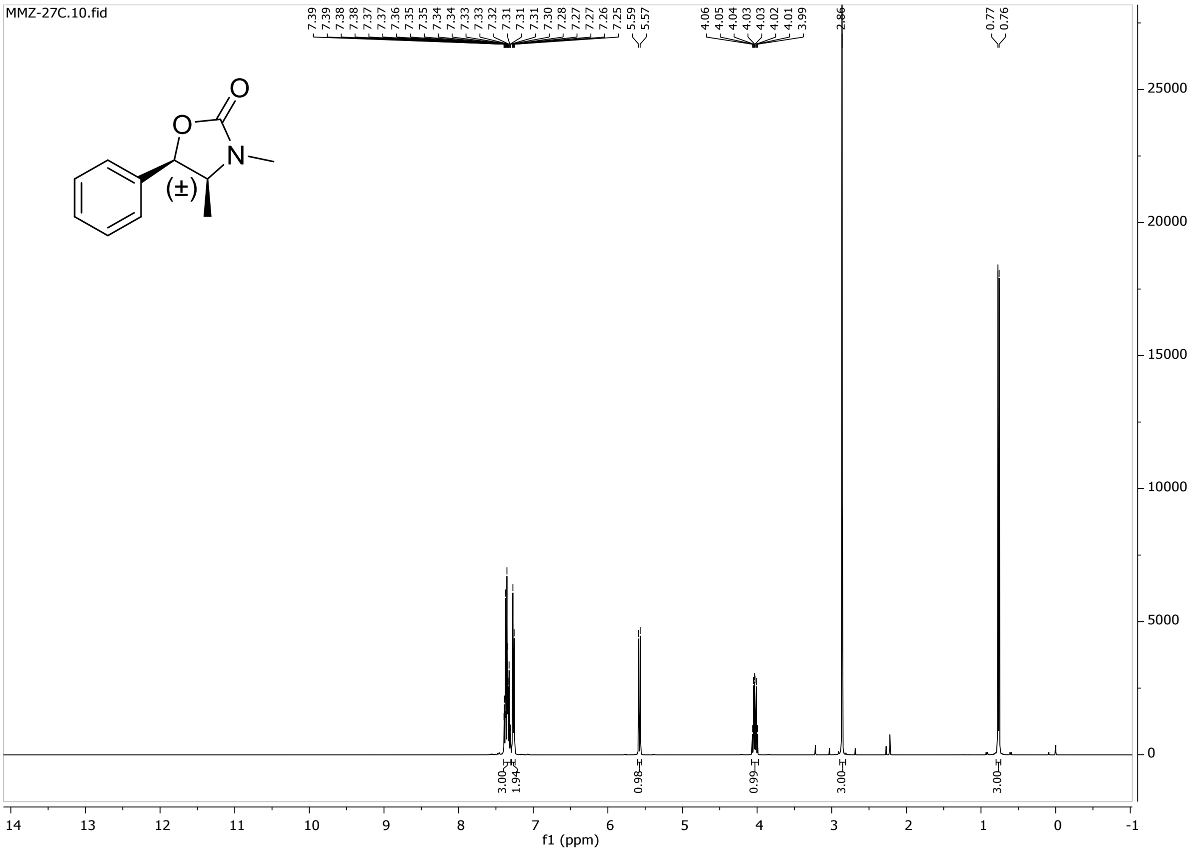

Figure S42. ^{1}H NMR spectrum of **3n**

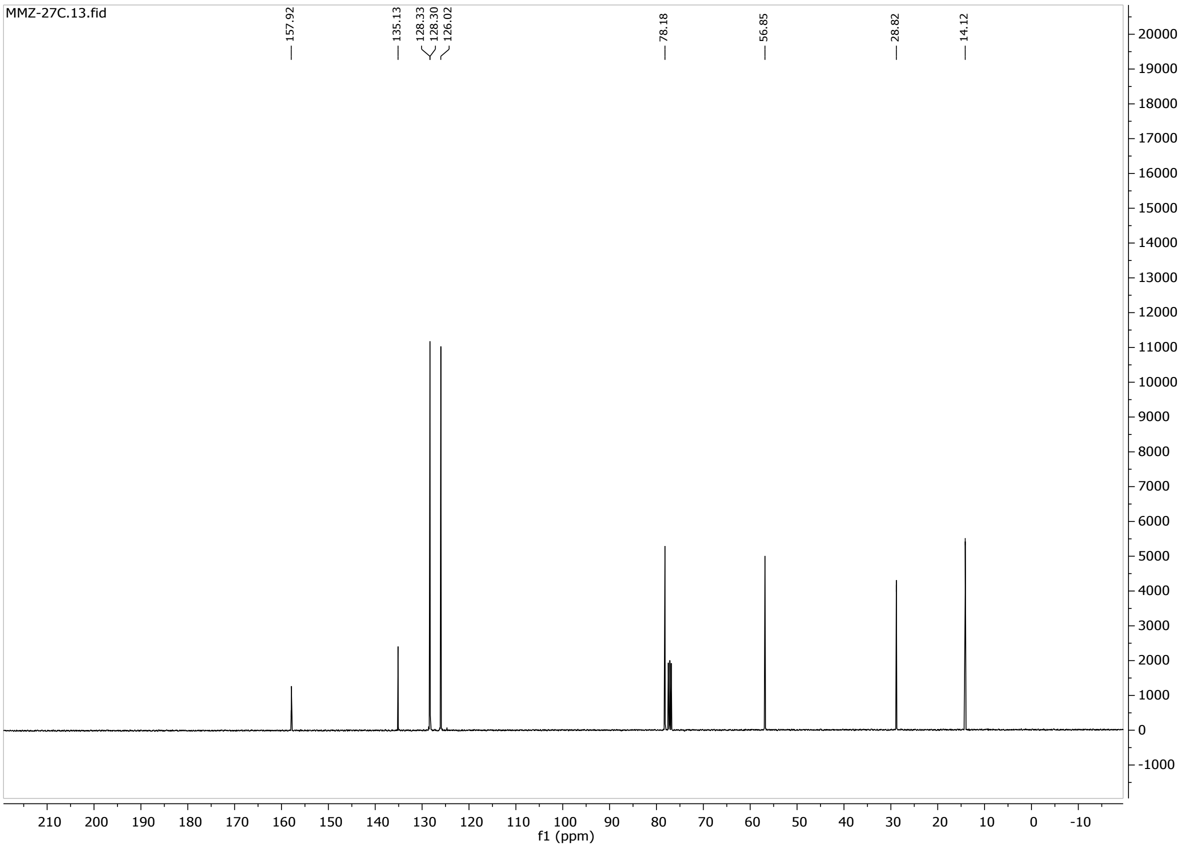

Figure S43. ^{13}C NMR spectrum of **3n**

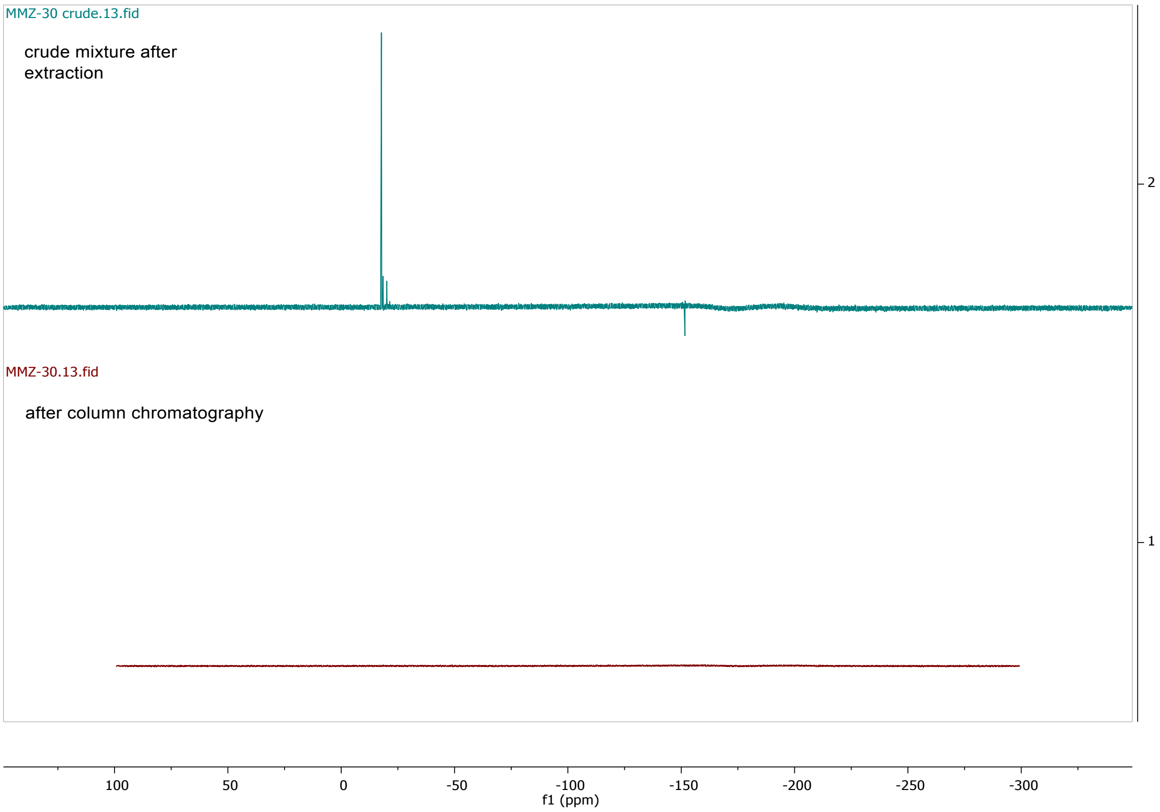

Figure S44. ^{19}F NMR spectrum of **3n**

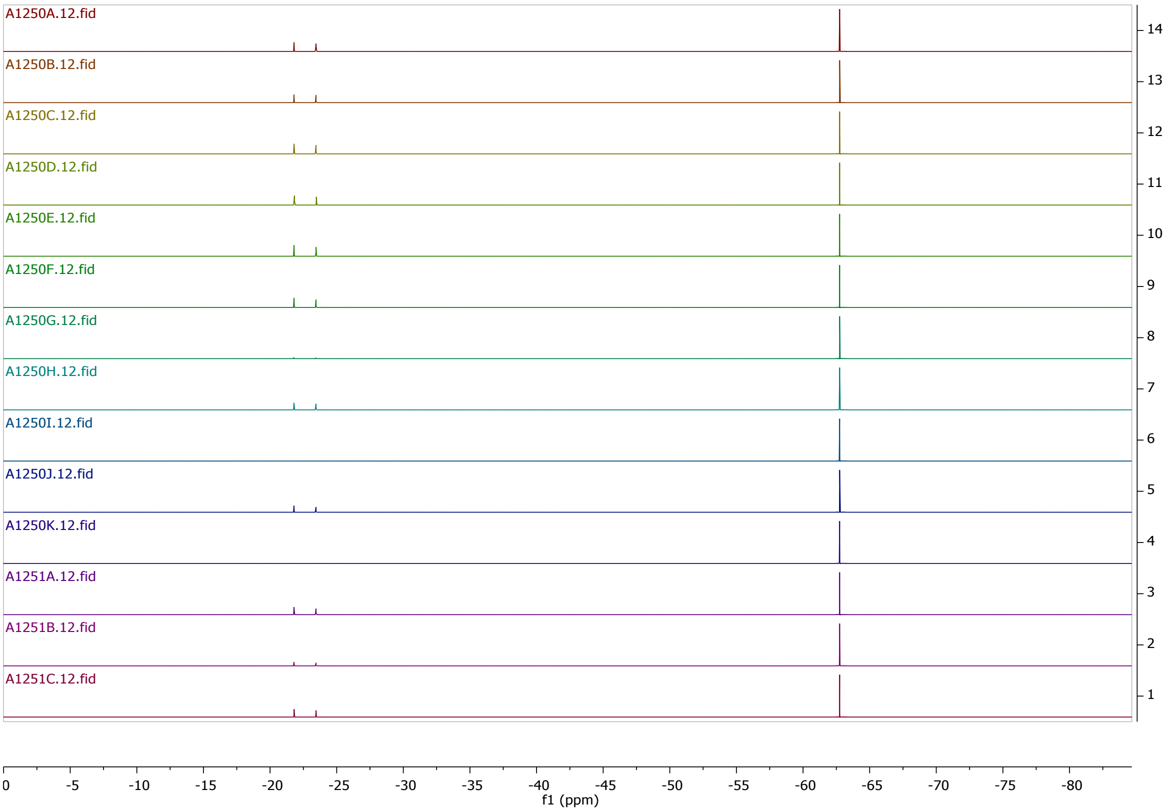

Figure S45. qNMR spectra for Table 1, entries 1–14

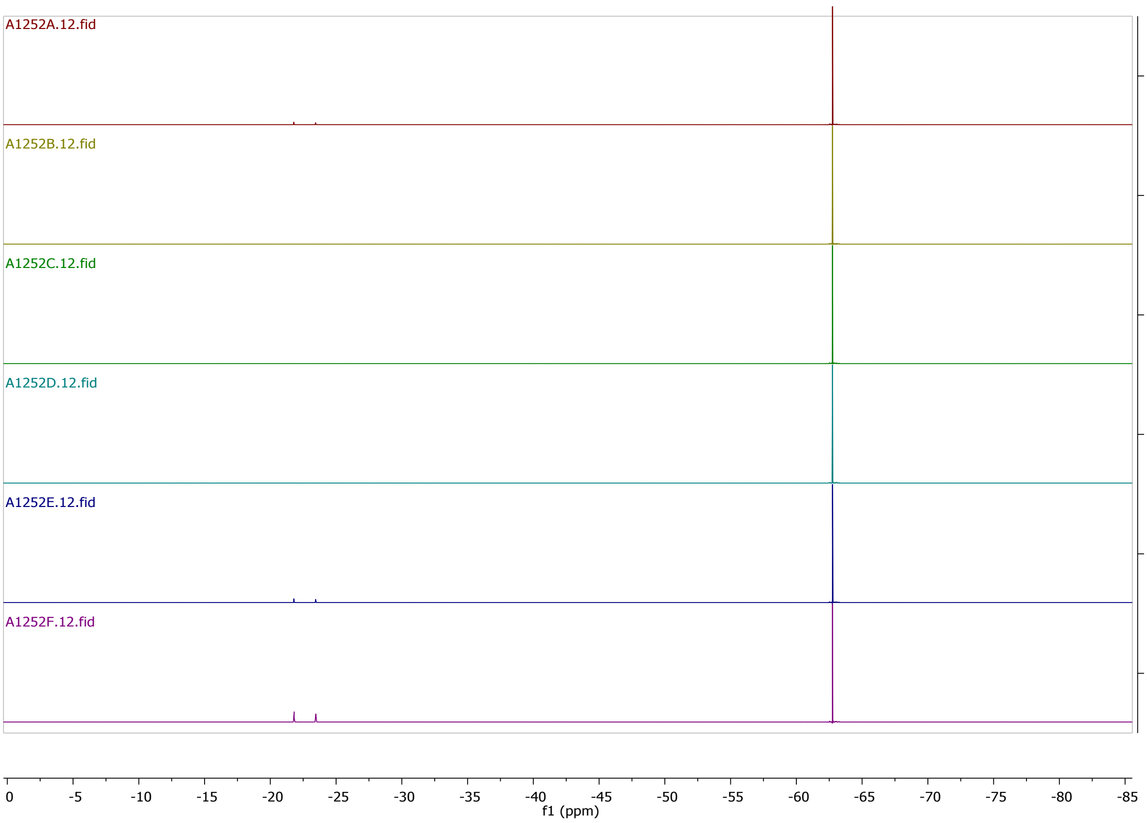

Figure S46. qNMR spectra for Table 1, entries 15–20

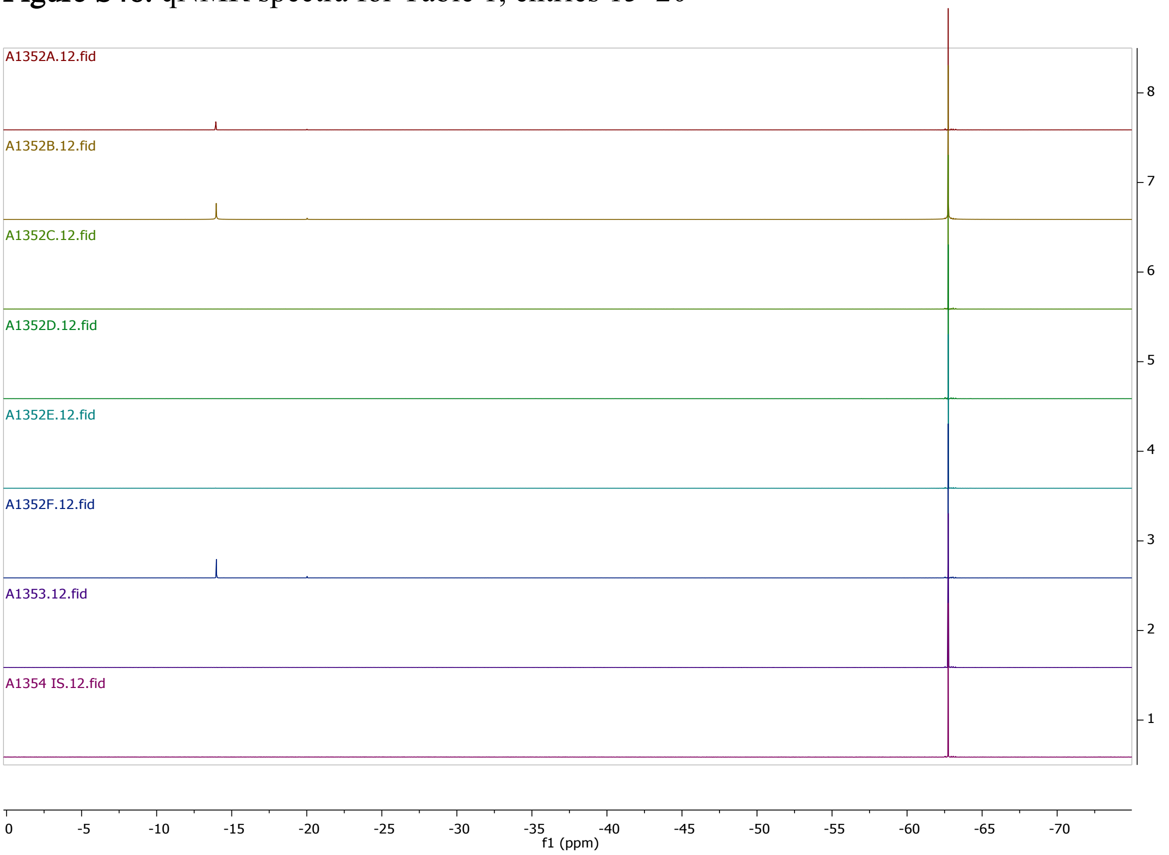

Figure S47. qNMR spectra for Table 1, entries 22–29

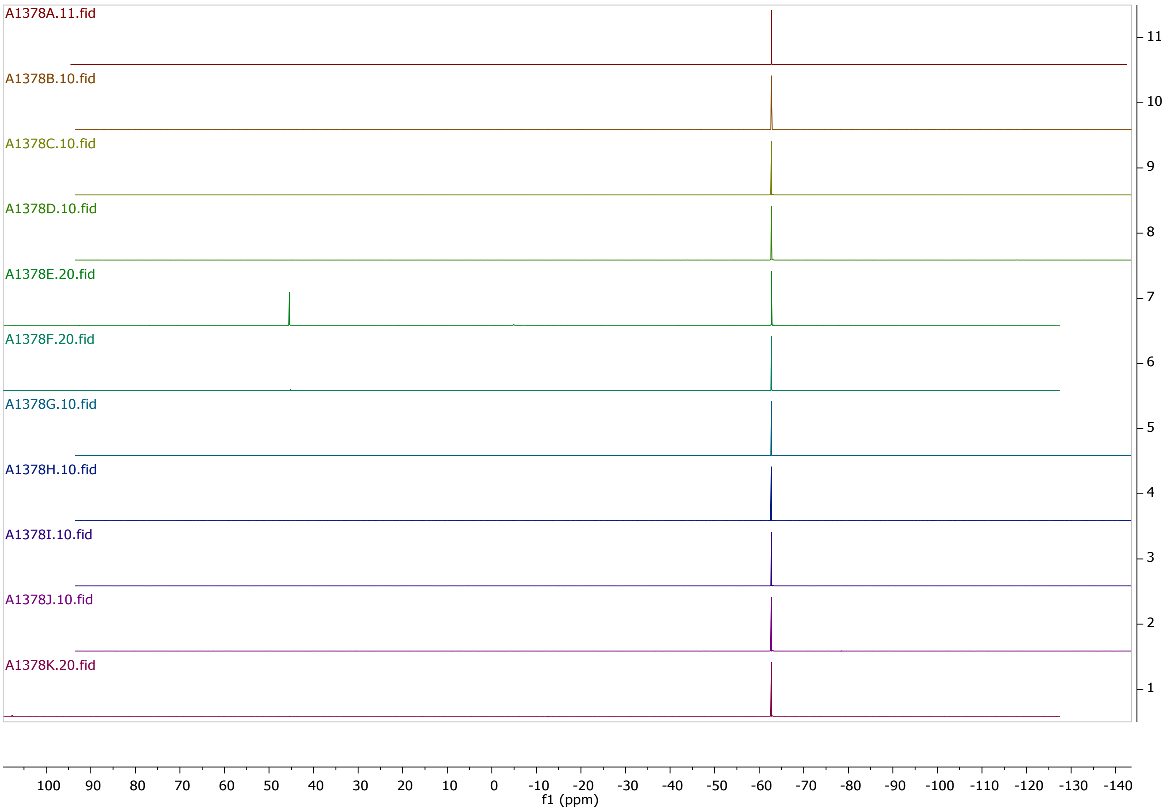

Figure S48. qNMR spectra for Table 2, entries 1–11

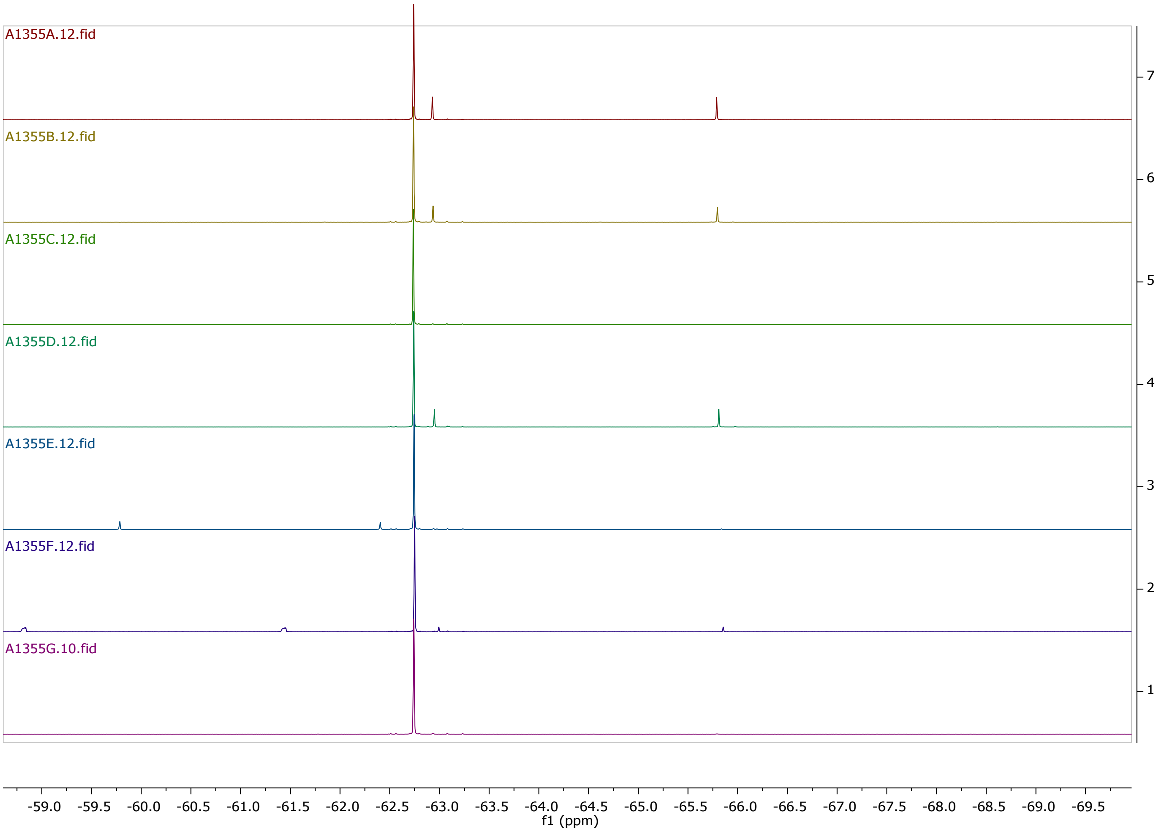

Figure S49. qNMR spectra for Table 2, entries 12–18

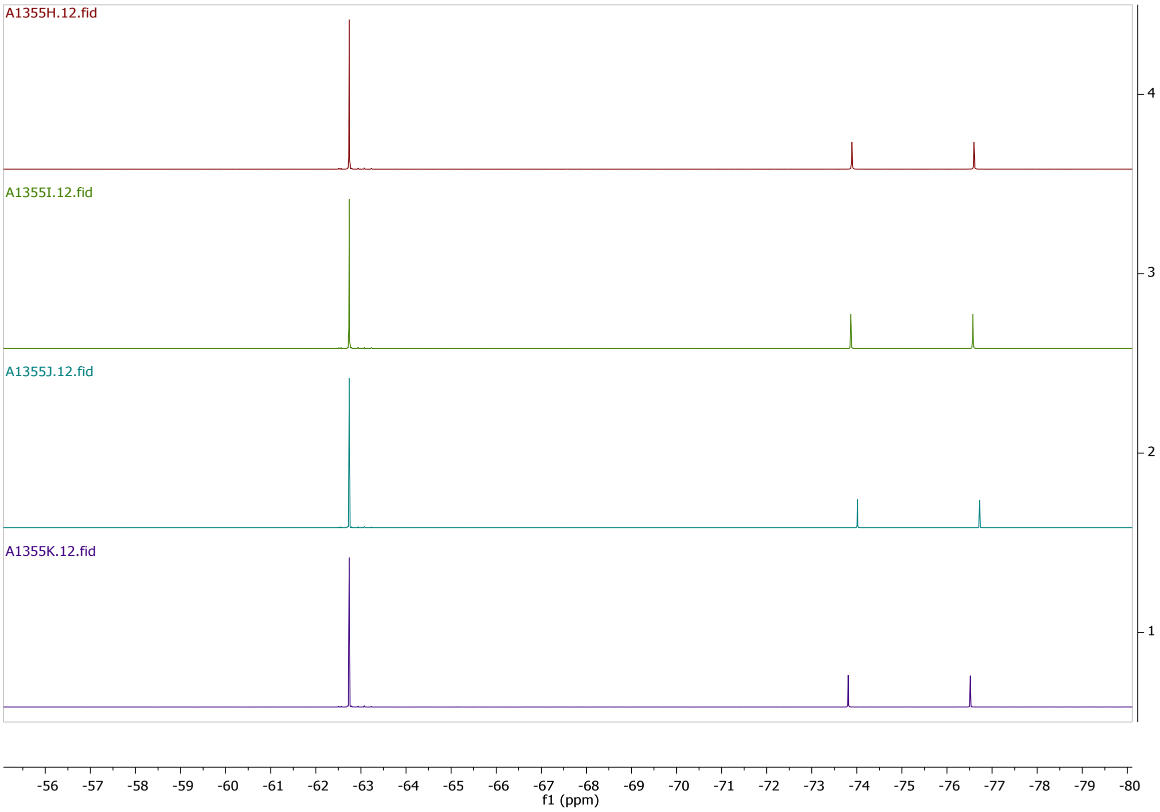

Figure S50. qNMR spectra for Table 2, entries 19–22

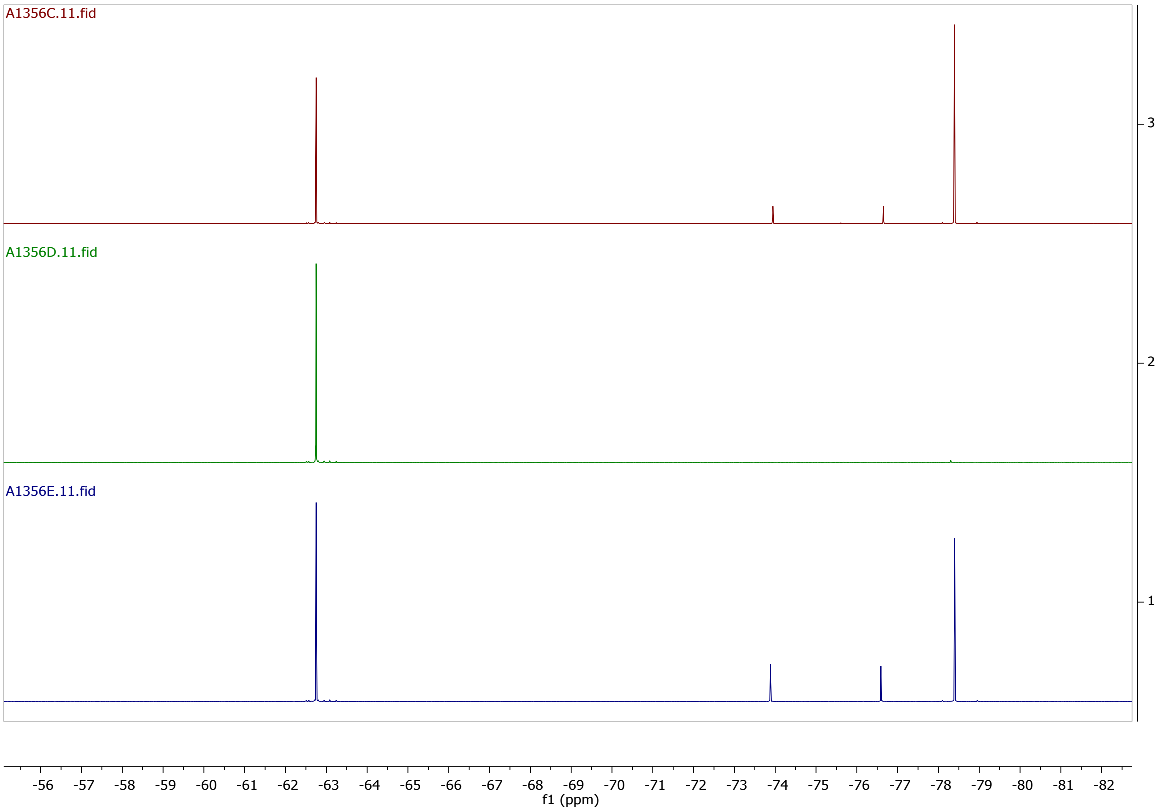

Figure S51. qNMR spectra for Table 2, entries 23–25

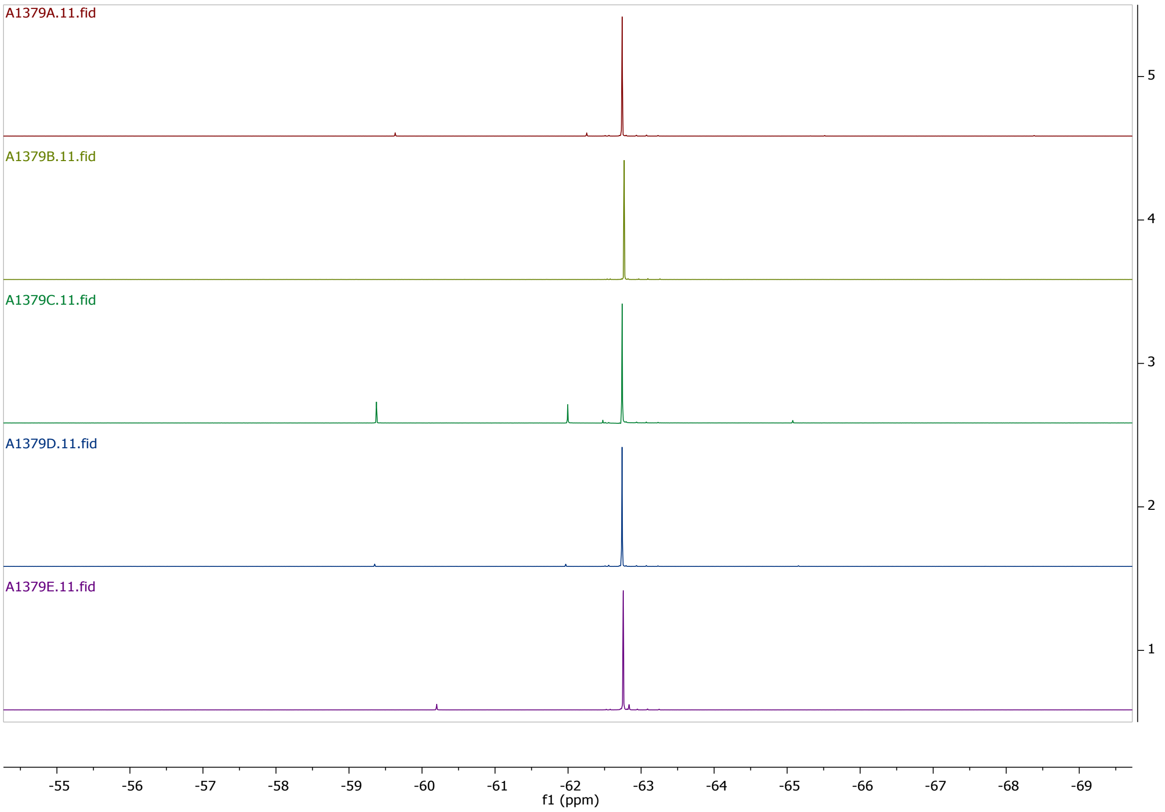

Figure S52. qNMR spectra for Table 2, entries 26–30

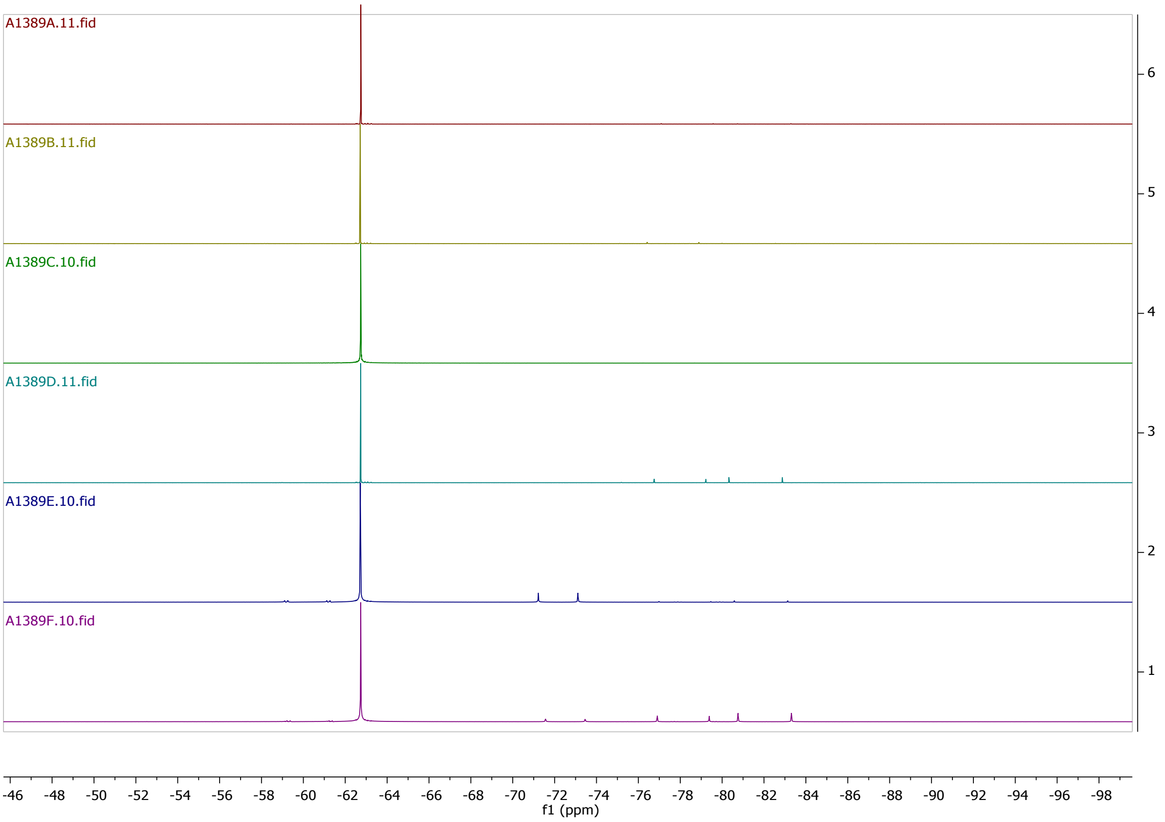

Figure S53. qNMR spectra for Table 2, entries 31–36
